# Clinical signature and associated immune metabolism of NLRP1 in pan‐cancer

**DOI:** 10.1111/jcmm.70100

**Published:** 2024-09-24

**Authors:** Yong Liao, Pinglian Yang, Cui Yang, Kai Zhuang, Aamir Fahira, Jiaojiao Wang, Zhiping Liu, Lin Yan, Zunnan Huang

**Affiliations:** ^1^ Key Laboratory of Computer‐Aided Drug Design of Dongguan City, The First Dongguan Affiliated Hospital Guangdong Medical University Dongguan Guangdong China; ^2^ Key Laboratory of Big Data Mining and Precision Drug Design of Guangdong Medical University, Key Laboratory for Research and Development of Natural Drugs of Guangdong Province, School of Pharmacy Guangdong Medical University Dongguan Guangdong China; ^3^ Guangdong Province Key Laboratory of Pharmacodynamic Constituents of TCM and New Drugs Research, College of Pharmacy Jinan University Guangzhou Guangdong China

**Keywords:** bioinformatics analysis, clinical signature, immune metabolism, NLRP1, pan‐cancer

## Abstract

Inflammations have been linked to tumours, suggesting a potential association between NLRP1 and cancer. Nevertheless, a systematic assessment of NLRP1's role across various cancer types currently absent. A comprehensive bioinformatic analysis was conducted to determine whether NLRP1 exhibits prognostic relevance linked to immune metabolism across various cancers. The study leveraged data from the TCGA and GTEx databases to explore the clinical significance, metabolic features, and immunological characteristics of NLRP1, employing various tools such as R, GEPIA, STRING and TISIDB. NLRP1 exhibited differential expression patterns across various cancers, with elevated expression correlating with a more favourable prognosis in lung adenocarcinoma (LUAD) and pancreatic adenocarcinoma (PAAD). Downregulation of NLRP1 reduced tumour metabolic activity in LUAD. Moreover, the mutational signature of NLRP1 was linked to a favourable prognosis. Interestingly, high NLRP1 expression inversely correlated with tumour stemness while positively correlating with tumour immune infiltration in various cancers including LUAD and PAAD. Through extensive big data analysis, we delved into the role of NLRP1 across various tumour types, constructing a comprehensive role map of its involvement in pan‐cancer scenarios. Our findings highlight the potential of NLRP1 as a promising therapeutic target specifically in LUAD and PAAD.

## 1 INTRODUCTION

According to the statistics from the World Health Organization (WHO) and the International Agency for Research on Cancer (IRAC), there were 20 million new cancer cases and 9.7 million cancer‐related deaths in 2022.^1^ The situation has gotten worse as the number of cancer patients has kept increasing over the past few years. Therefore, it is crucial to discover novel approaches for cancer prevention and treatment.

With the deepening of tumour immune research in recent years, it has been found that cell metabolism is a key factor in regulating the tumour immune microenvironment.^2^ Furthermore, nutrient consumption and metabolite production can affect the immune response, and the crosstalk among various cell types, comprising tumour cells and immune cells in a certain microenvironment also has a big influence on the tumour's growth and metastasis.^3^, ^4^ Inflammasomes are at the intersection of innate immune recognition and metabolic control.^5^

Nucleotide‐binding oligomerization domain (NOD)‐like receptor protein 1 (NLRP1), the first NOD‐like receptor (NLR) protein, serves as a pivotal inflammasome involved in both inflammation and innate immunity.^6^ NLRP1 plays a significant role in tumour immunity as a critical gene of cell pyroptosis.^7^, ^8^ NLRP1 may serve a critical innate immune regulator of cancers via promoting IL‐1β and IL‐18 secretion.^8^ NLRP1 also interacts with caspase‐1, which can initiate an immune response.^9^ NLRP1 can prevent obesity and metabolic syndrome through IL‐18.^10^ Furthermore, NLRP1 inhibits the development of intestinal tumours in animal models.^11^ It is therefore hypothesized that NLRP1 may serve as an effective clinical biomarker and have a substantial impact on both tumour immunity and metabolism.

**FIGURE 1:**
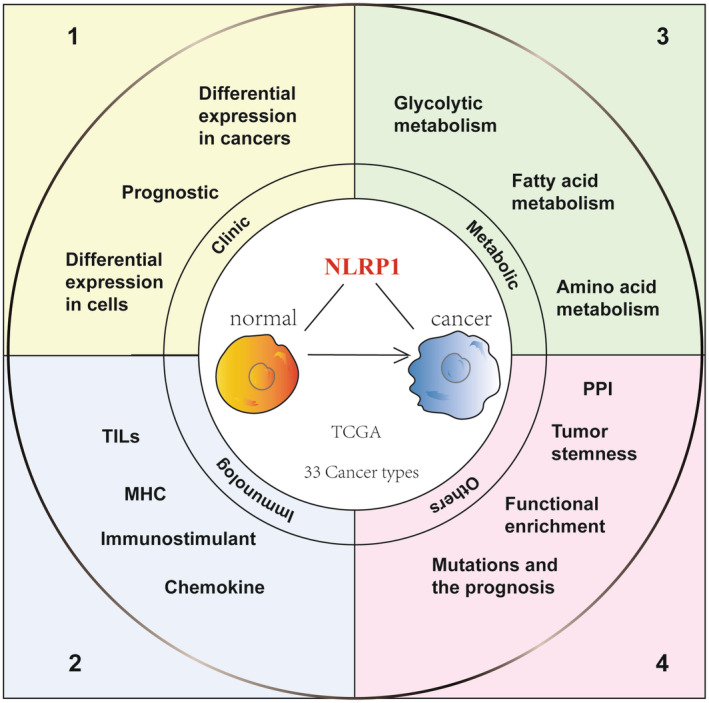
The flow chart of systematic pan‐cancer analysis of NLRP1. Thirty‐three cancer types from the TCGA database were utilized to analyse the functions of NLRP1 in clinical signature, tumour immunology, metabolism, stemness and mutation characteristics. MHC, major histocompatibility complex; NLRP1, nucleotide‐binding oligomerization domain‐like receptor protein 1; PPI, protein–protein interaction; TCGA, The Cancer Genome Atlas project; TILs, tumour‐infiltrating lymphocytes.

Until now, only a limited number of studies have investigated the role and function of NLRP1 in cancer studies, most of which are limited to a single type of cancer and have not studied the relationship between NLRP1 and tumour immunity or metabolism. Therefore, this study delves into the significant role of NLRP1 in cancer, examining its clinical significance, tumour immune characteristics, tumour metabolic characteristics, tumour stemness, mutation effect, protein interaction network and functional pathway enrichment analysis based on pan‐cancer analysis. (Figure 1 provides an overview of the study design, while Figure S1 presents a detailed study design tree diagram.) Studying the role of NLRP1 in tumorigenesis holds immense significance as it aids in enhancing our comprehension of the molecular mechanisms underlying tumorigenesis. This comprehension serves as a pivotal scientific groundwork for devising potent and safe anti‐tumour drugs. Moreover, it empowers researchers to delve deeper into the complexities of tumorigenesis, ultimately fostering the development of more effective tumour treatment strategies.

## 2 MATERIALS AND METHODS

### 2.1 Data collection and analysis

Sangbox 3.0 webserver (http://vip.sangerbox.com/)^12^ were preconfigured with the uniformly standardized data from UCSC database (Genome Browser, University of California, Santa Cruz, http://genome.ucsc.edu/)^13^ which integrated the pan‐cancer data and partial normal sample data from The Cancer Genome Atlas (TCGA, https://portal.gdc.cancer.gov/)^14^ and major normal sample data from Genotypic Tissue Expression (GTEx. https://gtexportal.org/home/)^15^ together. Most of the pan‐cancer analyses in this study were based on predefined gene expression data in Sangerbox,^12^ and the samples are shown in Table S1. Specifically, the NLRP1 expression files for each sample, along with relevant clinical parameters such as age, gender and survival status, were retrieved from these databases.

Solid normal tissues, primary blood samples derived from cancer patients and primary tumour tissues were screened. The expression values were transformed by Log_2_ (*x* + 0.001) for any analysis. Cancer types with less than three samples were excluded from subsequent analysis. Three external validation datasets (GSE10072, GSE15471 and GSE53757) were acquired from the Gene Expression Omnibus (GEO, https://www.ncbi.nlm.nih.gov/geo/) database. Dataset GSE10072 included 58 LUAD samples and 49 normal samples. Dataset GSE15471 included with 39 PAAD samples and 39 normal samples. Dataset GSE53757 included 72 KIRC samples and 72 normal samples. R (version 3.14.3) was used for statistical analysis. Data from two comparison groups were analysed using the Wilcoxon rank‐sum test if not otherwise stated, and *p* < 0.05 was considered statistically significant.

### 2.2 Expression pattern analysis of NLRP1 in tissues and cells

The Human Protein Atlas (HPA, https://www.proteinatlas.org) database^16^, ^17^ was used to describe the expression pattern of NLRP1 in various tissues and cell lines. In HPA, protein evidence scores for genes were calculated based on UniProt protein existence (UniProt evidence); neXtProt protein existence (neXtProt evidence); and a Human Protein Atlas antibody‐ or RNA‐based score (HPA evidence).^16^, ^17^

### 2.3 Differential expression analysis of NLRP1 in pan‐cancer

The Sangerbox 3.0 webserver was used to calculate differences in gene expression between normal and tumour samples in pan‐cancer. Additionally, box plots were created to contrast the discrepancies in NLRP1 expression between tumour and normal samples for LUAD, PAAD and KIRC using external validation datasets. The unmatched Wilcoxon rank sum test was used to determine the statistical significance of differential gene expression, with a threshold *p* value of <0.05.

### 2.4 Correlation analysis between NLRP1 and tumour prognosis

The Kaplan Meier plotter (http://kmplot.com/)^18^ was utilized to determine the significance between NLRP1 gene expression and pan‐cancer prognosis. Pan‐cancer module with server's default parameter was employed to categorize patients into the high‐ and low‐expression groups. Optimal cutoff values were determined according to the best‐performing threshold via calculating all possible thresholds between the lowest and highest quartiles.^19^ Patients surviving beyond the chosen threshold were censored. Median survival was calculated and visualized by Kaplan–Meier survival plots.

The Cox proportional hazards regression model was built to evaluate the prognostic significance of NLRP1 expression on both overall survival (OS) and disease‐specific survival (DSS) in each tumour type by using the coxph function of the R package survival (version 3.2‐7) in Sangerbox 3.0 webserver. The log‐rank test was utilized to measure the prognostic significance with a threshold *p* value of <0.05.

### 2.5 Comparison analysis of NLRP1 differential expression across single‐cell types

To explore NLRP1 expression across various tumour types at the single‐cell level, we conducted gene expression analysis using the deconvolution tool EPIC in the Gene Expression Profiling Interactive Analysis (GEPIA2021) database (http://gepia2021.cancer‐pku.cn/).^20^ In this analysis, we selected the interactive option in the Cell Type Level Expression Analysis module to display boxplots based on cell type, enabling simultaneous visualization and comparison of gene expression between normal and tumour tissues across specified cell types. The statistical significance of the expressional difference in NLRP1 was assessed using the one‐way ANOVA method^21^ with a *p*‐value <0.05 indicating statistical significance.

### 2.6 Protein–protein interaction (PPI) network and enrichment analysis of NLRP1

STRING (https://cn.string‐db.org/), a PPI database, was employed to conduct the PPI network analysis (minimum required interaction score: 0.400) of NLRP1.^22^ NLRP1 and its interaction genes from the PPI network were further used for enrichment analysis using the Kyoto Encyclopedia of Genes and Genomes (KEGG)^23^ and Gene Ontology (GO),^24^ respectively. Briefly, The KEGG and GO analyses were conducted in R software using ‘clusterProfiler’ package (version 3.1.0)^25^ which used the ‘org.Hs.eg.db’ (version 3.1.0) for the gene ID consistent conversions.^25^, ^26^ The pathways and functions were enriched with a minimum gene number of 5 from the selected interaction genes. The Benjamini–Hochberg method was employed to ascertain the false discovery rate (FDR) for correcting *p*‐values in multiple comparisons. A statistically significant threshold was set at an FDR value less than 0.05.

### 2.7 Correlation analysis between NLRP1 expression and tumour stemness

To explore the relationship between NLRP1 expression and tumour stemness, we obtained stemness scores based on the mRNA expression (RNAss) of each tumour sample following a previous study.^27^ Pearson's correlation coefficient was calculated between RNAss and NLRP1 expression of the tumour sample with a *p*‐value <0.05 indicating statistical significance. The lollipop chart was used to visualize the correlation results.

### 2.8 Immune microenvironment analysis of NLRP1

The Tumour Immune System Interactions Database (TISIDB) (http://cis.hku.hk/TISIDB/index.php),^28^ an integrated repository portal, provides the categories of tumour‐infiltrating lymphocytes (TILs), three types of immune‐related genes (including immunostimulatory factors, major histocompatibility complex MHC molecules and chemokine proteins) and immune subtypes. According to the NLRP1 expression profile, Gene‐Set Variation Analysis (GSVA),^29^ which is used to assess the enrichment level of gene sets, was conducted in TISIDB to determine the relative abundance of TILs and immune‐related genes in pan‐cancer. Spearman correlations were further employed to assess the correlation between NLRP1 and the expression of 28 TILs, 45 immunostimulatory factors, 21 MHC molecules and 41 chemokine proteins and visualized using heatmaps. In addition, the Kruskal–Wallis test^30^ was employed to assess the statistical significance of NLRP1 expression variations across different immune subtypes in each cancer and also to show the comparison of the expression variations among distinct immune subtypes in five top subtype‐varied cancers, followed by visualization using barplots and violinplots, respectively. The correlation was estimated by the Spearman test with a *p*‐value <0.05 indicating statistical significance.

### 2.9 Gene set enrichment analysis (GSEA) of NLRP1

To elucidate the biological functions associated with the expression of NLRP1 across 33 cancer types, the ‘clusterProfiler’ package was utilized for GSEA.^31^ The hallmark gene sets were acquired from the Molecular Signatures Database (MSigDB) using the file ‘h.all.v7.2.symbols.gmt’.^32^ Cohorts with high and low expressions of NLRP1 were distinguished based on the top and bottom 30% thresholds for each cancer type.^32^ Then, potential biological pathways based on hallmark pathway‐related gene sets in the high and low expression groups of NLRP1 were enriched in each tumour. Pathways with an FDR of less than 0.05 and an absolute Normalized Enrichment Score (NES)^31^ greater than 1 were considered to indicate potential biological functions linked to NLRP1.

### 2.10 Correlation analysis between NLRP1 and tumour metabolism

Gene sets of glycolysis metabolism, fatty acid metabolism and amino acid metabolism were downloaded from MSigDB.^32^ For LUAD, Gene Expression Profiling Interactive Analysis2 (GEPIA2) (http://gepia2.cancer‐pku.cn/)^33^ was used to analyse the relationship between NLRP1 and these metabolisms. Pearson's correlation analysis was employed to assess their correlation coefficients, in which the non‐log scale was used for calculation and the log scale was used for visualization.^33^

### 2.11 Correlation analysis between NLRP1 mutations and tumour prognosis

A mutation frequency analysis of NLRP1 in various tumour types was conducted utilizing the cBioPortal database (http://www.cbioportal.org/).^34^ Concisely, the NLRP1 mutations in tumours and a possible relationship between genetic alteration in NLRP1 and survival prognosis were investigated. The Kaplan–Meier plots were carried out to compare prognostic disparities among the altered and unaltered groups within NLRP1 mutations. The log‐rank test was used to assess the statistical significance with a *p‐*value less than 0.05 indicating statistical significance.

## 3 RESULTS

### 3.1 NLRP1 different expression pattern in tissues and cells

**FIGURE 2:**
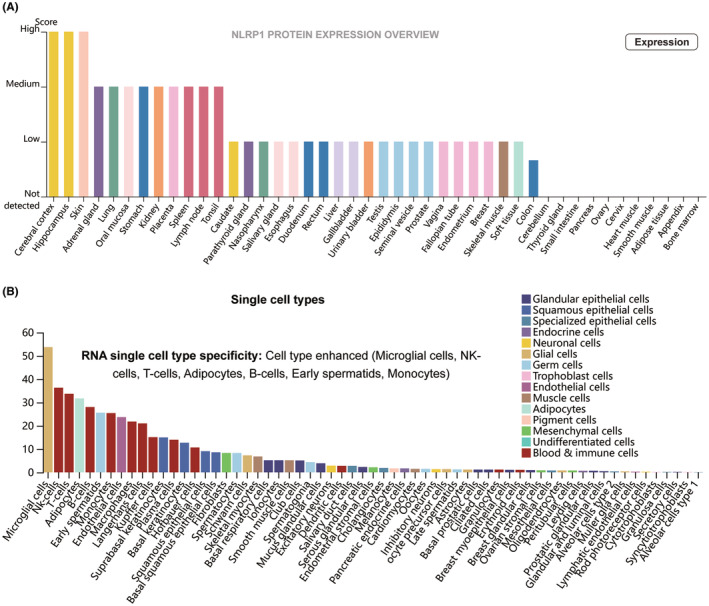
The distinctive expression pattern of NLRP1. (A) Protein expression levels of NLRP1 in different normal tissues. (B) Expression of NLRP1 in different types of cells.

NLRP1 expression pattern in different type of tissues and cells was analysed via the HPA database. As depicted by Figure 2A, the NLRP1 is highly expressed in the cerebral cortex, hippocampus, skin, adrenal gland, lung, oral mucosa, stomach, kidney, placenta, spleen, lymph node and tonsil. NLRP1 is the most highly expressed in the cerebral cortex and hippocampus among these tissues. NLRP1 expression has RNA single‐cell type specificity (Figure 2B). It is highly expressed in microglial cells, NK‐cells, T cells, adipocytes, B cells, early spermatids and monocytes, among which the expression is highest in microglial cells. These results indicate that NLRP1 exhibits distinct expression patterns across various types of tissues and cells.

### 3.2 NLRP1 differential expression in pan‐cancer

**FIGURE 3:**
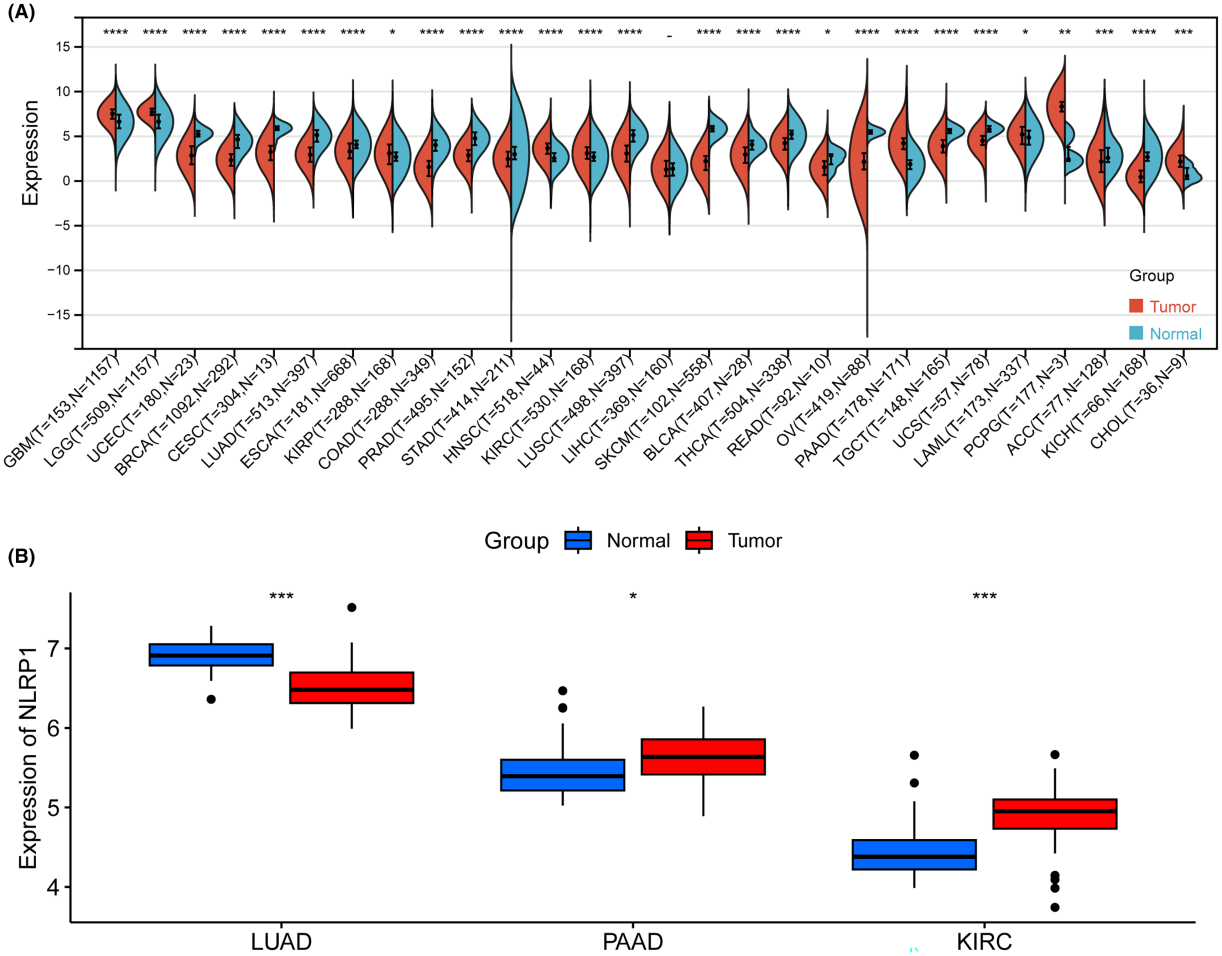
Differential expression of NLRP1 in pan‐cancer. (A) Expression of NLRP1 in multiple cancer tissues and the adjacent normal tissues. (B) The external validation of NLRP1 abnormal expression in LUAD, PAAD and KIRC. Red indicates tumour samples, while blue represents normal samples. *p < 0.05, **p < 0.01, ***p < 0.001, and ****p < 0.0001.

NLRP1 differential expression analysis was conducted in the Sangerbox 3.0 webserver to investigate the expression of NLRP1 in pan‐cancer. NLRP1 is significantly upregulated in 9 types of tumours including (GBM, LGG, KIRP, HNSC, KIRC, PAAD, LAML, PCPG, CHOL), while downregulated in 18 types of tumours containing (UCEC, BRCA, CESC, LUAD, ESCA, COAD, PRAD, STAD, LUSC, SKCM, BLCA, THCA, READ, OV, TGCT, UCS, ACC, KICH) (Figure 3A). Furthermore, external validation on three selected cancers confirms that NLRP1 is downregulated in LUAD, while upregulated in PAAD and KIRC (Figure 3B). These results suggest that NLRP1 is differentially expressed in pan‐cancer.

### 3.3 Correlation between NLRP1 and tumour prognosis

**FIGURE 4:**
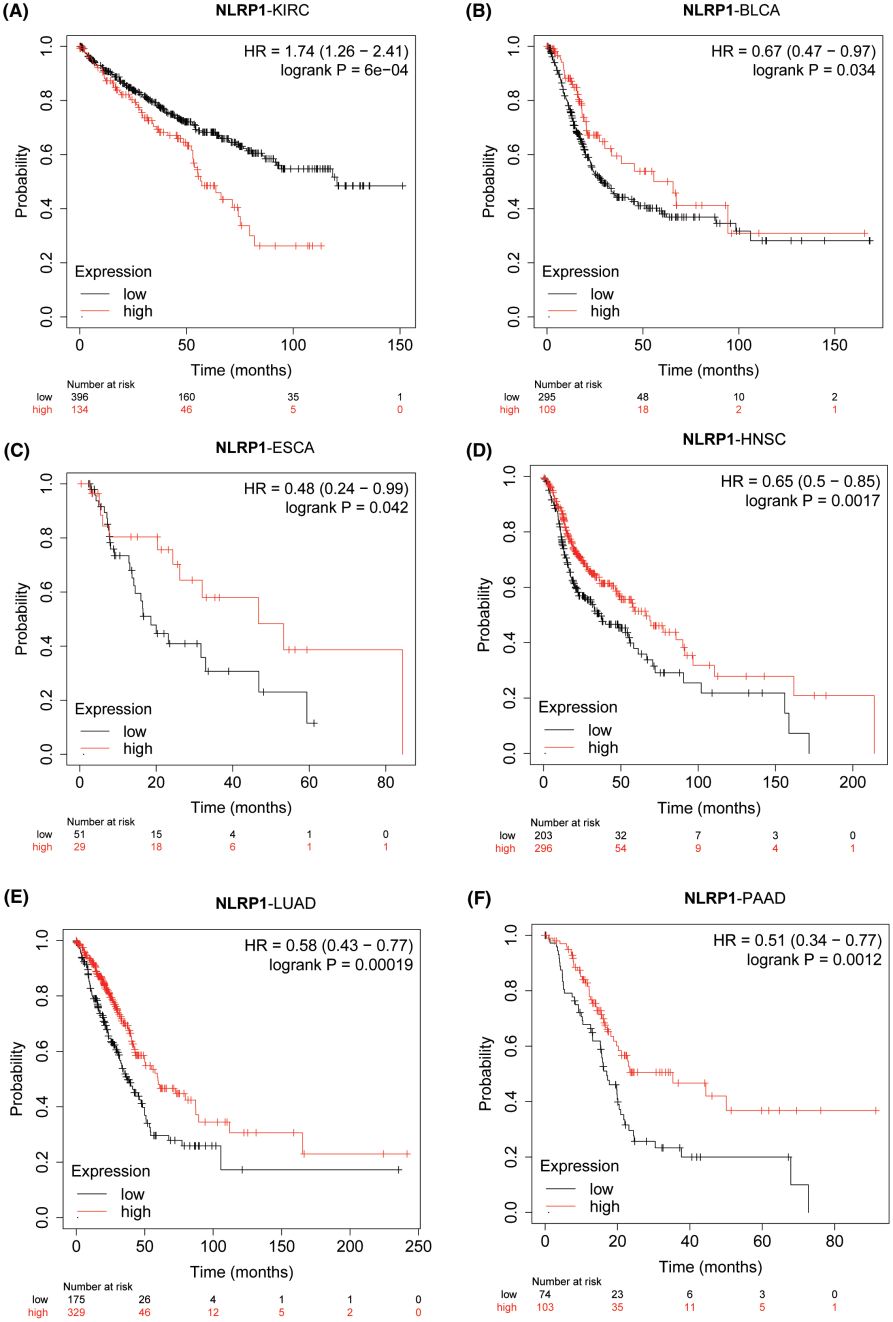
Kaplan–Meier analysis of overall survival (OS) between the low and high groups of NLRP1 in six cancer types including (A) KIRC, (B) BLCA, (C) ESCA, (D) HNSC, (E) LUAD and (F) PAAD.

Kaplan–Meier plotter analysis was conducted to investigate the effect of differential NLRP1 expression on tumour prognosis. The analysis reveals that high expression of NLRP1 is correlated with poor OS for KIRC (HR = 1.74, log‐rank *p* = 6e‐04) (Figure 4A), in contrast, high expression of NLRP1 is positively related to OS in BLCA (HR = 0.67, log‐rank *p* = 0.034), ESCA (HR = 0.48, log‐rank *p* = 0.042), HNSC (HR = 0.65, log‐rank *p* = 0.0017), LUAD (HR = 0.58, log‐rank *p* = 0.00019) and PAAD (HR = 0.51, Log‐rank *p* = 0.0012) (Figure 4B–F). In the other tumours, there is no significant correlation of NLRP1 with OS. Collectively, NLRP1 emerges as a potential biomarker, as it exhibits significant correlations with the prognosis of various types of tumours.

**FIGURE 5:**
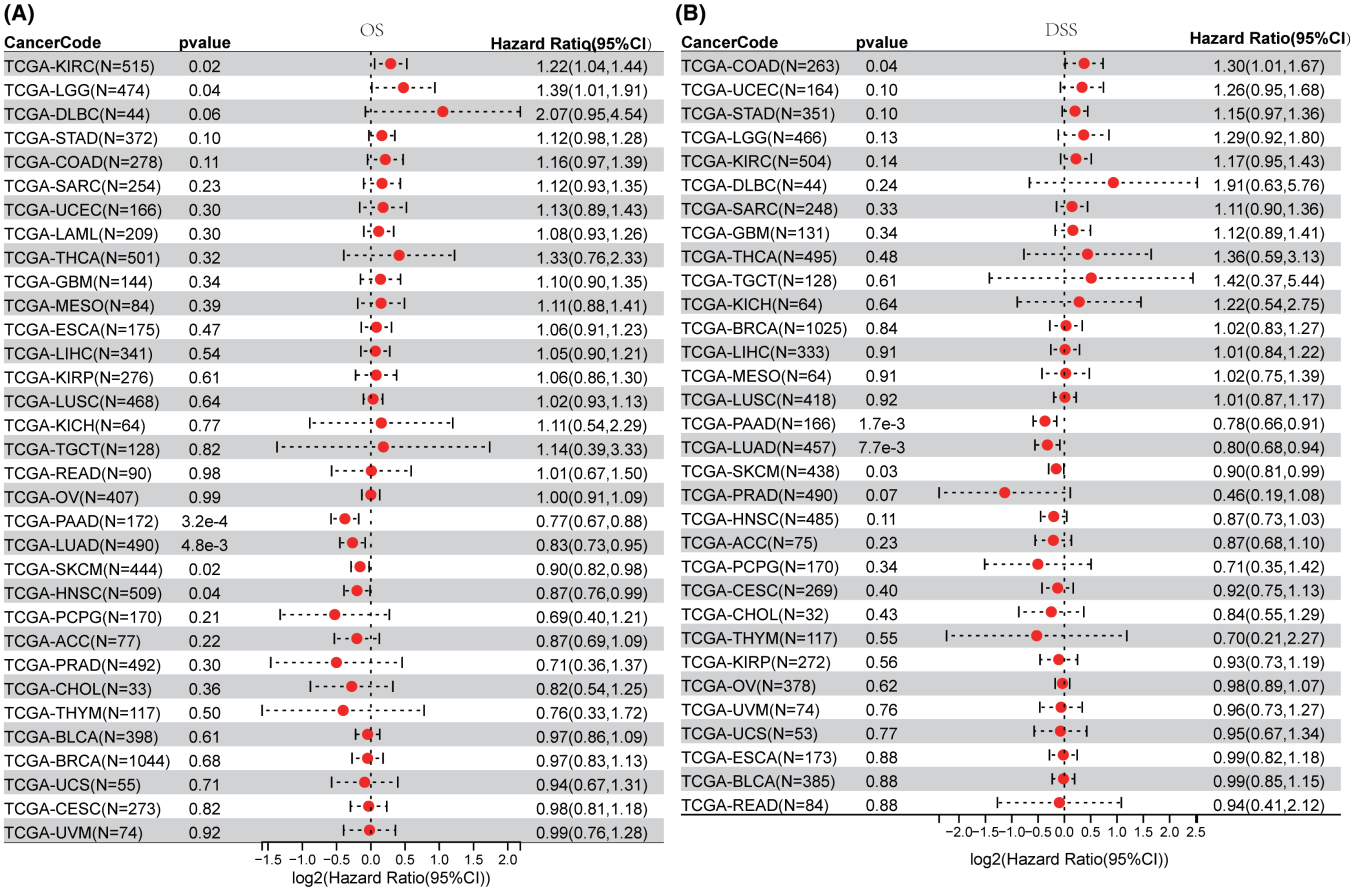
The forest plot for the association of NLRP1 expression with (A) overall survival (OS), and (B) disease‐specific survival (DSS) in each tumour type from TCGA. No DSS data are available for LAML.

To demonstrate the reliability of these results, a Cox proportional hazards regression model was developed to examine the NLRP1's expression in various cancers. The OS Forest plot (Figure 5A) shows that NLRP1 is a high‐risk gene in two types of tumours including KIRC (HR = 1.22, *p* = 0.02) and LGG (HR = 1.39, *p* = 0.06), while it is a low‐risk gene in four types of tumours, including PAAD (HR = 0.77, *p* = 3.2e‐4), LUAD (HR = 0.83, *p* = 4.8e‐3), SKCM (HR = 0.90, *p* = 0.02), HNSC (HR = 0.87, *p* = 0.04). In addition, the DSS forest plot (Figure 5B) shows high expression of NLRP1 is associated with an increased risk of death in COAD (HR = 1.30, *p* = 0.04). However, high expression of NLRP1 is associated with a decreased risk of death in PAAD (HR = 0.78, *p* = 1.7e−3), LUAD (HR = 0.80, *p* = 7.7e−3), and SKCM (HR = 0.90, *p* = 0.03). Altogether, the NLRP1 expression level is positively associated with prognosis in LUAD and PAAD.

### 3.4 Potential implications of NLRP1 differential expression across single‐cell types

**FIGURE 6:**
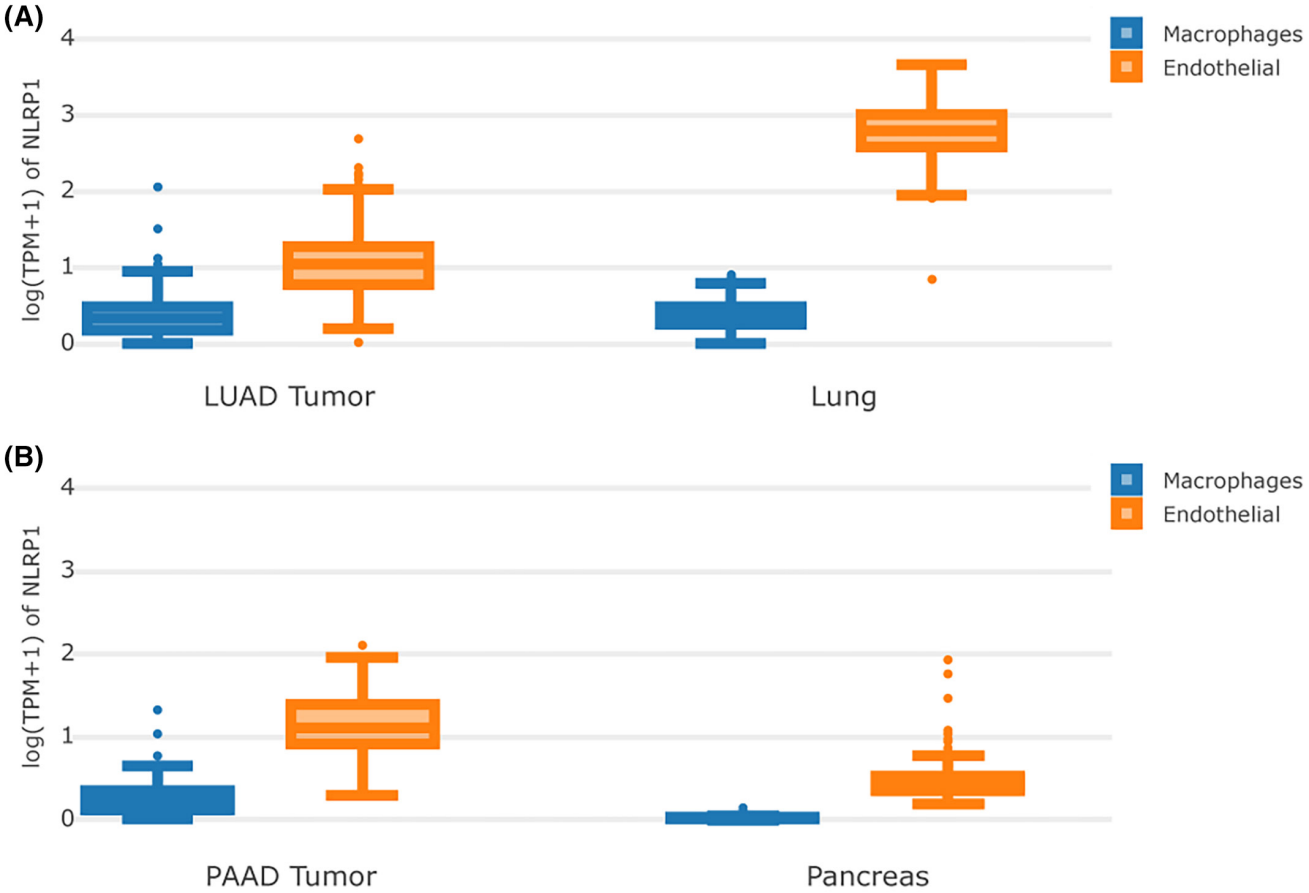
Expression of NLRP1 across macrophages and endothelial cells in LUAD and PAAD. (A) Differential expression of NLRP1 in macrophages and endothelial cells in lung adenocarcinoma and normal controls. (B) Differential expression of NLRP1 in macrophages and endothelial cells in pancreatic cancer and normal controls.

LUAD and PAAD patients with high levels of NLRP1 are associated with a favourable prognosis. However, NLRP1 expression is opposite between LUAD and PAAD. Specifically, NLRP1 is down‐expressed in LUAD, whereas over‐expressed in PAAD (Figure 3). The expression of NLRP1 in immune cells and other cells (taking macrophage and endothelia cells as examples, respectively) is examined to better understand this phenomenon (Figure 6). In normal lung tissue, NLRP1 is expressed in both macrophage and endothelial cells. However, its expressions in endothelial cells are obviously higher than in macrophages. In LUAD, the expressions of NLRP1 are decreased significantly in endothelial cells, while also reduce in macrophages though minutely (Figure 6A). This observation may explain that the downregulation of NLRP1 accompany with the unfavourable prognosis in LUAD. On the other hand, in normal pancreatic tissue, NLRP1 has low expression in both cells, especially with extremely low expression in macrophages. In PAAD, the expressions of NLRP1 are increased in endothelial cells, but increase with only moderation when compared to its decrease from normal lung tissue to LUAD. In addition, NLRP1 is also upregulated significantly in macrophages when compared to normal pancreatic tissue (Figure 6B). This finding may rationalize that the upregulation of NLRP1 in PAAD can also improve the prognosis of tumour patients.

### 3.5 Association between NLRP1 interacting network and immunity pathways

**FIGURE 7:**
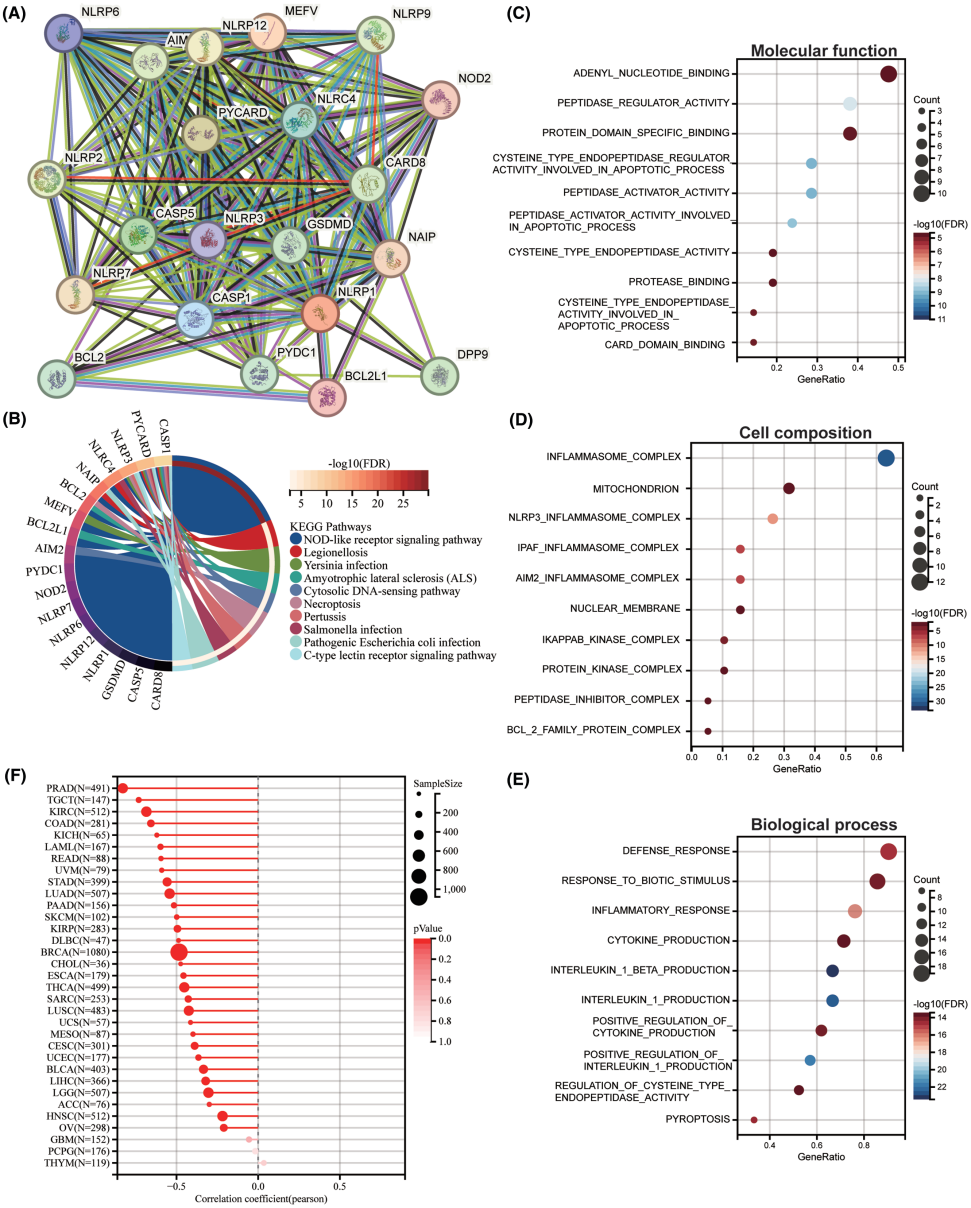
Interaction network, functional enrichment of NLRP1, and correlations between NLRP1 expression and tumour stemness. (A) Protein–protein interaction network by STRING software. (B–E): KEGG and GO enrichment analyses of NLRP1 and its PPI network proteins, showing the top 10 results. (B) KEGG pathway enrichment. (C) Molecular function in GO pathway enrichment. (D) Cell composition in GO pathway enrichment. (E) Biological process in GO pathway enrichment. (F) Correlations between NLRP1 expression and RNAss.

To investigate the biological importance and functional significance of NLRP1, PPI analysis was conducted. Figure 7A shows that 20 proteins, including AIM2, BCL2, BCL2L1, CARD8, CASP1, CASP5, DDP9, GSDMD, MEFV, NAIP, NLRC4, NLRP12, NLRP2, NLRP3, NLRP6, NLRP7, NLRP9, NOD2, PYCARD and PYDC1 form a protein network with NLRP1. Furthermore, as shown in Figure 7B, KEGG pathways analysis of the network proteins indicates that NLRP1 is related to the NOD‐like receptor signalling pathway, legionellosis, *Yersinia* infection, amyotrophic lateral sclerosis (ALS), cytosolic DNA‐sensing pathway, necroptosis, pertussis, *Salmonella* infection, pathogenic *Escherichia coli* infection, C‐type lectin receptor signalling pathway. Among these pathways, NOD‐like receptor signalling pathway is an immune‐related pathway^35^ and the other pathways were mostly related to infections, demonstrating the importance of NLRP1 in immunity. Furthermore, GO enrichment analysis of these proteins displayed that NLRP1 is significantly associated with molecular function such as adenyl nucleotide binding and peptidase regulator activity (Figure 7C), cell composition like inflammasome complex and mitochondrion (Figure 7D), biological processes like defense response and response to biotic stimulus (Figure 7E). These data indicate that NLRP1 is associated with immunity pathways and can act as a potential immunotherapeutic target.

### 3.6 Negative correlation between NLRP1 and tumour stemness

As shown in Figure 7F, NLRP1 is negatively associated with RNAss in 30 tumours with the statistical significance, including PRAD, TGCT, KIRC, COAD, KICH, LAML, READ, UVM, STAD, LUAD, PAAD, SKCM, KIRP, DLBC, BRCA, CHOL, ESCA, THCA, SARC, LUSC, UCS, MESO, CESC, UCEC, BLCA, LIHC, LGG, ACC, HNSC and OV, indicating NLRP1 is significantly negatively associated with tumour stemness.

### 3.7 Positive association between NLRP1 and immune microenvironment

**FIGURE 8:**
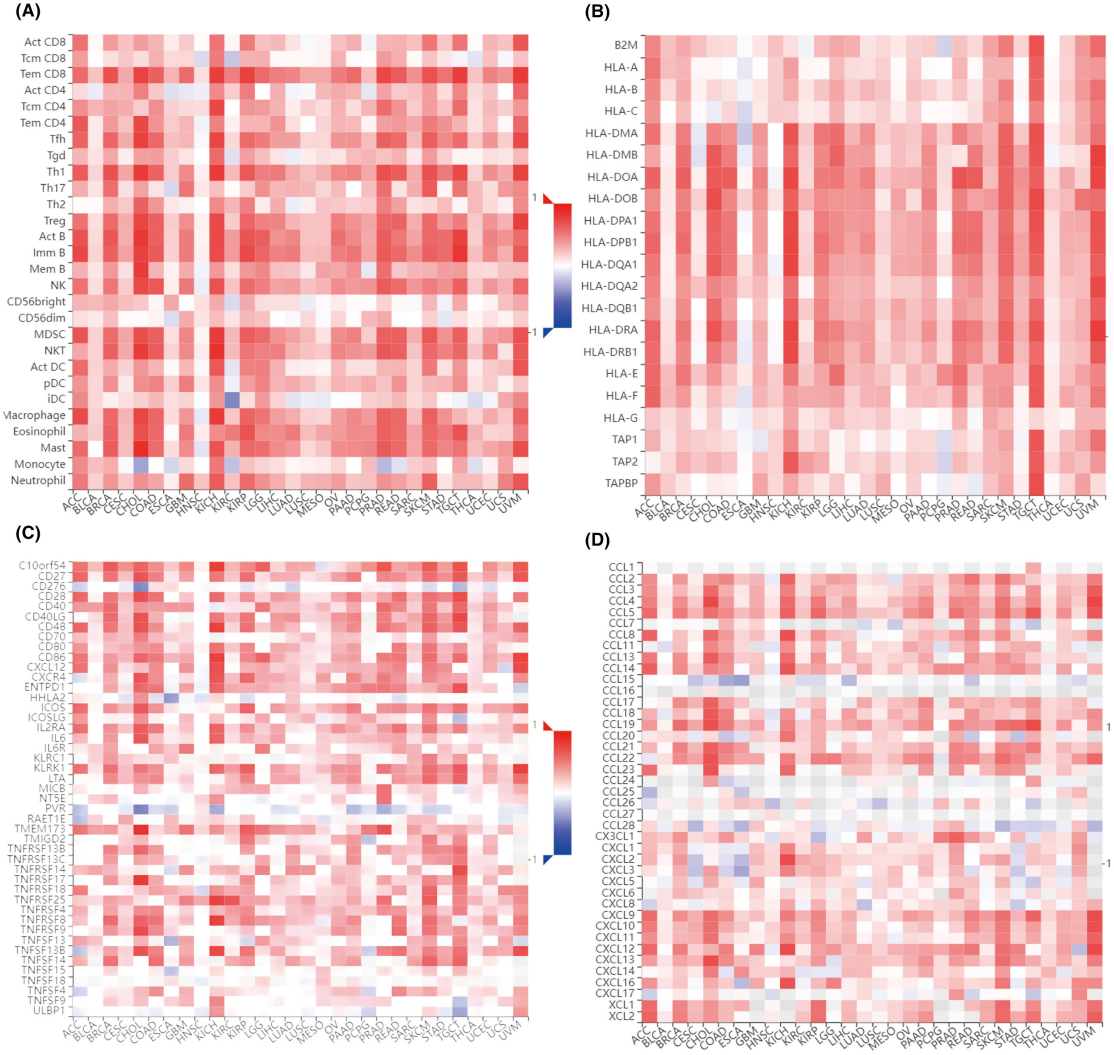
Correlations between the expression of NLRP1 and immunological signatures including (A) TILs; (B) MHC markers; (C) immune stimulating makers; (D) chemokine proteins in pan‐cancer.

Tumour immune infiltration is strongly related to clinical relevance, invasion, metastasis status and somatic mutation in cancer.^36^ Thus, we used the diverse immunological signatures to examine the relationship between tumour NLRP1 inflammasome and tumour immunity. Strikingly, the immune microenvironment analysis of NLRP1 shows that NLRP1 expression is positively correlated with 28 different types of TILs with statistical significance in most types of cancers, including LUAD and PAAD (Figure 8A).

Immunotherapy has been observed to have a substantial effect on the outcomes of certain cancers.^37^ As shown in Figure 8B–D, the expression level of NLRP1 is correlated significantly with three types of immune pathway‐related marker genes. In more detail, NLRP1 is positively related to most MHC genes with statistical significance in pan‐tumours except for CESC, ESCA, HNSC, PCPG and THCA (Figure 8B). NLRP1 is also significantly associated with most immune‐stimulating genes except for CD276, HHLA2, NTSE, PVR, TNFSF18 and UNBP1 in the HNSC‐excluded tumours **(**Figure 8C). Additionally, NLRP1 exhibits significant positive associations with the majority of chemokine proteins, except for CCL1, CCL15, CCL16, CCL24, CCL25, CCL26, CCL27, CCL28 and CXCL17 across pan‐tumours (Figure 8D). These results illustrate that the expression level of NLRP1 is notably correlated with the immune pathways.

**FIGURE 9:**
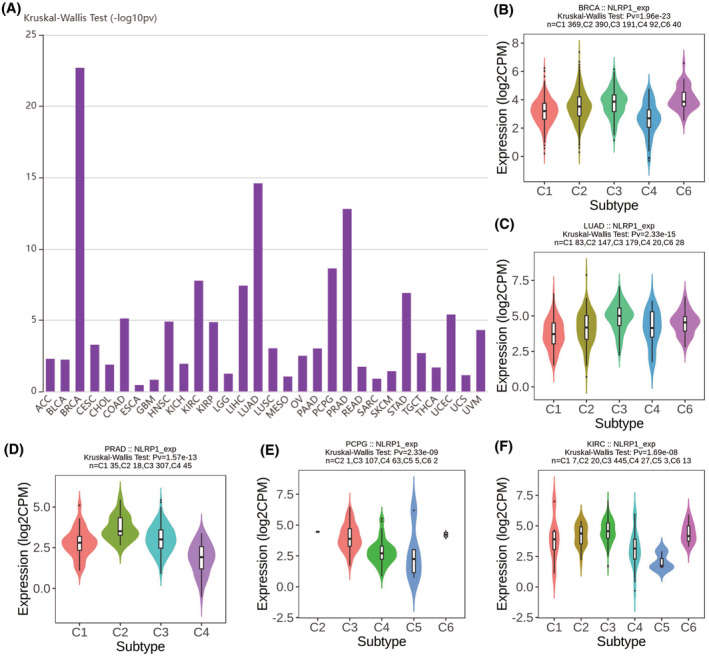
Expression variation of NLRP1 across different immune subtypes in (A) pan‐cancer; (B) BRCA; (C) LUAD; (D) PRAD; (E) PCPG; (F) KIRC.

Since NLRP1 is associated with immune infiltrating cells and immune pathway genes, further studies were conducted to determine whether it is related to common immune subtypes of tumours. Figure 9A shows that NLRP1 expression is significantly variated among different immune subtypes in most of cancers. Specifically, NLRP1 has top high correlations with immune subtypes in BRCA, LUAD, PARD, PCPG and KIRC.

NLRP1 expression is significantly different in most tumour immune subtypes. Figure 9B–F further show that NLRP1 is highest expressed in the C6 subtype of BRAC, the C3 subtype of LUAD, the C2 subtype of PRAD, the C3 subtype of PCPG and the C3 subtype of KIRC among subtypes with more than five samples. It has been reported that different immune subtypes have diverse influences on tumour prognosis.^38^, ^39^ The different impacts of NLRP1 on the prognosis of various tumour subtypes may be explained by the variable expression of NLRP1 in distinct subtypes of tumours.

### 3.8 Association between NLRP1 and immune/metastasis‐related pathways

**FIGURE 10:**
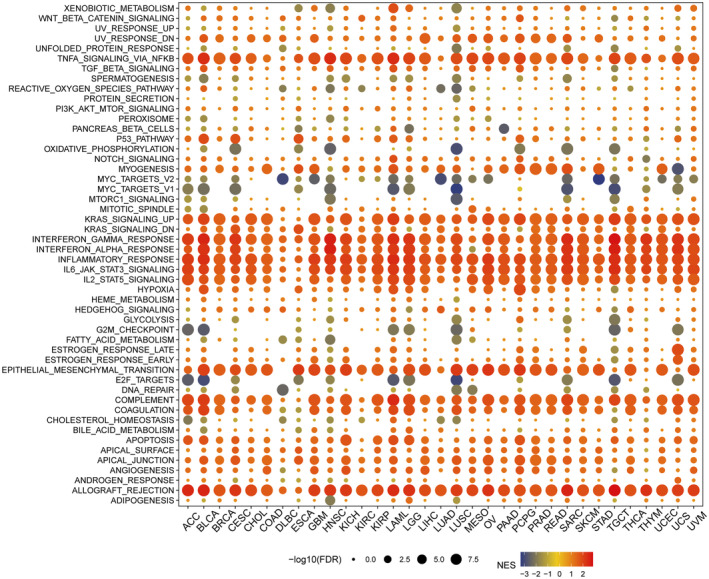
Association of NLRP1 expression with signalling pathways across 33 cancer types. Red represents enrichment in tumour samples with high NLRP1 expression and blue represents enrichment in tumour samples with low NLRP1 expression.

Insight of NLRP1 expression is significantly different in most tumour immune subtypes, thus, we further evaluated the relationship between NLRP1 levels and pathways using GSEA. Multiple signalling pathways are enriched significantly in tumour samples with high NLRP1 expression, including allograft rejection, angiogenesis, apical junction, apoptosis, coagulation, complement activation, epithelial‐mesenchymal transition (EMT), IL‐2/STAT5 signalling, IL‐6/JAK/STAT3 signalling, inflammatory response, interferon‐alpha response, interferon‐gamma response, KRAS signalling, TNF‐alpha signalling via NF‐kB and the p53 pathway etc. (Figure 10). Conversely, in tumour samples with low NLRP1 expression, there is a notable enrichment of signalling pathways, include E2F targets, G2/M checkpoint, MYC targets version 1, MYC targets version 2 and oxidative phosphorylation (Figure 10). Overall, the GSEA analysis suggested that high NLRP1 expression is positively associated with immune‐related pathways in tumours, while negatively linked to metastasis‐related pathways. These findings demonstrate that NLRP1 may develop into a novel immunological biomarker in tumours.

### 3.9 Potential association between NLRP1 and tumour metabolic reprogramming

**FIGURE 11:**
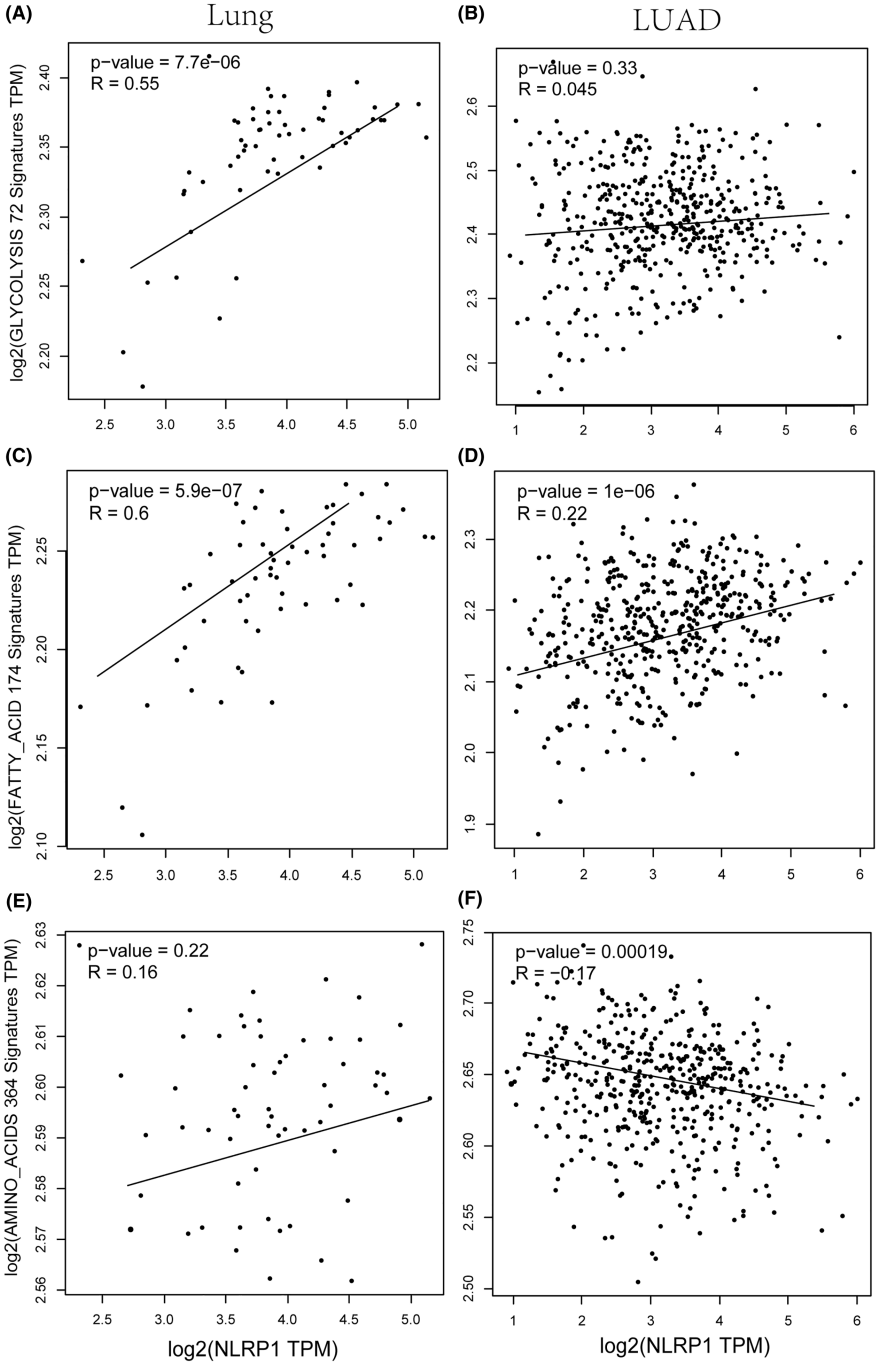
Correlation between the expression of NLRP1 and those of (A) glycolysis metabolism genes in normal samples; (B) glycolysis metabolism genes in LUAD patients; (C) fatty acid metabolism genes in normal samples; (D) fatty acid metabolism genes in LUAD patients; (E) amino acid metabolism genes in normal samples; (F) amino acid metabolism genes in LUAD patients.

The interconnection between tumour immune microenvironment and tumour metabolism offers potential improvements in tumour immunotherapy through targeting altered tumour metabolic system.^40^ For LUAD, NLRP1 expression in normal tissues is statistically significantly correlated with genes regulating glycolysis metabolism (Figure 11A,B). NLRP1 is also correlated with genes involved in fatty acid metabolism more positively when compared with LUAD samples (Figure 11C,D). Moreover, NLRP1 expression in normal tissues demonstrates a positive correlation with genes related to amino acid metabolism, although this association lacks statistical significance. However, in tumours, there is a significant negative correlation between NLRP1 expression and genes involved in amino acid metabolism (Figure 11E,F). These data may indicate that NLRP1 contributes to metabolic reprogramming. The down‐regulated NLRP1 decreases the tumour metabolic activity in LUAD, demonstrating the potential relationship between NLRP1 and tumour metabolism.

### 3.10 Connection between NLRP1 mutations and tumour prognosis

**FIGURE 12:**
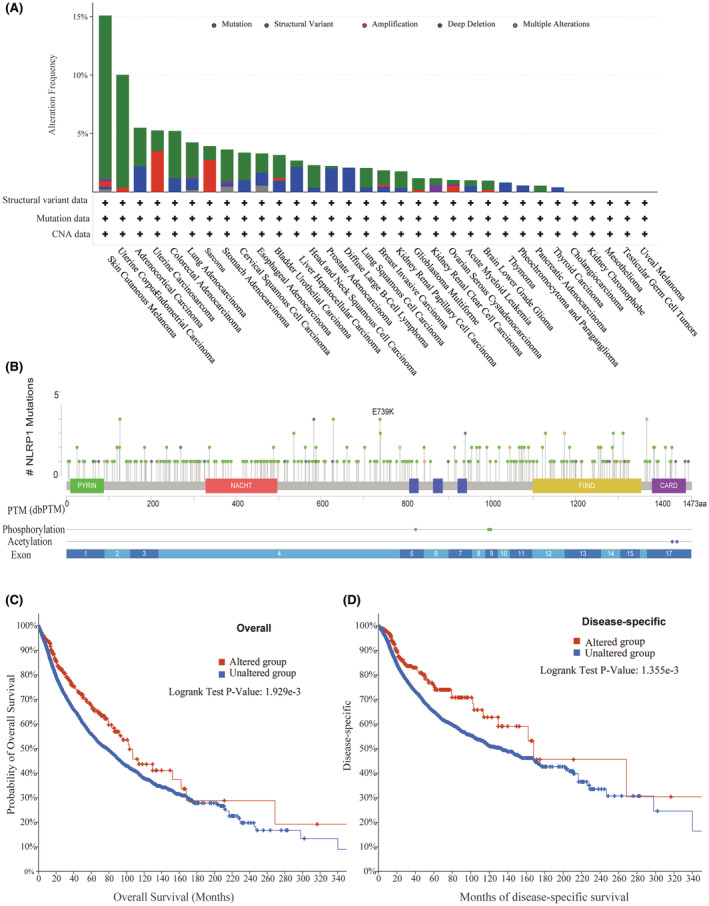
Relationship between NLRP1 mutations and the prognosis of tumour. (A) The alteration frequency of NLRP1 in pan‐cancer. (B) Exhibition of the type of alterations, mutated sites, and mutation cases. (C) Correlation between NLRP1 mutation and progression OS of pan‐cancer. (D) Correlation between NLRP1 mutation and progression DSS of pan‐cancer.

Some gene mutations are common in some malignancies and are strong predictors of tumour prognosis.^41^, ^42^ According to the cBioPortal database, NLRP1 mutations are relatively common in SKCM (15%) and UCEC (10%) (Figure 12A). Specifically, there are 311 mutation sites in the NLRP1 (243 missense mutations, 44 truncation mutations, 1 in‐frame mutation, 15 shear mutations and 8 fusion mutations), with E739K being the most common mutation (Figure 12B). In addition, the association analysis between the gene alterations of NLRP1 and the clinical survival prognosis of pan‐cancer cases in the TCGA illustrates that the cases with the NLRP1 mutation has a good prognosis in OS (*N* = 10,803, *p* = 1.929e‐3) and DSS (*N* = 10,258, *p* = 1.355e‐3) (Figure 12C,D). Taking together, NLRP1 mutations can act as a predictor of tumour prognosis.

## 4 DISCUSSION

NLRP1 is associated with innate immunity.^7^ It holds a crucial role in tumour immunity as an important gene of cell pyroptosis.^8^ In addition to immunity, NLRP1 may also be related to metabolism. It has been reported that inflammasomes are associated with immune and metabolic diseases.^5^ Thus, the relationship between NLRP1 and tumours has come to the foreground.

It is worthwhile to investigate the effect of NLRP1 on tumour immunology and metabolism since it may have a variety of essential functions in various types of tumours. Available studies previously reported that several scorch death‐related genes, including NLRP1 and NLRP3, play a key role in cancer immunity and may be employed as prognostic factors in pancreatic,^43^ breast,^44^ lung,^45^ and head and neck squamous cell carcinomas.^46^

In previous studies, the research on NLRP1 was restricted to specific tumours and did not consider tumour immunity or immune metabolism. Moreover, the relationship between NLRP1 mutations and expression level with patient prognosis, as well as NLRP1 and tumour immunity, was unclear. According to this study, NLRP1 plays a key role in expression, prognosis, immunity, metabolism, stemness and mutation in pan‐cancer (a summary of the results can be found in Table 1).

**TABLE 1:** A comparison of the findings in this study with the earlier related studies in the literature.

No.	Interesting results	Thoroughly mentioned previously	Partially mentioned previously	Explored in this study
1	The expression of NLRP1 had cell specificity, and its distribution had tissue specificity	×	×	√
2	NLRP1 was significantly upregulated in 10 tumours and significantly downregulated in 18 tumours	×	√^6^, ^47^, ^48^	√
3	NLRP1 expression was significantly related to prognosis in multiple cancers. A significant correlation existed between the elevated NLRP1 expression and the good prognosis of PAAD and LUAD	√^43^, ^44^, ^45^, ^46^	\	√
4	RNAss tumour stemness score and NLRP1 expression were significantly negatively correlated in all 33 tumours	×	×	√
5	The expression of NLRP1 was remarkably correlated with 28 infiltrating immune cells in most tumours	×	√^6^, ^48^	√
6	A robust and significant relationship existed between NLRP1 and expression levels of recognized immune stimulants, MHC molecules, and chemokine proteins in most cancers	×	×	√
7	NLRP1 expression levels were significantly correlated with immune subtypes in tumours	×	×	√
8	NLRP1 had relatively high mutation levels in SKCM (15%) and UCEC (10%). NLRP1 had 311 mutation sites in pan‐cancer	×	×	√
9	Patients with NLRP1 mutations had a better prognosis in terms of OS and DSS	×	×	√
10	The analyses of PPI, KEGG, GO and GSEA indicate that NLRP1 is associated with inflammatory response, inflammasome complex, immune‐related pathways and tumour metastasis‐related pathways	×	×	√
11	NLRP1 expression is metabolically relevant in tumours such as LUAD	×	×	√
12	The statistically significant increase in both endothelial and macrophage cells of the tumour samples compared with the normal control may account for the reason why the elevated expressions of NLRP1 in PAAD show a favourable effect on the prognosis of these two cancers	×	×	√

*Note*: The ‘Interesting results’ column summarizes the main research and innovation points of the study. The sign √ indicates that the innovation point was described in the study. The sign × indicates that the innovation point was not mentioned. The sign \ indicates that the point was partially mentioned in the literature.

Furthermore, the subsequent investigations into the functions of NLRP1 by our study yielded intriguing discoveries.

### 4.1 NLRP1 is tightly linked to the tumour immune microenvironment

Immunological checkpoint (IC) therapy offers a new idea for cancer treatment and increases the likelihood that more patients with metastatic disease may have long‐term clinical remission and even be cured, but IC therapy has little impact on some patient populations.^49^ Unfortunately, there are no measures to determine whether IC therapy is effective. Thus, it is necessary to find new biomarkers or therapeutic strategies for cancer treatment.

This study shows compelling evidence for the relationship between NLRP1 expression and immune infiltration. For example, lower expression of NLRP1 in LUAD (Figure 3) are substantially associated with low levels of immune cell infiltration (Figure 8A) and poor prognosis (Figures 4E and 5), which is in consistent exactly with Shen's study.^6^ Moreover, we report that NLRP1 expression is significantly different in various tumours for the first time (Figure 3A) and the variations are correlated with immune cell infiltration, immune subtypes and immune‐related pathways (Figures 8, 9, 10). These findings underscore the pivotal role of NLRP1 in shaping the tumour immune microenvironment and its potential significance in tumour immunotherapy. By examining the relationship between NLRP1 levels and the immune environment, this study aims to provide valuable insights that could inform the development of immunotherapy across a range of tumours, offering novel guidance for cancer treatment.

NLRP1 and NLRP3 are two inflammasome sensors in the innate immune system that activate inflammasome complexes by sensing different stimuli, which in turn promote a variety of immune responses, including proinflammatory cytokines.^7^ NLRP3 was activated after radiation and promoted IL‐1 signalling on dendritic cells, resulting in T‐cell activation and antitumor immunity.^50^ However, NLRP3 promotes the immune infiltration of tumours and is closely related to immune escape by regulating IC.^51^ Unlike NLRP3, NLRP1 is positively correlated with high degree of immune cells infiltration that enhances the immune microenvironment of tumours, thereby improving patient prognosis in tumours, such as in LUAD.^6^ In addition, thioredoxin system serves as an intrinsic checkpoint for innate immunity via suppressing NLRP1 activation.^52^ These data provide a hint of NLRP1 may regulate IC and serve as a new immunotherapeutic biomarker in tumours. According to Figures 7 and 10, NLRP1 mainly affects tumours through the inflammatory immune pathways, in which NLRP1 may play a common role with NLRP3.

### 4.2 NLRP1 regulates tumour progression by influencing the metabolic environment

The function of inflammasomes, particularly NLRP1 in tumour metabolism, has not yet been investigated. By influencing the metabolic environment, NLRP1 can regulate tumour progression. In the tumour microenvironment, the main metabolites of tumour cells, lactic acid and transforming growth factor‐β (TGF‐β) can inhibit the activation of inflammasomes to escape immune surveillance.^53^ The previous study revealed that increased metabolism in tumours contributes to growth and immune escape. In this study, NLRP1 is found to be more positively correlated with glycolysis metabolism and fatty acid metabolism in adjacent normal tissues than in LUAD (Figure 11A–D). Moreover, NLRP1 shows notably negative association with amino acid metabolism in LUAD (Figure 11F). Thus, NLRP1 may prefer to reprogram tumour energy metabolism, thereby suppress the progression of LUAD. However, the complex mechanism by how NLRP1 in LUAD regulates metabolic rewiring needs to be further investigated.

### 4.3 NLRP1 mutations are associated with good tumour prognosis

We reveal that the NLRP1 mutation may be related to a good prognosis for the tumours (Figure 12C,D). Since this study analyses the effect of NLRP1 mutations in pan‐cancers, the prognosis effect on a single tumour still needs further study. However, this study shows that NLRP1 mutations can be used as a reliable biomarker for the prognosis with good biological characteristics and better prognostic value in pan‐cancer.

### 4.4 NLRP1 expression negatively correlates with tumour stemness

Cancer stem cells (CSCs) are characterized as a kind of self‐renewing cell type that has been identified in most cancers, which contribute to the initiation and progression of most cancers.^54^ CSCs, as a minor subpopulation in cancers, are identified based on the expression of different cell surface markers and varied in different tumour types.^55^ Targeting the signals and tumour microenvironment of CSCs may be a novel approach for cancers treatment.^56^, ^57^ To date, the relationship between inflammasomes and tumour stemness has not been studied. In this study, NLRP1 is shown to be inversely connected with the tumour stemness index of RNAss, suggesting that high expression of NLRP1 can be accompanied by a decrease in tumour metastasis, as well as a comparative reduction of the drug resistance and self‐renewal ability of tumours.

### 4.5 NLRP1 expression in immune cells positively correlates with prognosis

Immune cell infiltration in the tumour microenvironment is strongly associated with tumour prognosis.^58^, ^59^ NLRP1 is differentially expressed in different cells. In this study, the expression of NLRP1 in immune cells is found to be comparable in both LUAD and normal lung tissue (Figure 6A). Nevertheless, the expression of NLRP1 in immune cells in PAAD differed dramatically from that in normal pancreatic tissue (Figure 6B). NLRP1 is highly expressed in nonimmune cells such as endothelial cells, and much less present in immune cells such as macrophages in normal pancreatic tissue (Figure 6B). NLRP1 increase significantly in endothelial cells in the PAAD, which may be the cause of NLRP1 being more highly expressed in PAAD than in normal pancreatic tissue. NLRP1 also increase notably in immune cells in the PAAD, which may be the cause of higher expressed NLRP1 showing good prognostics in the PAAD. In addition, NLRP1 high expressed PAAD has a high abundance of immune cell infiltration (Figure 10), corresponding to a good prognosis of NLRP1 in PAAD (Figure 5). Our results indicate that the higher the gene‐induced immune cell infiltration, the better the prognosis, which is in accord well with earlier studies in breast cancer^58^ and ovarian cancer.^59^ The immune cells in LUAD and PAAD tissues may contribute to anti‐tumour immune response. The study suggests that the different prognosis of NLRP1 in different tumours may be related to its variable expression in different cells.

## 5 CONCLUSION

This study sheds light on the multifaceted role of NLRP1 in cancer, revealing its impact on expression, prognosis, immunity, metabolism and stemness across various tumour types. NLRP1 emerges as a crucial factor in the shaping tumour immune microenvironment and regulating metabolism, with its mutations potentially offering valuable prognostic insights The findings underscore the importance of further exploration into the upstream and downstream regulatory mechanisms of NLRP1. Notably, NLRP1 exhibits distinct characteristics in different tumours, suggesting its abnormal expression influences on tumour metabolism and immune response within the tumour microenvironment. These insights not only enhance our understanding of NLRP1's involvement in cancer but also highlight its potential as both a therapeutic target and prognostic marker. However, the complexity of NLRP1's clinical significance across cancers underscores the need for comprehensive research to fully elucidate its mechanisms. Overall, the clinical significance of NLRP1 in cancer is important, and this study lays the foundation for future research and applications.

## AUTHOR CONTRIBUTIONS

**Yong Liao:** Formal analysis (equal); project administration (equal); software (equal); writing – original draft (equal). **Pinglian Yang:** Formal analysis (equal); writing – original draft (equal). **Cui Yang:** Formal analysis (equal); writing – original draft (equal). **Kai Zhuang:** Formal analysis (equal). **Aamir Fahira:** Writing – review and editing (equal). **Jiaojiao Wang:** Formal analysis (equal); funding acquisition (equal); project administration (equal); writing – original draft (equal); writing – review and editing (equal). **Zhiping Liu:** Funding acquisition (equal); supervision (equal); writing – review and editing (equal). **Lin Yan:** Writing – review and editing (equal). **Zunnan Huang:** Conceptualization (equal); funding acquisition (equal); project administration (equal); supervision (equal); writing – review and editing (equal).

## FUNDING INFORMATION

Thanks to the Key Discipline Construction Project of Guangdong Medical University (4SG23004G), and the Science and Technology Plan Project of Maoming (no. 2021570).

## CONFLICT OF INTEREST STATEMENT

The authors declare no conflict of interest.

## CONSENT

Not applicable.

## Supporting information


Data S1.

## Data Availability

The supplementary file provides a detailed and comprehensive overview of both the data and methodology, which thoroughly support the findings of this study.
