# Supplementary material for: Clinical signature and associated immune metabolism of NLRP1 in pan‐cancer
Source: J Cell Mol Med. 2024 Sep 24;28(18):e70100. doi: 10.1111/jcmm.70100 (PMC11422451; doi:10.1111/jcmm.70100)
Supplement: Supplementary file 1 — Data S1. [file JCMM-28-e70100-s001.docx]

**Supplementary File**

Clinical Signature and Associated Immune Metabolism of NLRP1 in Pan-Cancer

Yong Liao^#^, Pinglian Yang^#^, Cui Yang, Kai Zhuang, Aamir Fahira, Jiaojiao Wang, Zhiping Liu, Lin Yan, Zunnan Huang^*^

**1. Figure S1…………………………………………………………………………....2**

**2. Table S1 ………………………………………………………………………….…3**

**3. Guidelines and Steps for Online Analysis Using the Webserver…..……..……..4**

**3.1 NLRP1 Different Expression Pattern in Tissues and Cells…..…….……..……4**

**3.2 NLRP1 Differential Expression in Pan-cancer……….………….………...……6**

**3.3 Correlation Between NLRP1 and Tumor Prognosis…………..……….…….…9**

**3.4 Potential Implications of NLRP1 Differential Expression Across Single-cell Types…………………………….………………………………………..………….20**

**3.5 Association Between NLRP1 Interacting Network and Immunity Pathways.23**

**3.6 Negative Correlation Between NLRP1 and Tumor Stemness….………….….30**

**3.7 Positive Association Between NLRP1 and Immune Microenvironment.…….32**

**3.8 Association Between NLRP1 and Immune/Metastasis-related Pathways…....44**

**3.9 Potential Association Between NLRP1 and Tumor Metabolic Reprogramming ……………………………..……………………………………...50**

**3.10 Connection Between NLRP1 Mutations and Tumor Prognosis……….……57**

**1. Figure S1**

**
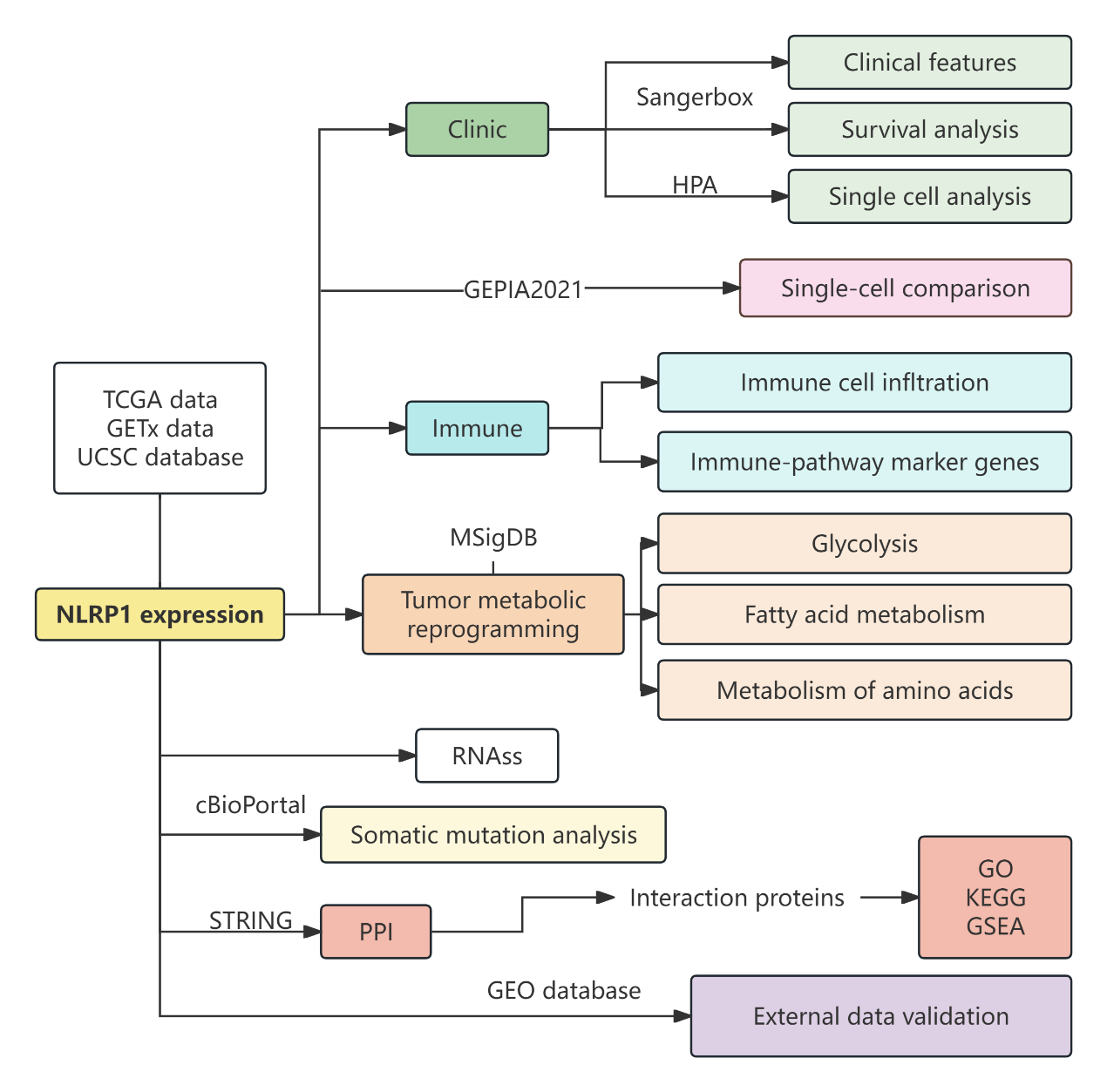
**

**Figure S1. A detailed study design tree diagram**

| **2. Table S1**  **Table S1. The 33 cancer types were analyzed.** | | | | |
| --- | --- | --- | --- | --- |
| **Cancer Type** | **Full name** | **Sample size** | | |
|  |  | **Normal** | **Tumor** | **Total** |
| ACC | Adrenocortical carcinoma | 128 | 77 | 205 |
| BLCA | Bladder urothelial cancer | 28 | 407 | 435 |
| BRCA | Breast invasive cancer | 292 | 1092 | 1384 |
| CESC | Cervix squamous cell carcinoma and endocervical adenocarcinoma | 13 | 304 | 317 |
| CHOL | Cholangiocarcinoma | 9 | 36 | 45 |
| COAD | Colon adenocarcinoma | 349 | 288 | 637 |
| DLBC | Lymphoid neoplasm diffuse large B-cell lymphoma | 0 | 48 | 48 |
| ESCA | Esophageal carcinoma | 668 | 181 | 849 |
| GBM | Glioblastoma multiforme | 1157 | 153 | 1310 |
| HNSC | Head and neck squamous cell carcinoma | 44 | 518 | 562 |
| KICH | Kidney Chromophobe | 168 | 66 | 234 |
| KIRC | Kidney renal clear cell carcinoma | 168 | 530 | 698 |
| KIRP | Kidney renal papillary cell carcinoma | 168 | 288 | 456 |
| LAML | Acute myeloid leukemia | 337 | 173 | 510 |
| LGG | Low grade glioma | 1157 | 509 | 1666 |
| LIHC | Liver hepatocellular carcinoma | 160 | 369 | 529 |
| LUAD | Lung adenocarcinoma | 397 | 513 | 910 |
| LUSC | Lung squamous cell carcinoma | 397 | 498 | 895 |
| MESO | Mesothelioma | 0 | 87 | 87 |
| OV | Ovarian serous cystadenocarcinoma | 88 | 419 | 507 |
| PAAD | Pancreatic adenocarcinoma | 171 | 178 | 349 |
| PCPG | Pheochromocytoma and paraganglioma | 3 | 177 | 180 |
| PRAD | Prostate adenocarcinoma | 152 | 495 | 647 |
| READ | Rectal adenocarcinoma | 10 | 92 | 102 |
| SARC | Sarcoma | 0 | 265 | 265 |
| SKCM | Skin Cutaneous Melanoma | 558 | 102 | 660 |
| STAD | Stomach adenocarcinoma | 211 | 414 | 625 |
| TGCT | Testicular germ cell tumor | 165 | 148 | 313 |
| THCA | Thyroid carcinoma | 338 | 504 | 842 |
| THYM | Thymoma | 0 | 122 | 122 |
| UCEC | Uterine corpus endometrial carcinoma | 23 | 180 | 203 |
| UCS | Uterine carcinosarcoma | 78 | 57 | 135 |
| UVM | Uveal melanoma | 0 | 80 | 80 |

**3. Guidelines and Steps for Online Analysis Using the Webserver (NLRP1 as a case study)**

**3.1 NLRP1 Different Expression Pattern in Tissues and Cells (Figure 2, analyzed on May 10, 2024)**

**1.** Access the Human Protein Atlas (HPA) Database:

Navigate to the Human Protein Atlas website: <https://www.proteinatlas.org>

**2.** Retrieve NLRP1 Data:

On the Human Protein Atlas homepage, use the search bar to find the NLRP1 protein by entering its gene identifier: ENSG00000091592. Alternatively, directly access the NLRP1 protein summary page using this URL: https://www.proteinatlas.org/ENSG00000091592-NLRP1.

**3.** View Protein Expression Levels in Tissues:

On the NLRP1 protein summary page, click on the “TISSUE” module. This will display the protein expression levels of NLRP1 across various normal tissues. Sort the tissues by expression levels by clicking on the “Expression” option within the “TISSUE” module. This action sorts the y-axis tissues according to NLRP1 expression levels (**Figure 2A** (main body manuscript)). Below is the screenshot representation of the above steps.


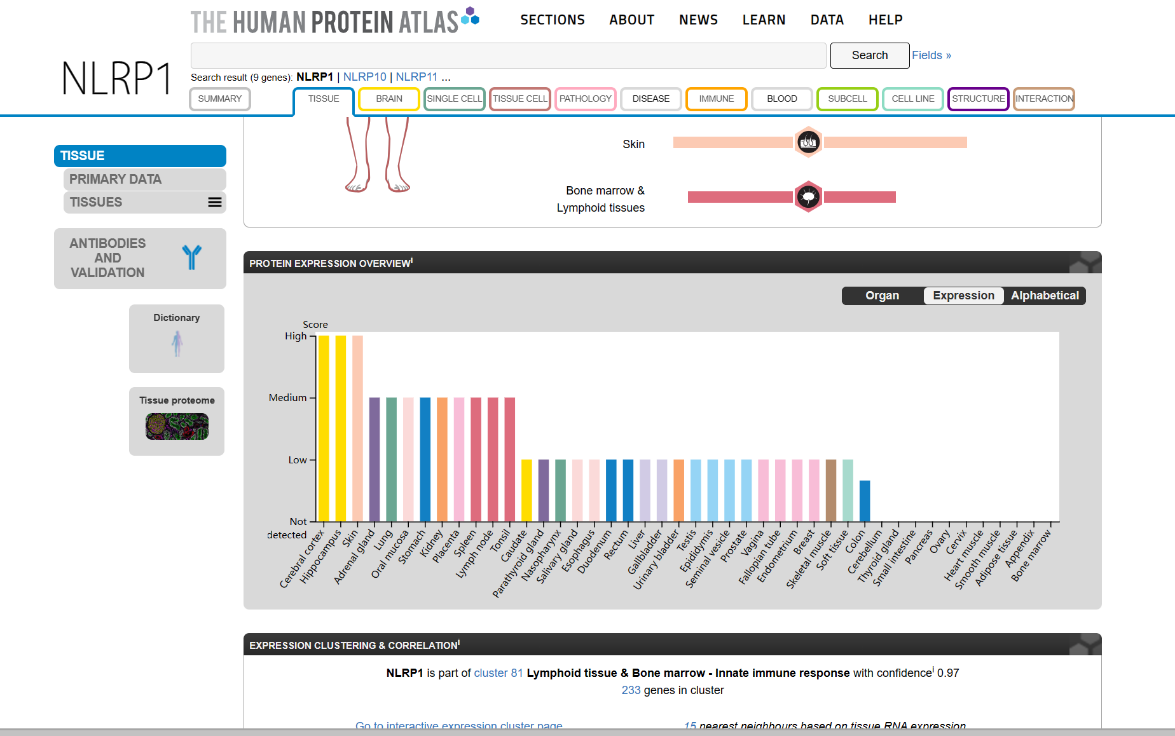


**4.** Investigate Single-Cell Expression Levels:

Next, to further investigate the expression level of NLRP1 at the single-cell level, click on the “SINGLE CELL” module on the NLRP1 protein summary page. This will display the expression data of NLRP1 across different single-cell types (**Figure 2B** (main body manuscript)).

**5.** Interpret the Data:

Review the single-cell expression data to understand the differential expression patterns of NLRP1 at the single-cell level. **Figure 2B** (main body manuscript) provides a visual representation of these expression levels, which can be used for further analysis and interpretation. Below is the screenshot representation of the above steps

**
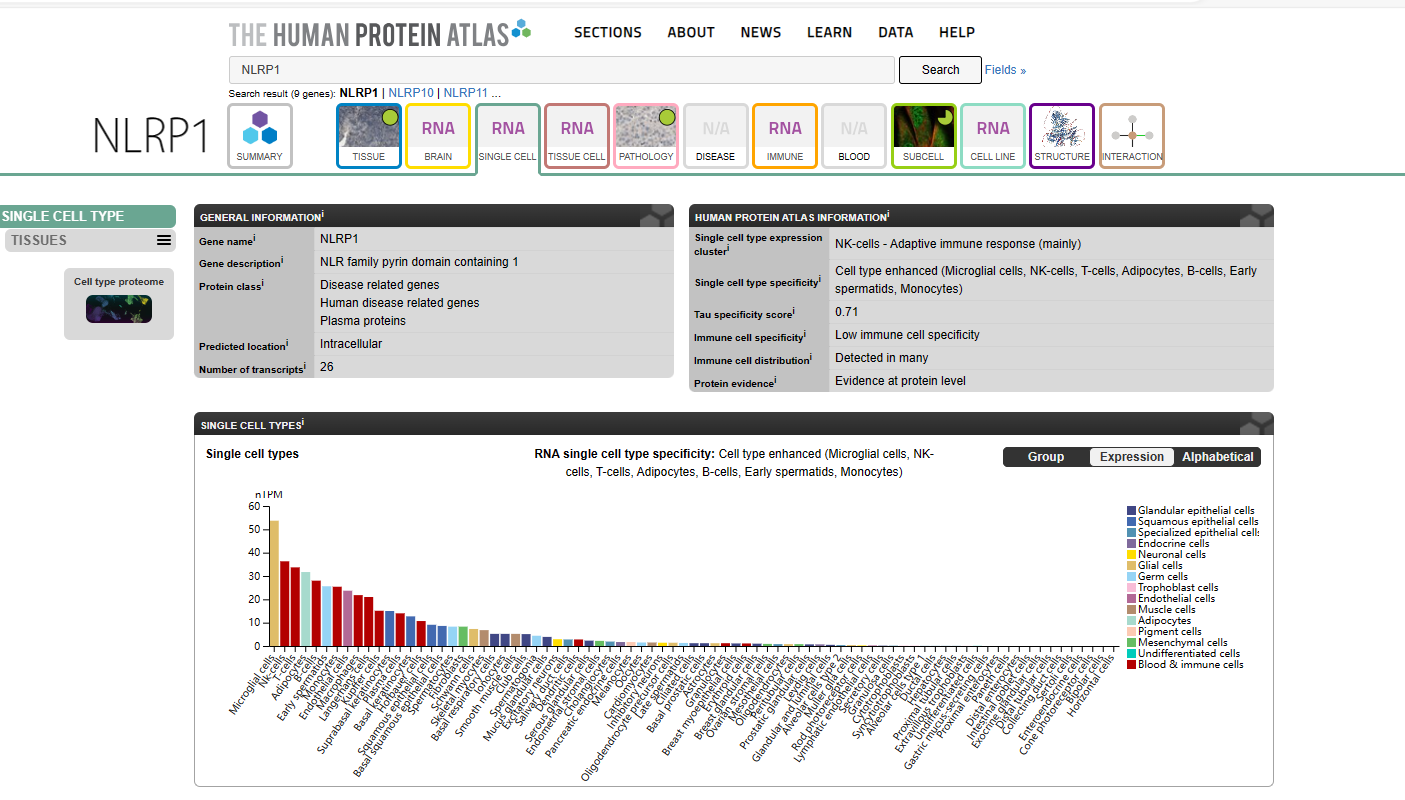
**

By following these steps, users can accurately retrieve and analyze the expression patterns of the gene of interest in both tissues and single cells using the Human Protein Atlas database.

**3.2 NLRP1 Differential Expression in Pan-cancer (Figure 3, analyzed on May 8, 2024)**

**1. Conducting Differential Expression Analysis**

**1.1** Access Sangerbox 3.0:

Navigate to the Sangerbox 3.0 website: http://vip.sangerbox.com/.

**1.2** Select the “Differential Gene Expression Analysis” Module:

Within the Sangerbox 3.0 interface, go to the “泛癌分析” (Pan-Cancer Analysis) section. Select the “基因表达差异分析” (Gene Expression Differential Analysis) module.

**1.3** Input Gene Information:

Enter the gene identifier ENSG00000091592 (NLRP1) in the search field.

**1.4** Choose Data Source and Sample Origin:

Set “TCGA+GTEx” as the data source to include both The Cancer Genome Atlas (TCGA) and Genotype-Tissue Expression (GTEx) project data. Select “所有样本” (All Samples) as the sample origin to include both cancerous and adjacent normal tissues.

**1.5** Set Data Transformation:

Choose “log2(x+0.001)” as the data transformation method to normalize the expression data.

**1.6** Generate and Interpret the Violin Plot:

Generate the plot by executing the analysis. A violin plot will be displayed, showing NLRP1 expression levels across multiple cancer tissues and adjacent normal tissues (**Figure 3A** (main body manuscript)). Review the violin plot to analyze the differential expression patterns of NLRP1 in various cancer types. Below is the screenshot representation of the above steps.

**
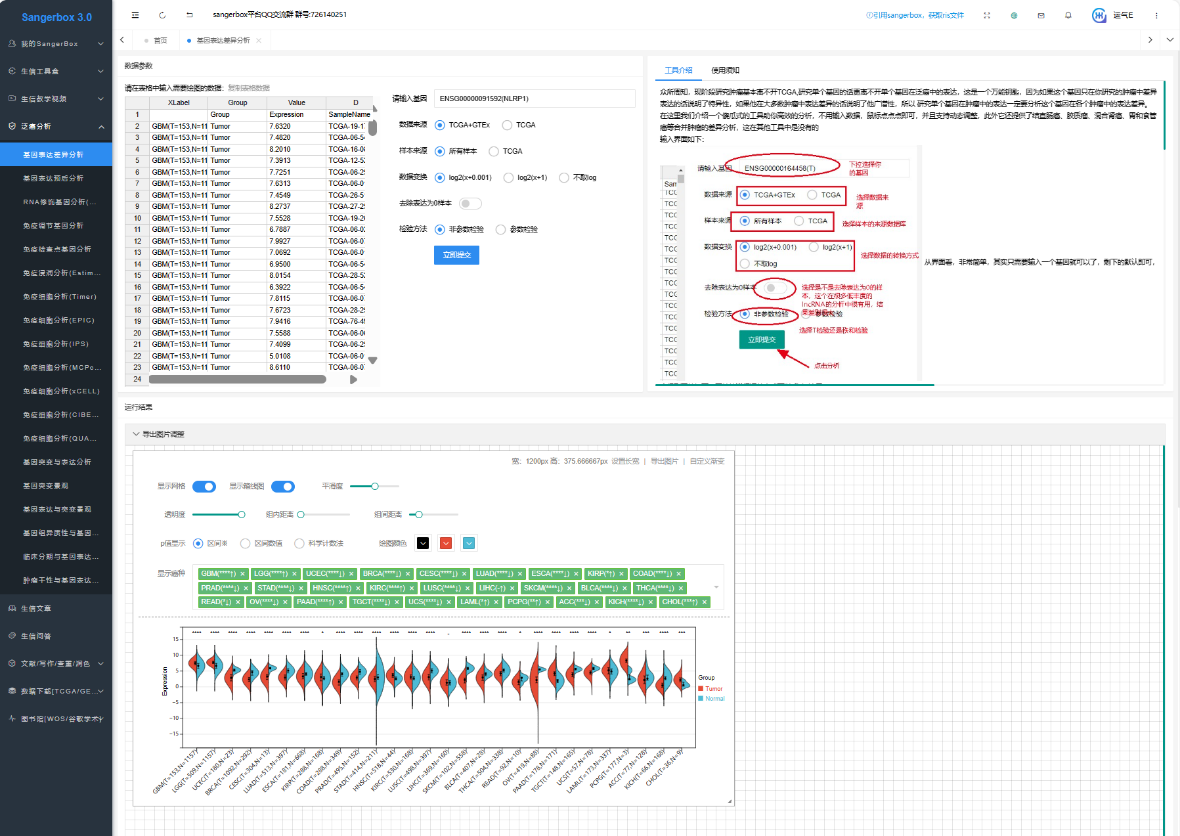
**

By following these steps, users can accurately retrieve and analyze the differential expression of the gene of interest across multiple cancer tissues and adjacent normal tissues using the Sangerbox 3.0 platform.

**2.** **Conducting External Validation**

To compare NLRP1 expression in tumor tissues and normal tissues in LUAD (Lung Adenocarcinoma), PAAD (Pancreatic Adenocarcinoma), and KIRC (Kidney Renal Clear Cell Carcinoma), use the R programming language to create box-and-line plots. The R code for this analysis is also detailed in the following GitHub repository: https://github.com/liaoyong0668/NLRP1-in-pancancer. Download and run the provided R script to generate the box-and-line plots comparing NLRP1 expression in LUAD, PAAD, and KIRC (**Figure 3B** (main body manuscript)).

**R code:**

library(reshape2)

library(ggpubr)

expFile="sum.txt"

data1=read.table(expFile, header=T, sep="\t", check.names=F,row.names = 1)

bioCol=c("#0066FF","#FF0000")

bioCol=bioCol[1:length(levels(factor(data1[,"cluster"])))]

p=ggboxplot(data1, x="type", y="NLRP1", fill="cluster",

ylab="Expression of NLRP1",

xlab="",

legend.title="Group",

palette=bioCol)

p+stat_compare_means(aes(group=cluster),

method="wilcox.test",symnum.args=list(cutpoints = c(0, 0.001, 0.01, 0.05, 1), symbols = c("***", "**", "*", "ns")),label = "p.signif")

**Result：**


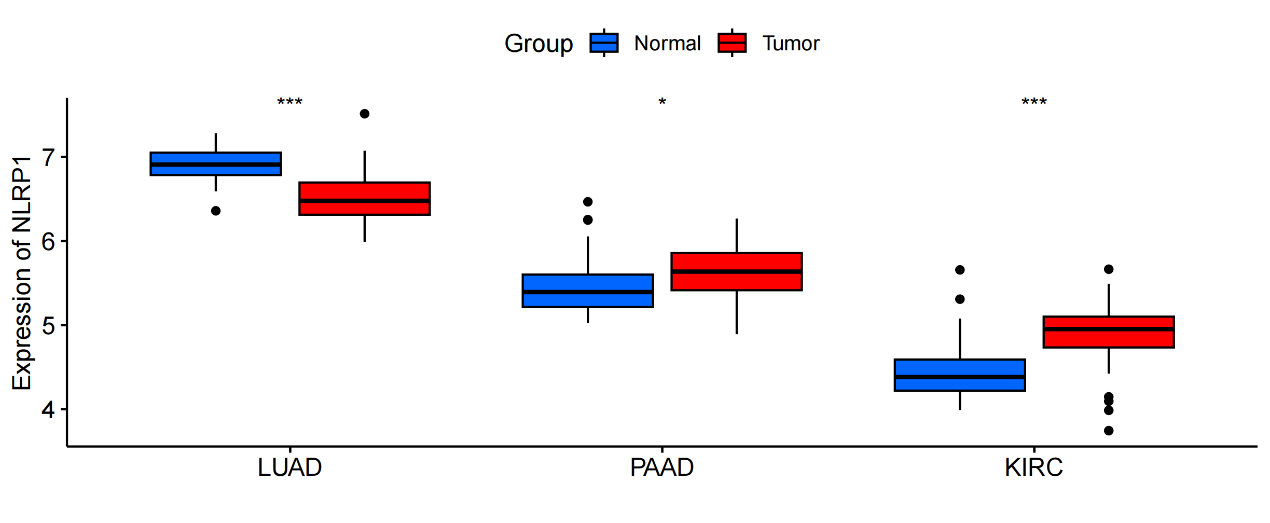


**3.3 Correlation Between NLRP1 and Tumor Prognosis (Figure 4 and Figure 5, analyzed on May 12, 2024)**

1. Access the Kaplan-Meier Plotter:

Navigate to the Kaplan-Meier Plotter website: http://kmplot.com/.

1. Select the “Pan-Cancer Prognostic Analysis” Module:

Click on the “Start KM Plotter for Pan-Cancer” module or directly access it via this URL: https://kmplot.com/analysis/index.php?p=service&cancer=pancancer_rnaseq.

1. Input Gene Information:

In the search box, enter the gene name “NLRP1”.

1. Select Sample Size and Data Source:

Choose the option “Select all (n=7489)” to include all 7,489 samples from 21 different types of cancer.

1. Run the Analysis:

Execute the analysis to generate Kaplan-Meier survival plots that display the prognostic significance of NLRP1 across the 21 types of cancer.

1. Review the Results:

**Figure 4** (main body manuscript) shows the overall survival analysis for NLRP1 across all cancers. Detailed screenshots of the analysis steps are provided below to illustrate the process using the Kaplan-Meier Plotter.

**
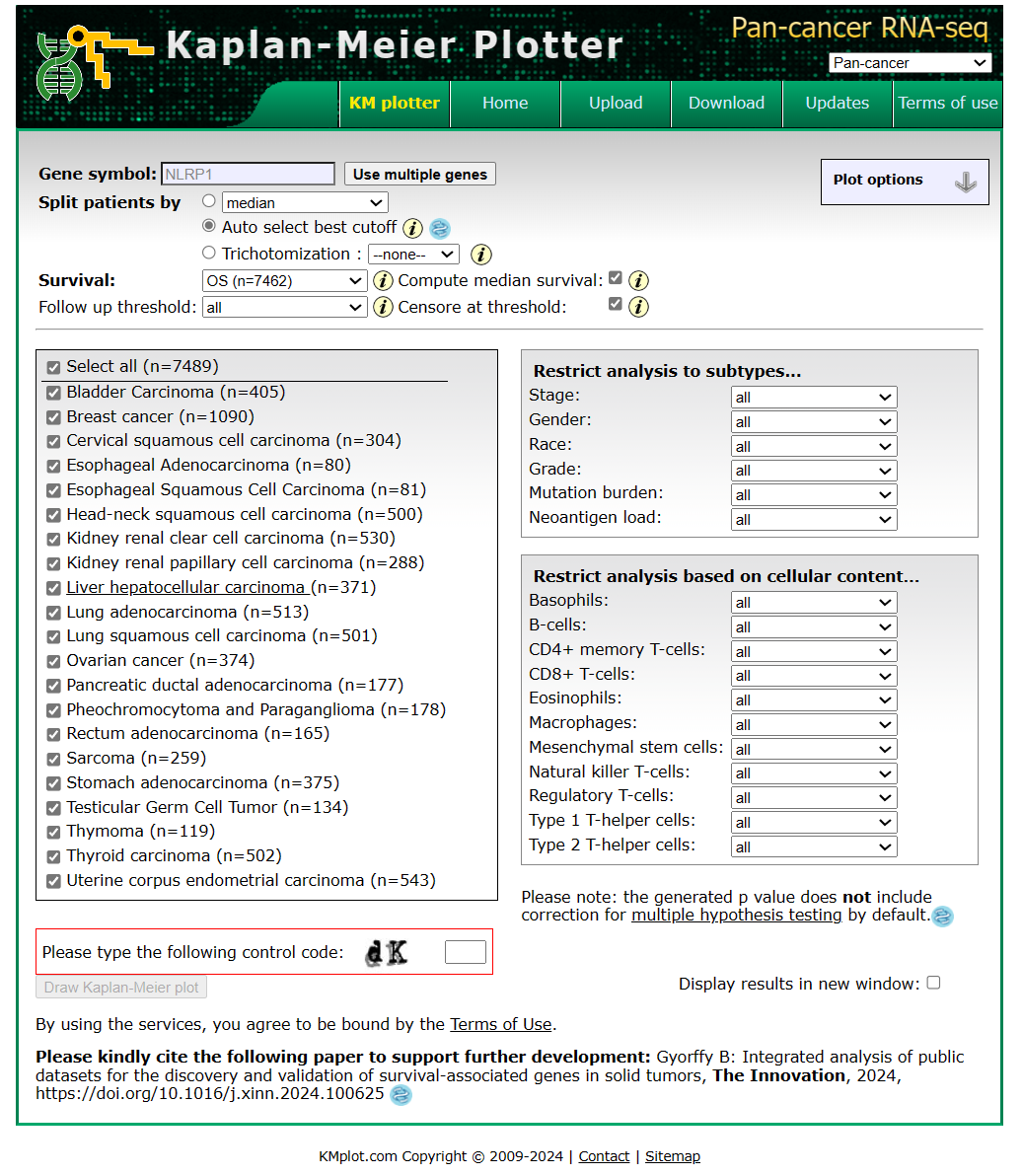
**

**
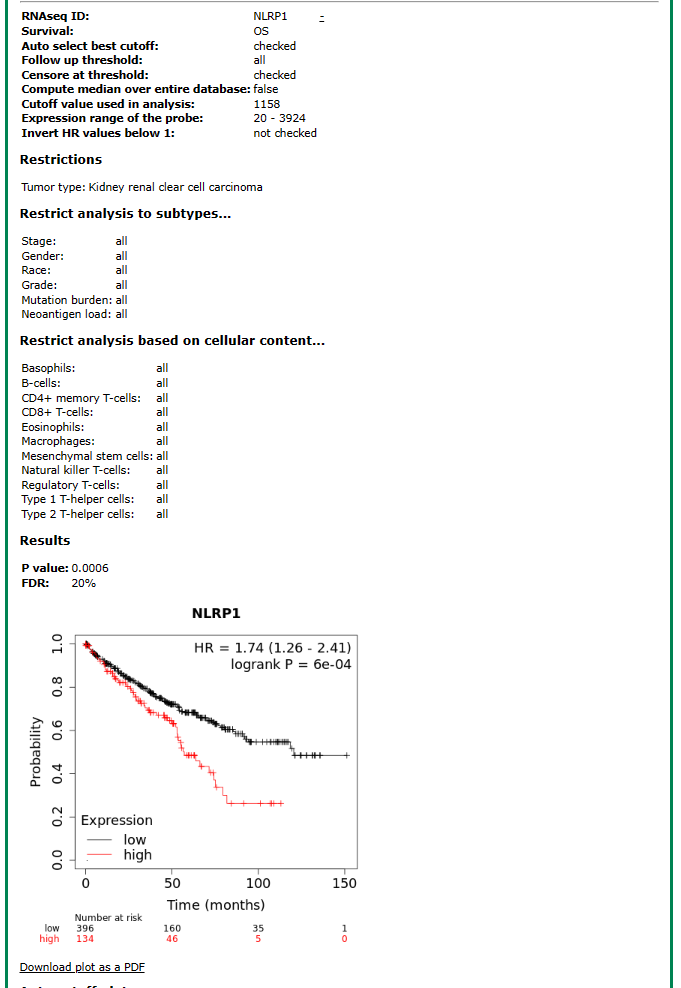
**

**
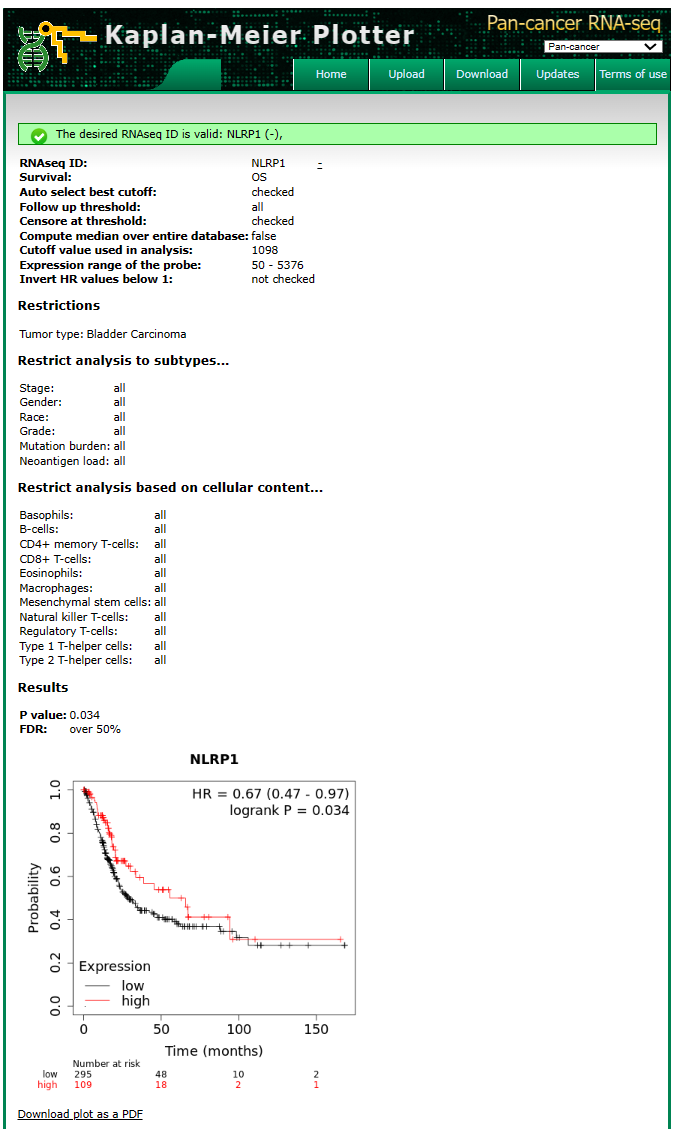
**

**
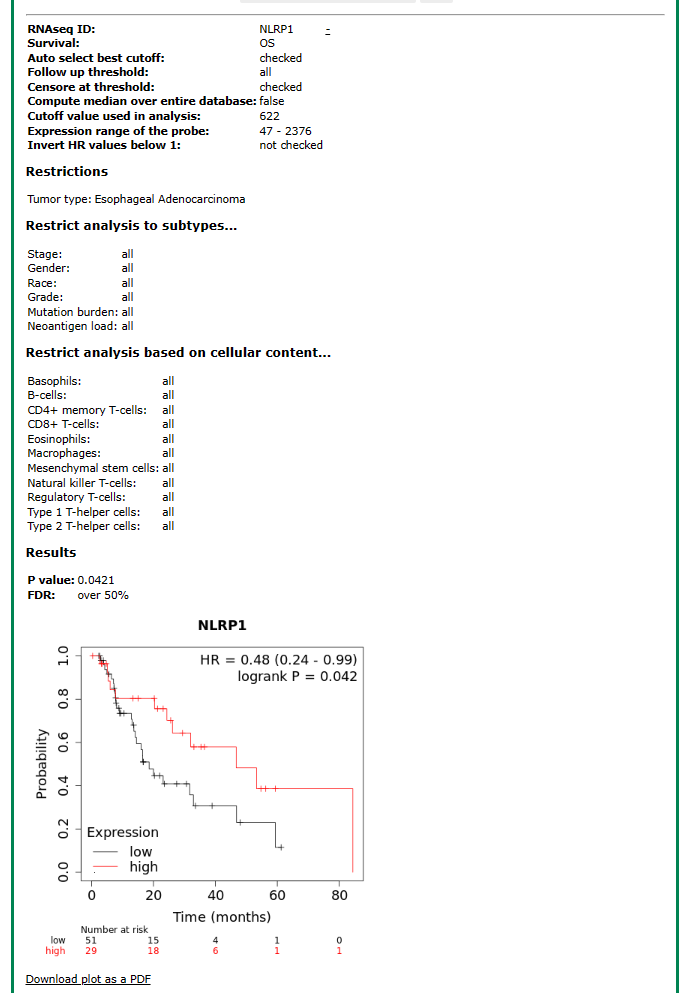
**

**
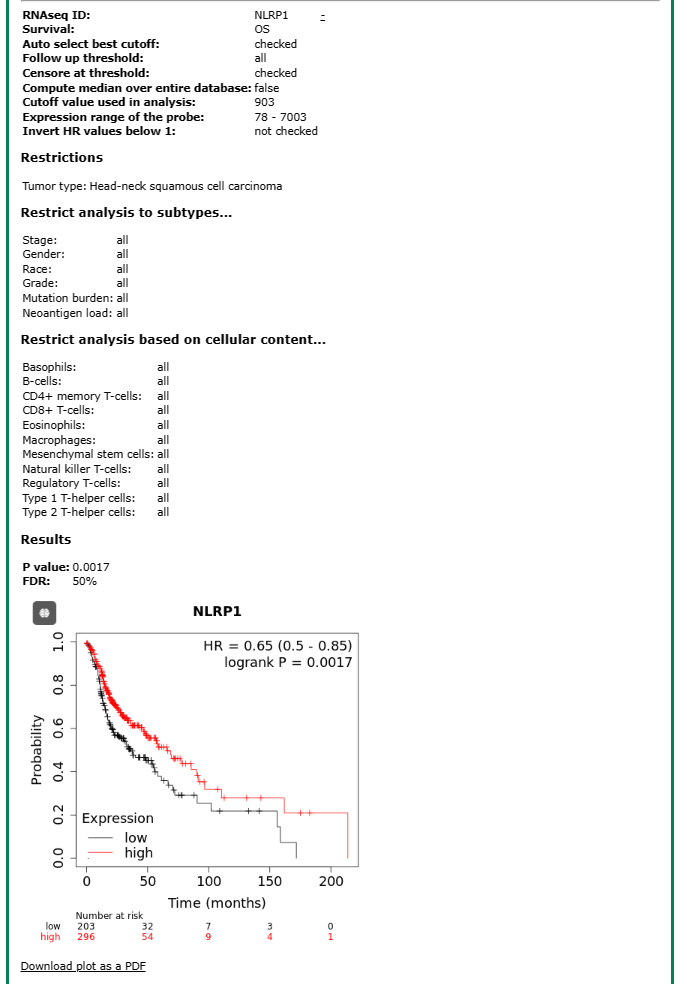
**

**
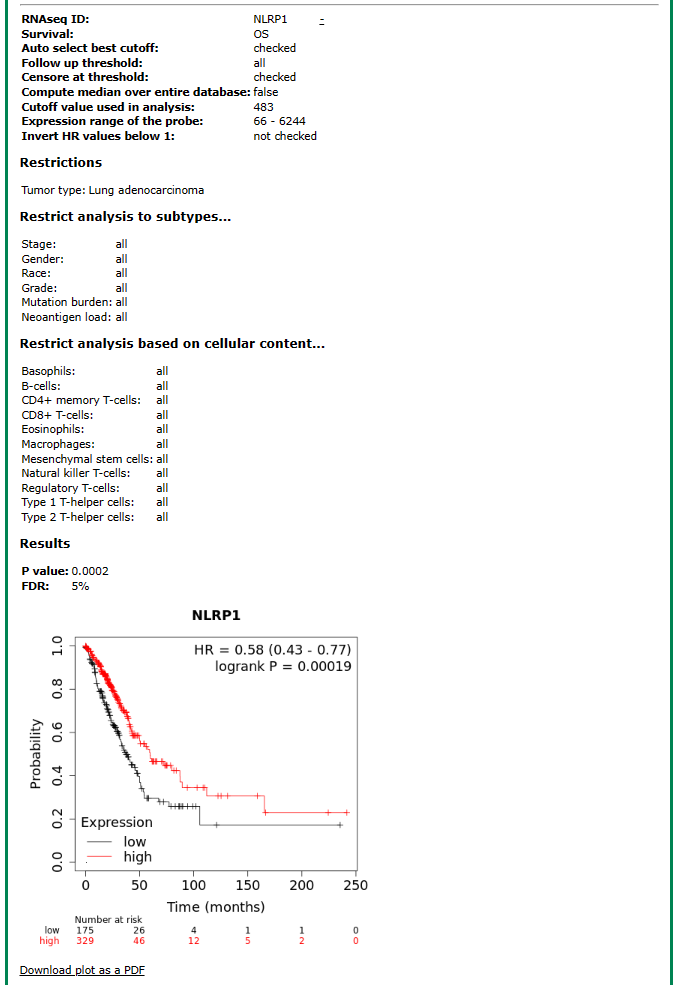
**

**
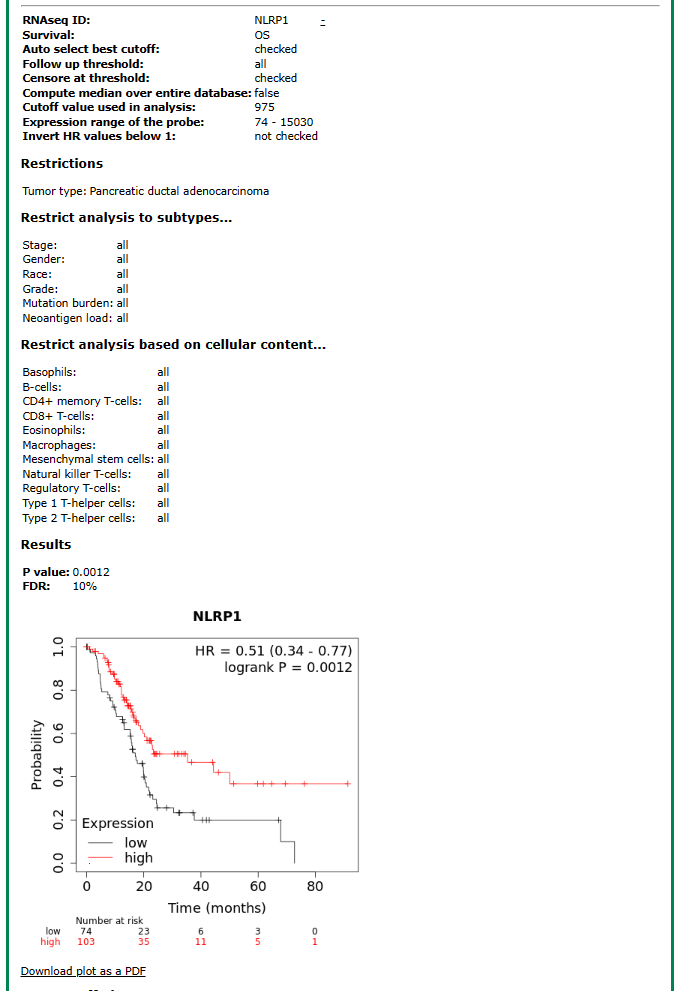
**

1. Perform Prognostic Analysis Using Sangerbox 3.0:

Access Sangerbox 3.0: http://vip.sangerbox.com/.

1. Select the “Prognostic Analysis” Module:

Within the Sangerbox 3.0 interface, go to the “泛癌分析” (Pan-Cancer Analysis) section. Select the “基因表达预后分析” (Gene Expression Prognostic Analysis) module.

1. Input Gene Information:

Enter the gene identifier ENSG00000091592 (NLRP1) in the search field.

1. Choose Data Source and Sample Origin:

Set “TCGA+GTEx” as the data source to include both The Cancer Genome Atlas (TCGA) and Genotype-Tissue Expression (GTEx) project data. Select “所有样本” (All Samples) as the sample origin.

1. Set Data Transformation:

Choose “log2(x+0.001)” as the data transformation method to normalize the expression data.

1. Configure Survival Analysis Parameters:

Set the minimum time to 30 days. Opt for survival data concerning both “Overall Survival” and “Disease-Specific Survival”.

1. Exclude Specific Cancer Types:

Exclude data from the following 11 types of cancers: TARGET-ALL, TARGET-ALL-R, TARGET-LAML, TARGET-NB, TARGET-WT, TCGA-COADREAD, TCGA-GBBLGG, TCGA-KIPAN, TCGA-SKCM-M, TCGA-SKCM-P, TCGA-STES.

1. Submit and Analyze:

Submit the module analysis to generate the prognostic results. Review the analysis results displayed in **Figure 5** (main body manuscript). Detailed screenshots of the analysis steps are provided below to illustrate the Prognostic Analysis Using Sangerbox. Detailed screenshots of the analysis steps are provided below to illustrate the Prognostic Analysis Using Sangerbox.

**
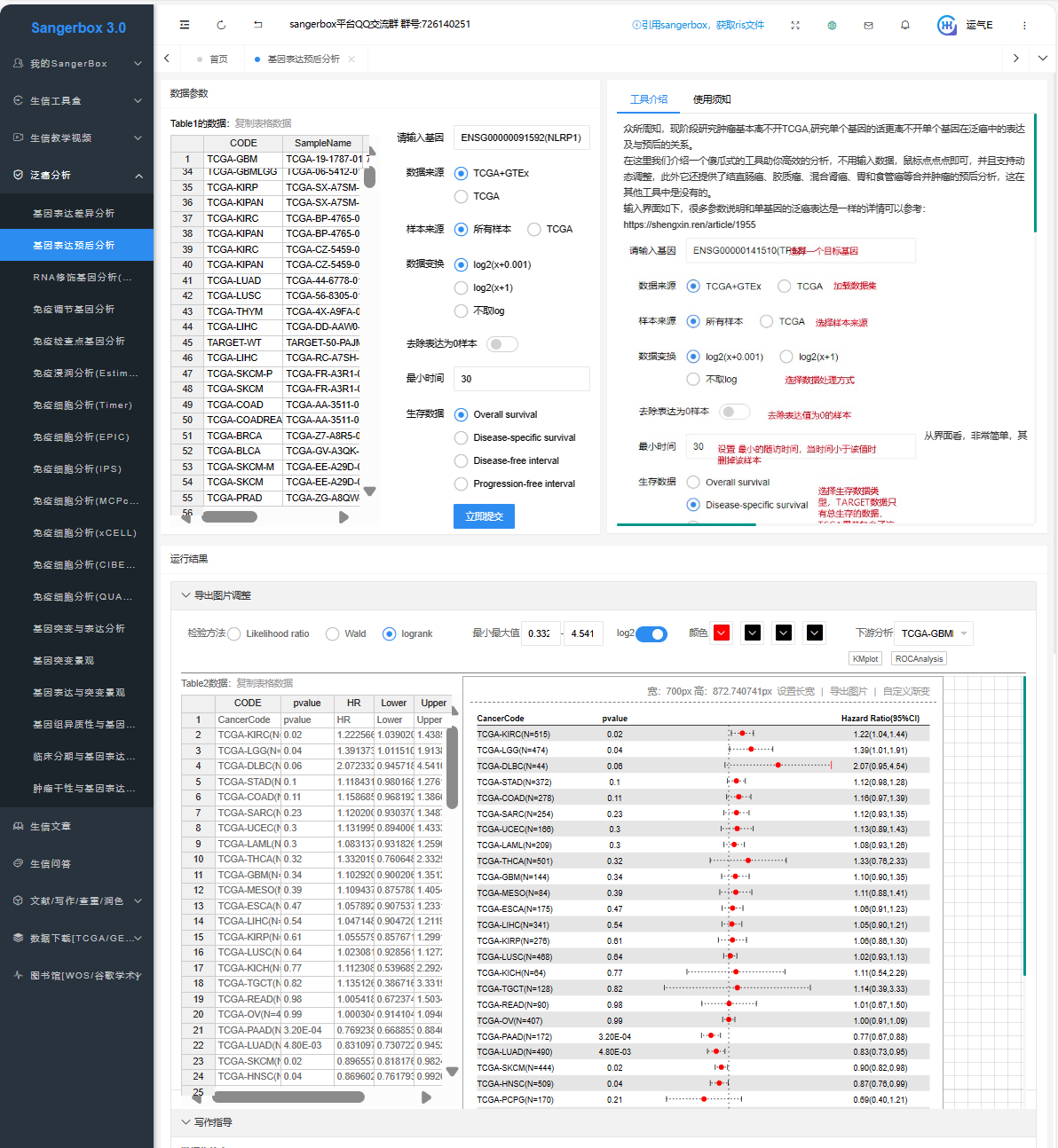
**

**
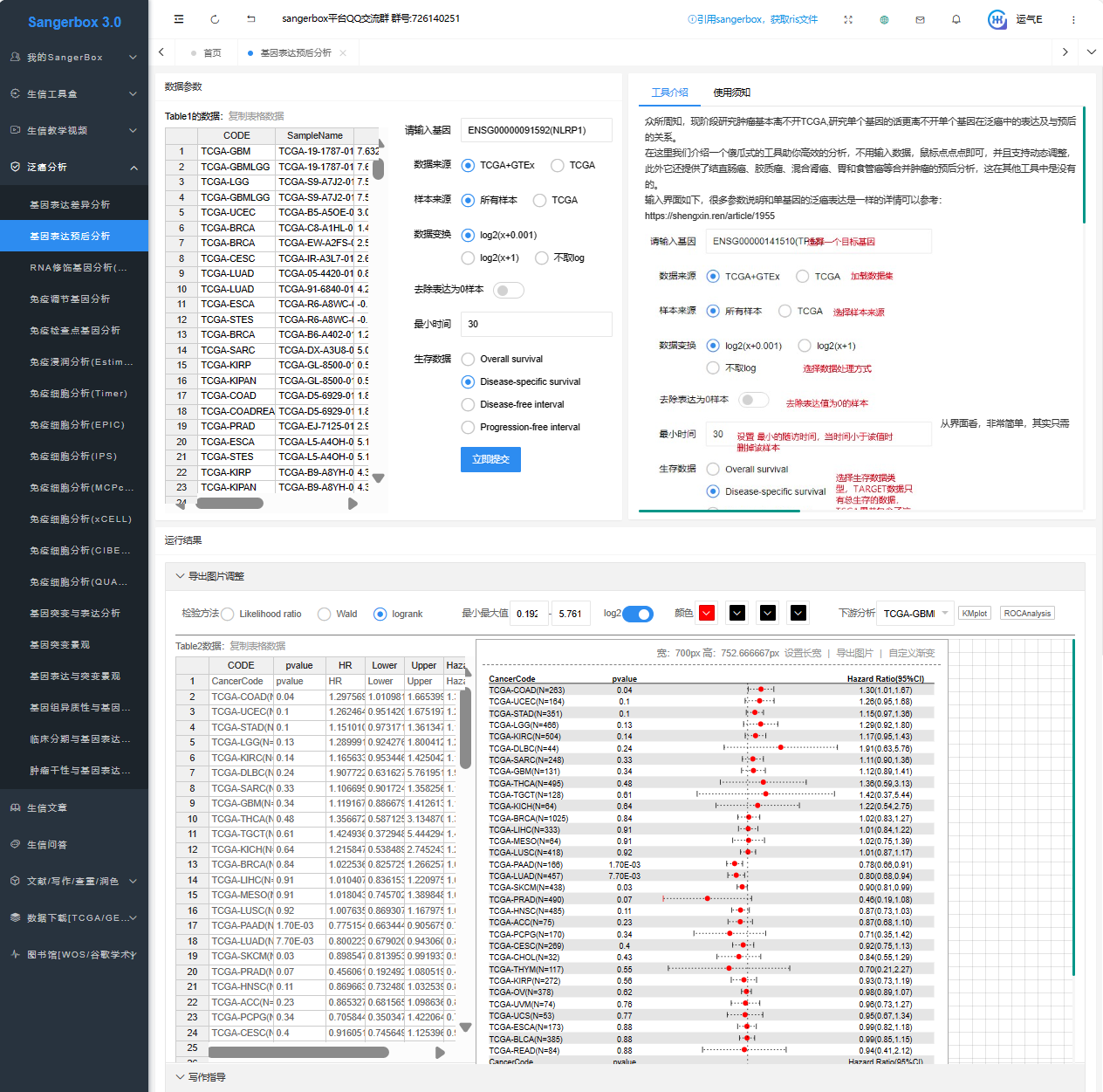
**

By following these steps, users can perform a detailed prognostic analysis of the gene of interest using both the Kaplan-Meier Plotter and Sangerbox 3.0, interpreting the significance of the gene of interest in tumor prognosis across multiple cancer types.

**3.4 Potential Implications of NLRP1 Differential Expression Across Single-cell Types (Figure 6, analyzed on May 14, 2024)**

1. Access GEPIA2021:

Navigate to the GEPIA2021 database: http://gepia2021.cancer-pku.cn/.

1. Navigate to the “Sub-Expression” Module:

On the GEPIA2021 homepage, select the “sub-expression” module.

1. Conduct Cell Type-Level Expression Analysis:

Within the “sub-expression” module, select “Cell Type-level Expression Analysis”.

1. Select Deconvolution Tool and Cell Types:

Choose “EPIC” as the deconvolution tool. Select “Macrophages” and “Endothelials” as the cell types of interest.

1. Input Gene Information:

Enter “NLRP1” in the “Gene symbol” field.

1. Select Tumor and Normal Tissue Samples for LUAD:

Under “TCGA Tumor”, select “LUAD Tumor”. Under “GTEx”, select “Lung” to obtain the expression levels of macrophages and endothelial cells in LUAD and lung tissues (**Figure 6A** (main body manuscript)).

1. Select Tumor and Normal Tissue Samples for PAAD:

Under “TCGA Tumor”, select “PAAD Tumor”. Under “GTEx”, select “Pancreas” to obtain the expression levels of macrophages and endothelial cells in PAAD and pancreas tissues (**Figure 6B** (main body manuscript)).

1. Review and Interpret the Data:

The resulting plots will display the expression levels of NLRP1 in macrophages and endothelial cells within the selected tumor and normal tissues. **Figure 6A** in the main body manuscript shows the expression comparison in LUAD and lung tissues. **Figure 6B** in the main body manuscript shows the expression comparison in PAAD and pancreas tissues. Detailed screenshots of the analysis steps are provided below to illustrate the process using the GEPIA2021.

**
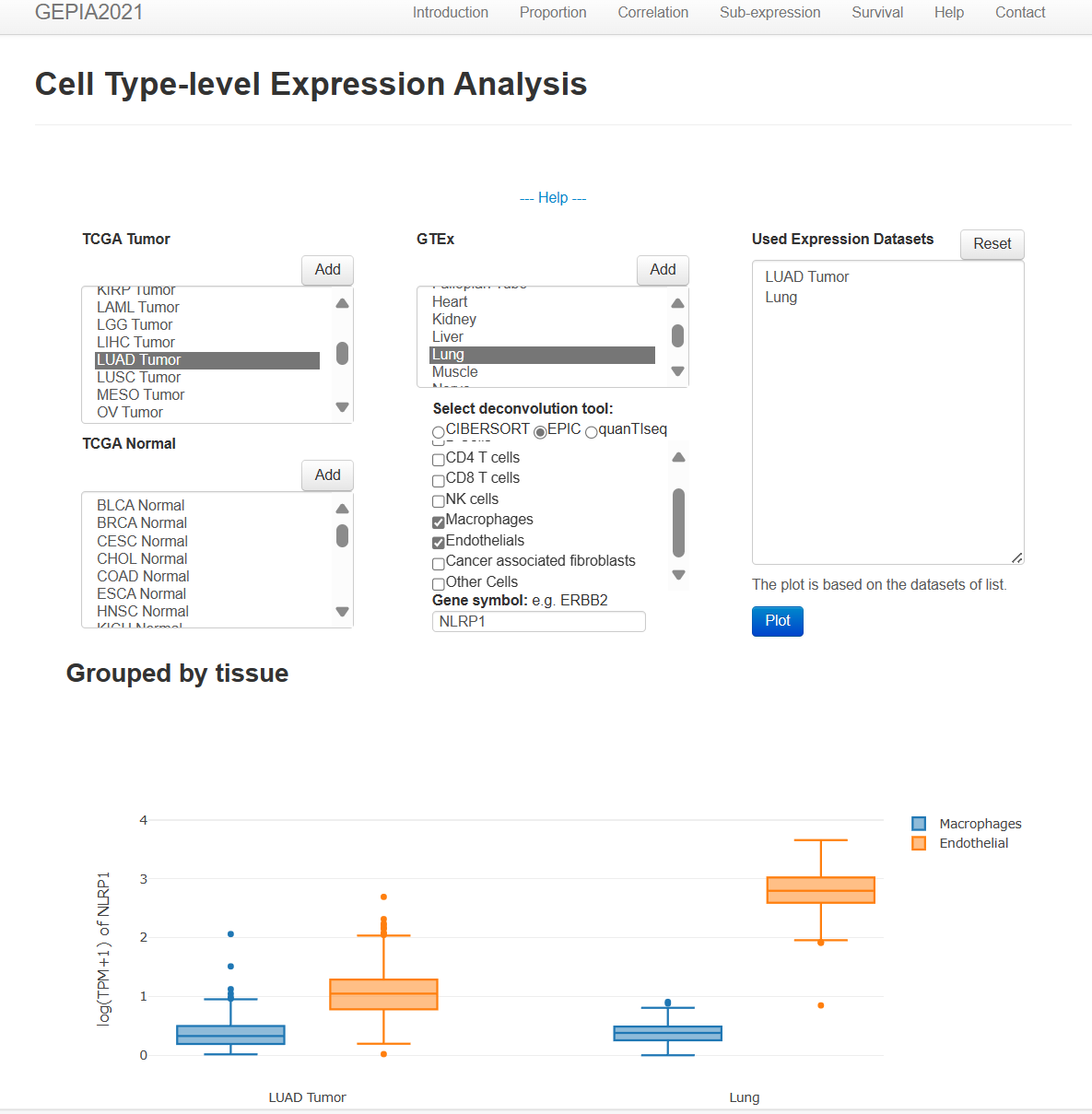
**

**
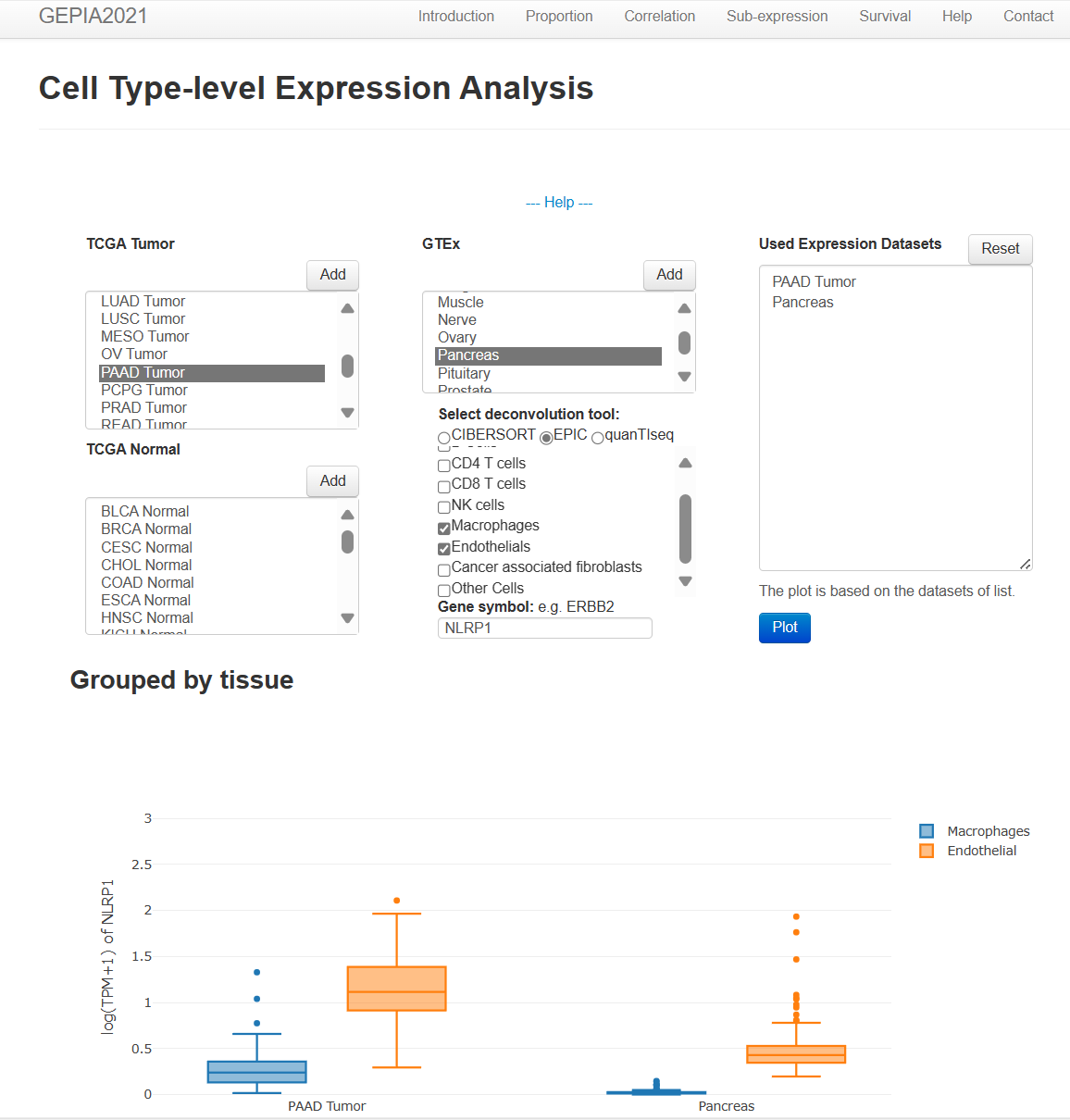
**

By following these steps, users can accurately compare the expression levels of the gene of interest in macrophages and endothelial cells across different tissues using the GEPIA2021 database.

**3.5 Association Between NLRP1 Interacting Network and Immunity Pathways (Figure 7A-E, analyzed on May 10, 2024)**

**1. Constructing the Protein-Protein Interaction Network:**

**1.1** Access the STRING Database:

Navigate to the STRING database: https://cn.string-db.org/.

**1.2** Search for NLRP1:

Select the “Protein by name” module. Enter “NLRP1” in the “Protein Name” field. Set “Organisms” to “Homo sapiens”.

**1.3** Configure Network Parameters:

Under “Network Type”, select “full STRING network”. Set the “Required score” to “medium confidence (0.400)”. Set “Size cutoff” to “no more than 20 interactors”.

**1.4** Generate and Adjust PPI Network:

Click “SEARCH” to construct the optimal protein-protein interaction (PPI) network for NLRP1. Navigate to the resulting PPI network page, adjust the network layout by dragging, and export the image (**Figure 7A** (main body manuscript)).


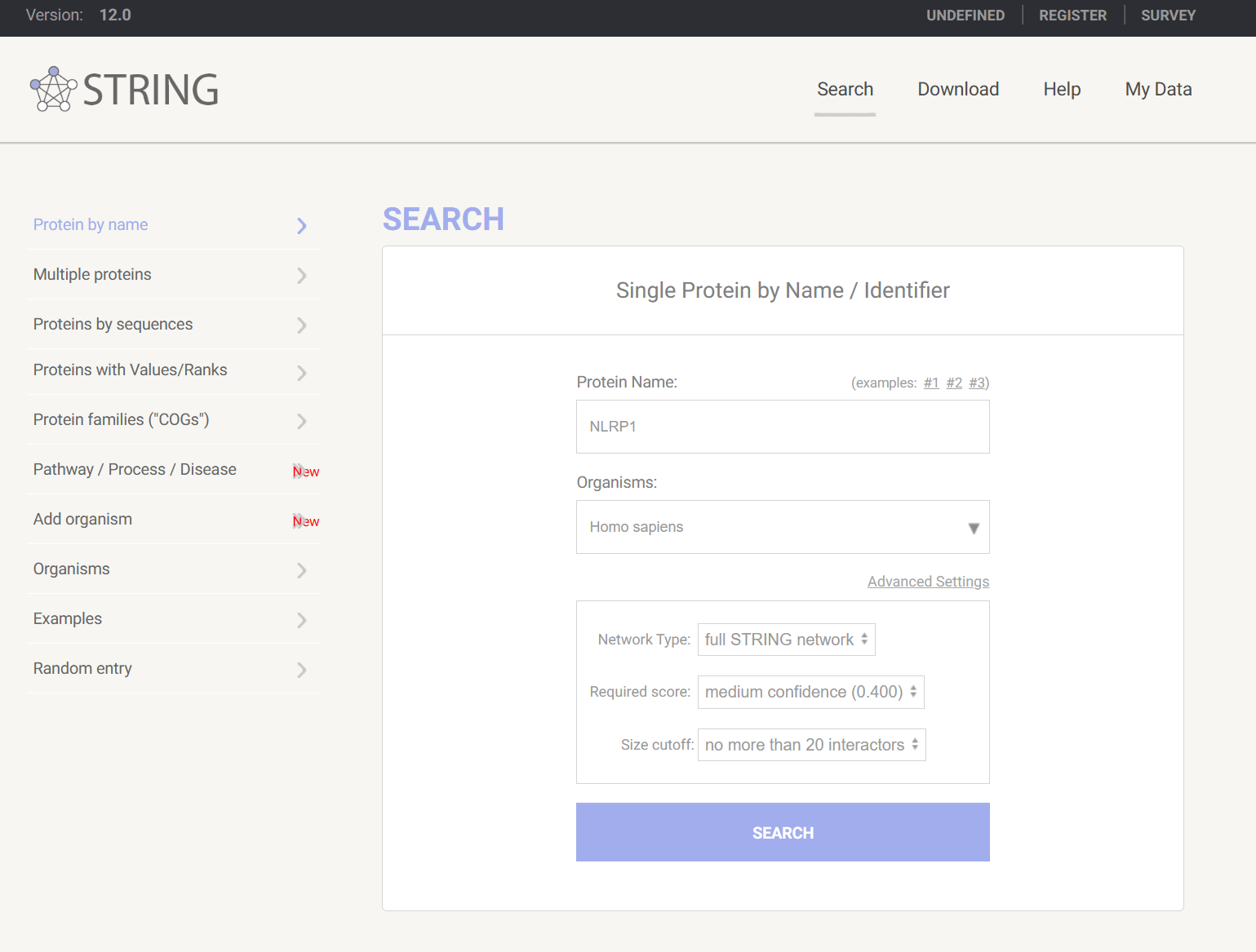


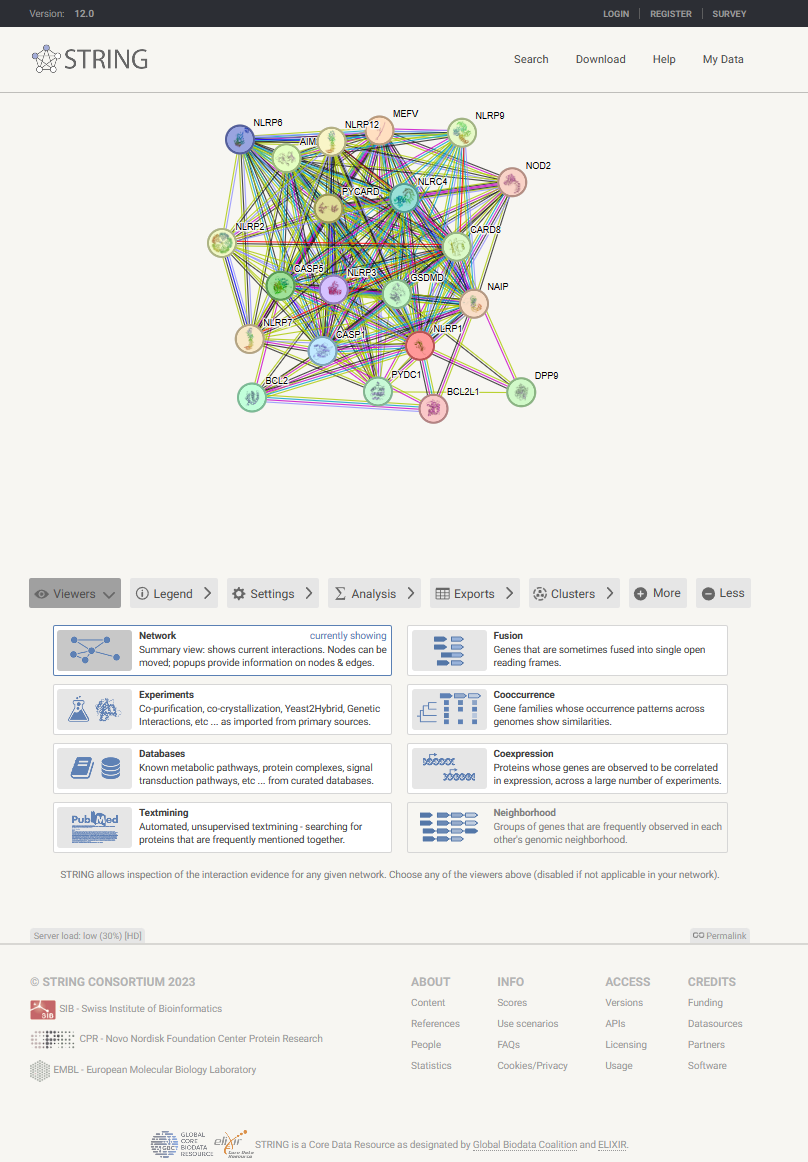


**2. Exploring the Functional Relevance of the PPI Network:**

**2.1** Access the Sangerbox 3.0 Platform:

Navigate to Sangerbox 3.0: http://vip.sangerbox.com/

**2.2** Use “Bioinformatics Analysis” Module:

Select the “GO、KEGG一键富集分析工具” (GO and KEGG Enrichment Analysis Tool).

**2.3** Import PPI Network Genes:

Import the genes from the NLRP1 PPI network into the left table of the analysis tool.

**2.4** Configure KEGG Enrichment Analysis Parameters:

Select “Benjamini & Hochberg” as the statistical method for enrichment analysis. In the “选择数据库” (Choose Database) section, select “KEGG” for the enrichment analysis method. Perform the KEGG enrichment analysis and choose “圈图” (Circular Plot) and “使用FDR” (Use FDR) for visualization (**Figure 7B** (main body manuscript)).


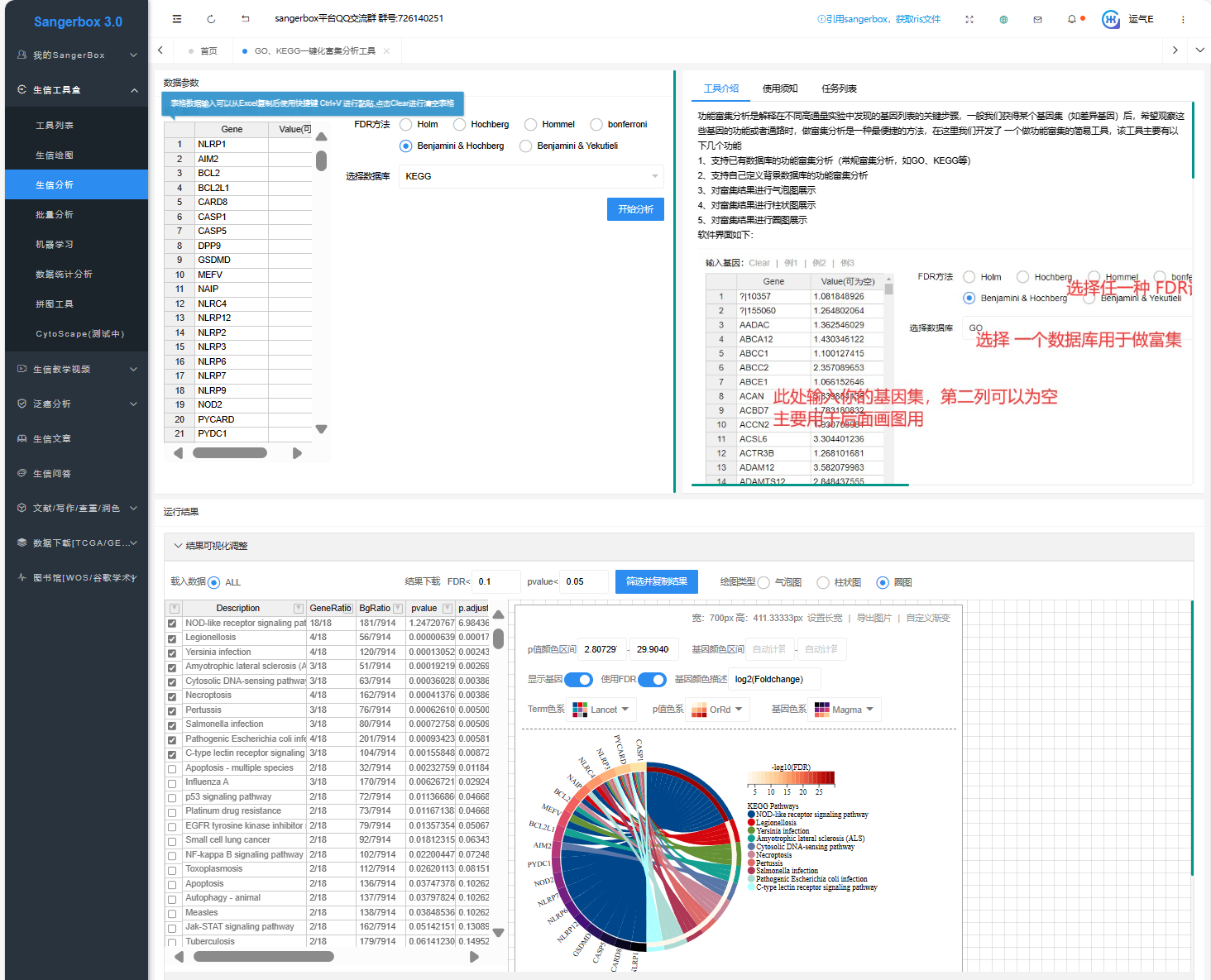


**3. Conducting Gene Ontology (GO) Enrichment Analysis:**

**3.1** Select GO Enrichment Parameters:

Within the same “GO、KEGG一键富集分析工具” module, choose “Benjamini & Hochberg” for statistical analysis of enrichment.

**3.2** Choose Databases for GO Analysis:

In the “选择数据库” (Choose Database) section: “C5: GO molecular functions”, “C5: GO cellular components”, “C5: GO biological processes”

**3.3** Set Significance Criterion:

Opt for “使用FDR” (Use FDR) as the significance criterion for visualization.

**3.4** Run the Analysis and Visualize Results:

Conduct the GO enrichment analysis and visualize the results for each category: **Figure 7C** (main body manuscript): GO Molecular Functions, **Figure 7D** (main body manuscript): GO Cellular Components, **Figure 7E** (main body manuscript): GO Biological Processes.

**4. Review and Interpret the Results:**

The visualizations (**Figure 7C-E** (main body manuscript)) provide insights into the molecular functions, cellular components, and biological processes associated with the NLRP1 interacting network.


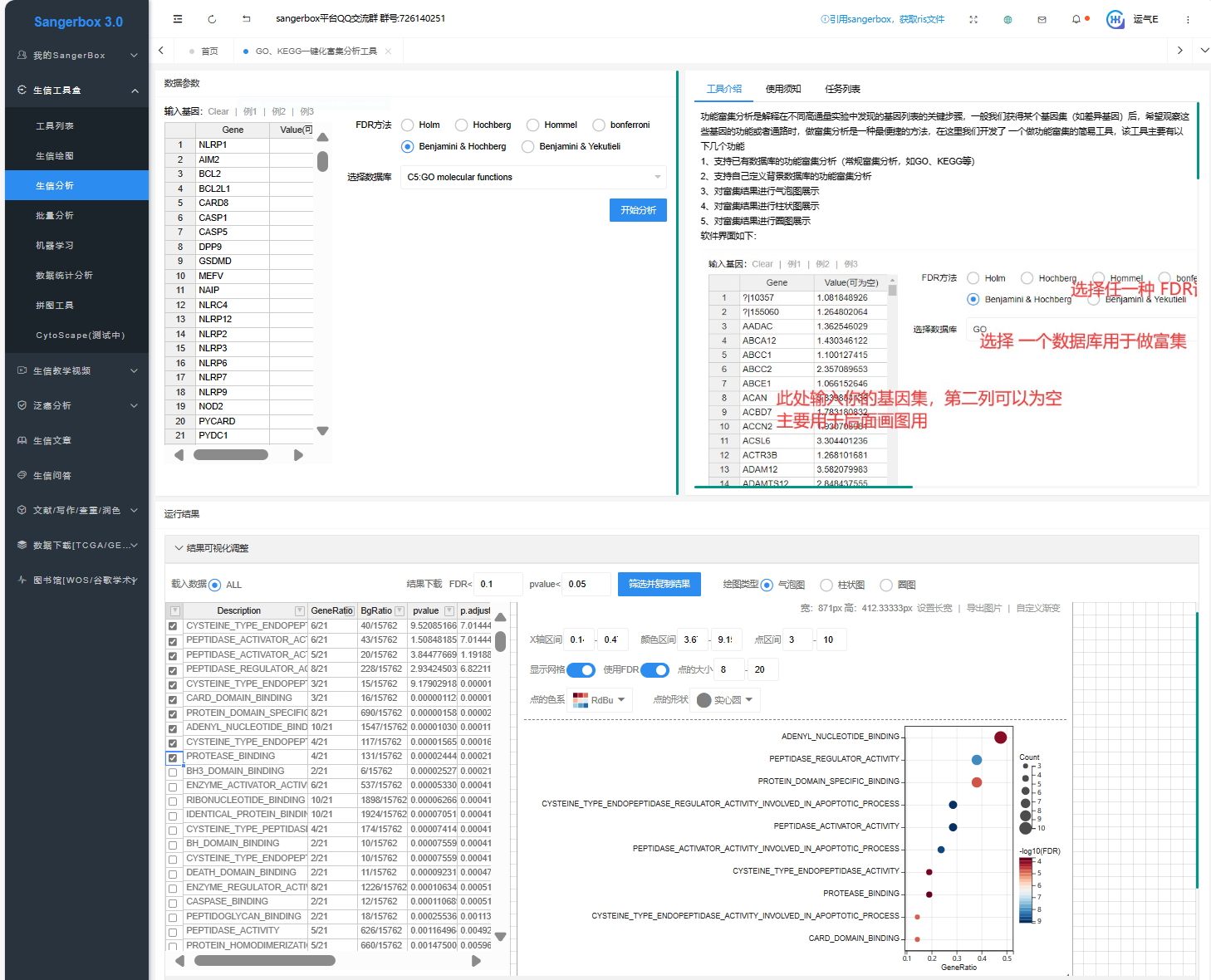


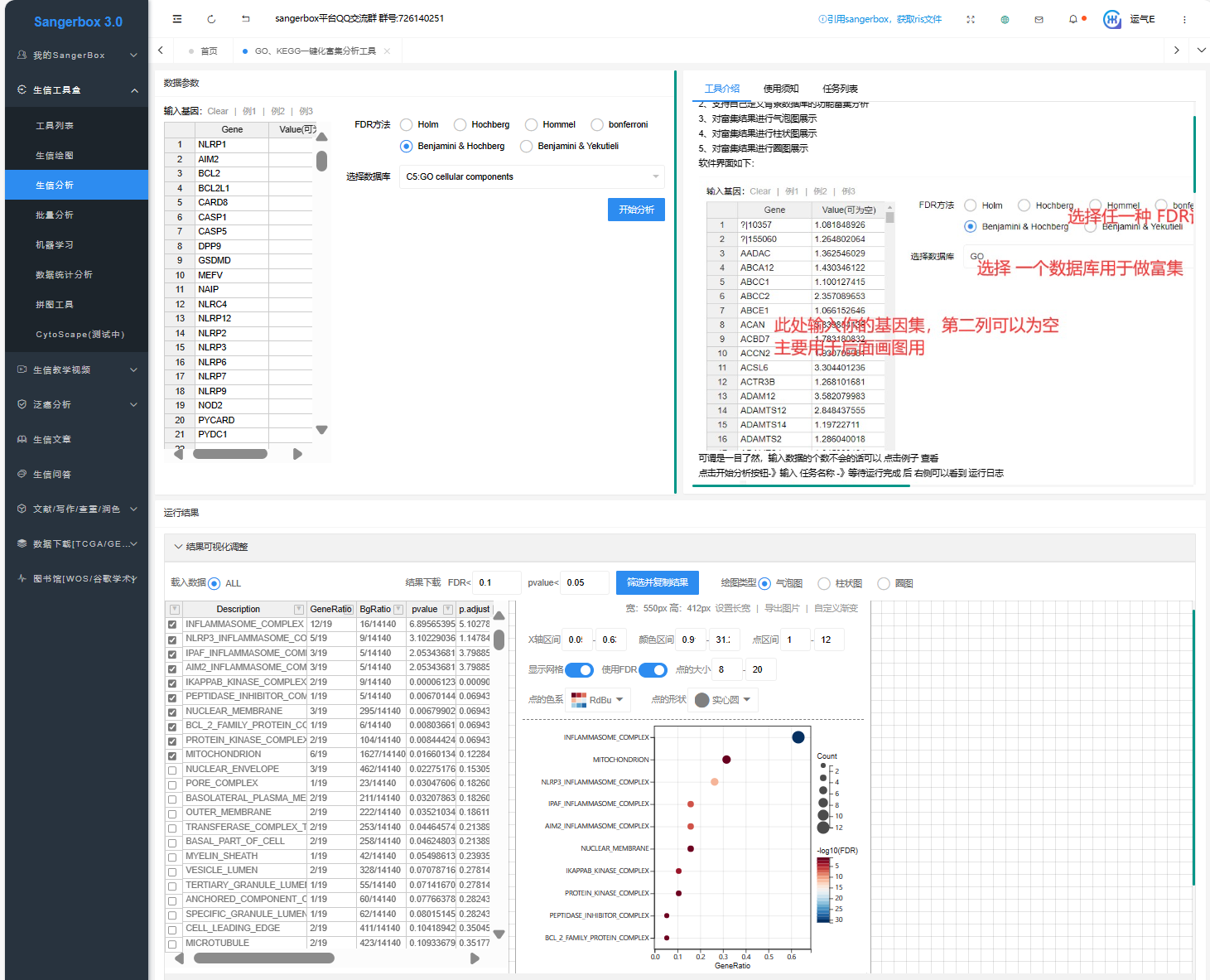


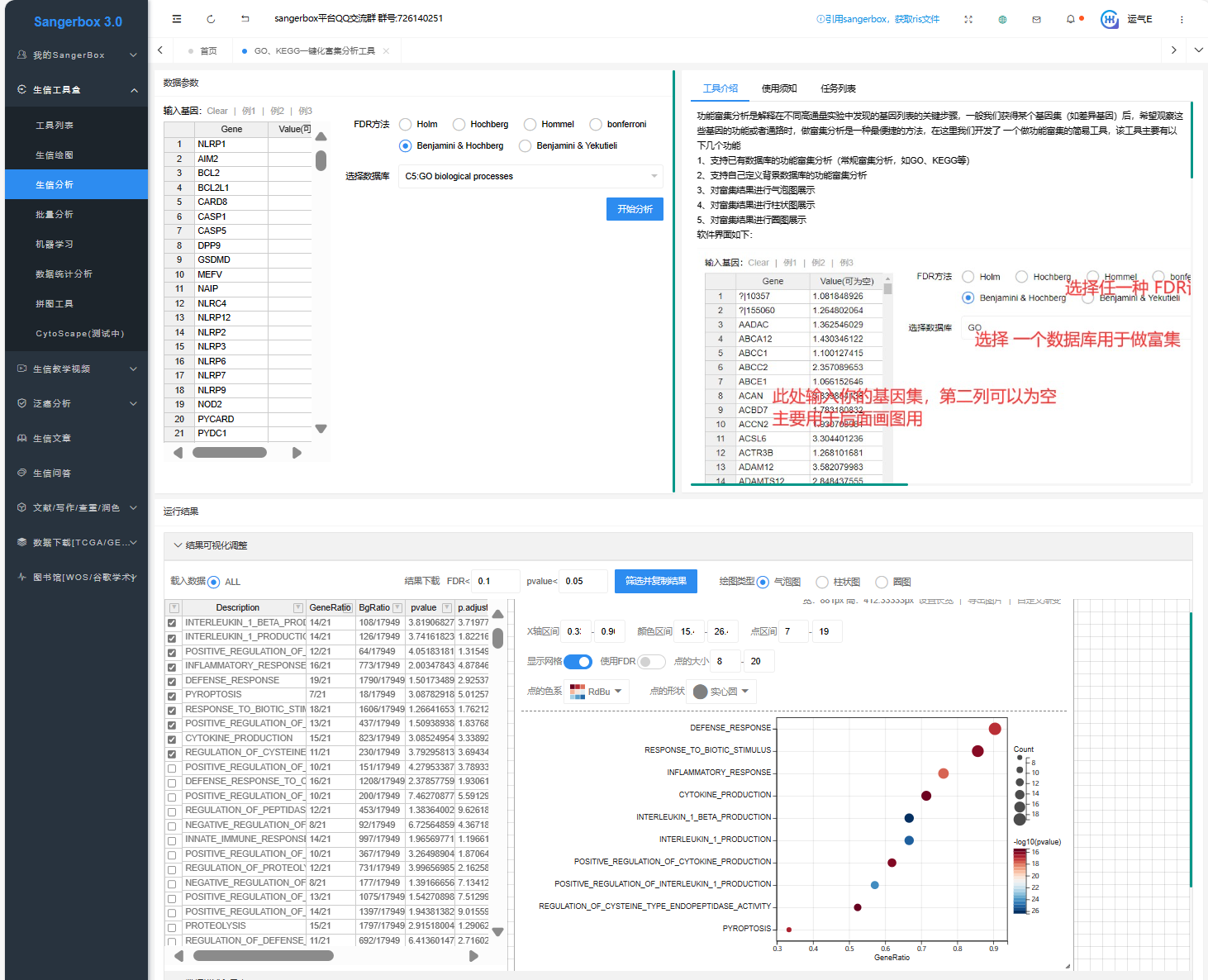


By following these steps, users can construct the protein-protein interaction network of gene of interest using the STRING database and explore its functional relevance through KEGG and GO enrichment analyses using the Sangerbox 3.0 platform.

**3.6 Negative Correlation Between NLRP1 and Tumor Stemness (Figure 7F, analyzed on May 12, 2024)**

1. Access the Sangerbox 3.0 Platform:

Navigate to Sangerbox 3.0: http://vip.sangerbox.com/

1. Navigate to the Pan-Cancer Analysis Section:

Within the Sangerbox 3.0 interface, go to the “泛癌分析” (Pan-Cancer Analysis) section.

1. Select the “Tumor Stemness and Gene Expression Analysis” Module:

Choose the “Tumor Stemness and Gene Expression Analysis” module.

1. Input Gene Information:

Enter the gene identifier “NLRP1” in the designated field.

1. Choose Data Source and Transformation Method:

Set the data source to “RNAss”. Apply the data transformation using the formula “log2(x+0.001)”.

1. Perform Correlation Analysis:

Use the Pearson correlation method to analyze the correlation between NLRP1 expression and tumor stemness (RNAss).

1. Generate Visualization:

The correlation analysis will produce a Lollipop chart illustrating the relationship between NLRP1 and RNAss (**Figure 7F** (main body manuscript)).

1. Review and Interpret the Results:

The Lollipop chart (**Figure 7F** (main body manuscript)) displays the correlation between NLRP1 expression and tumor stemness across various cancer types.

**
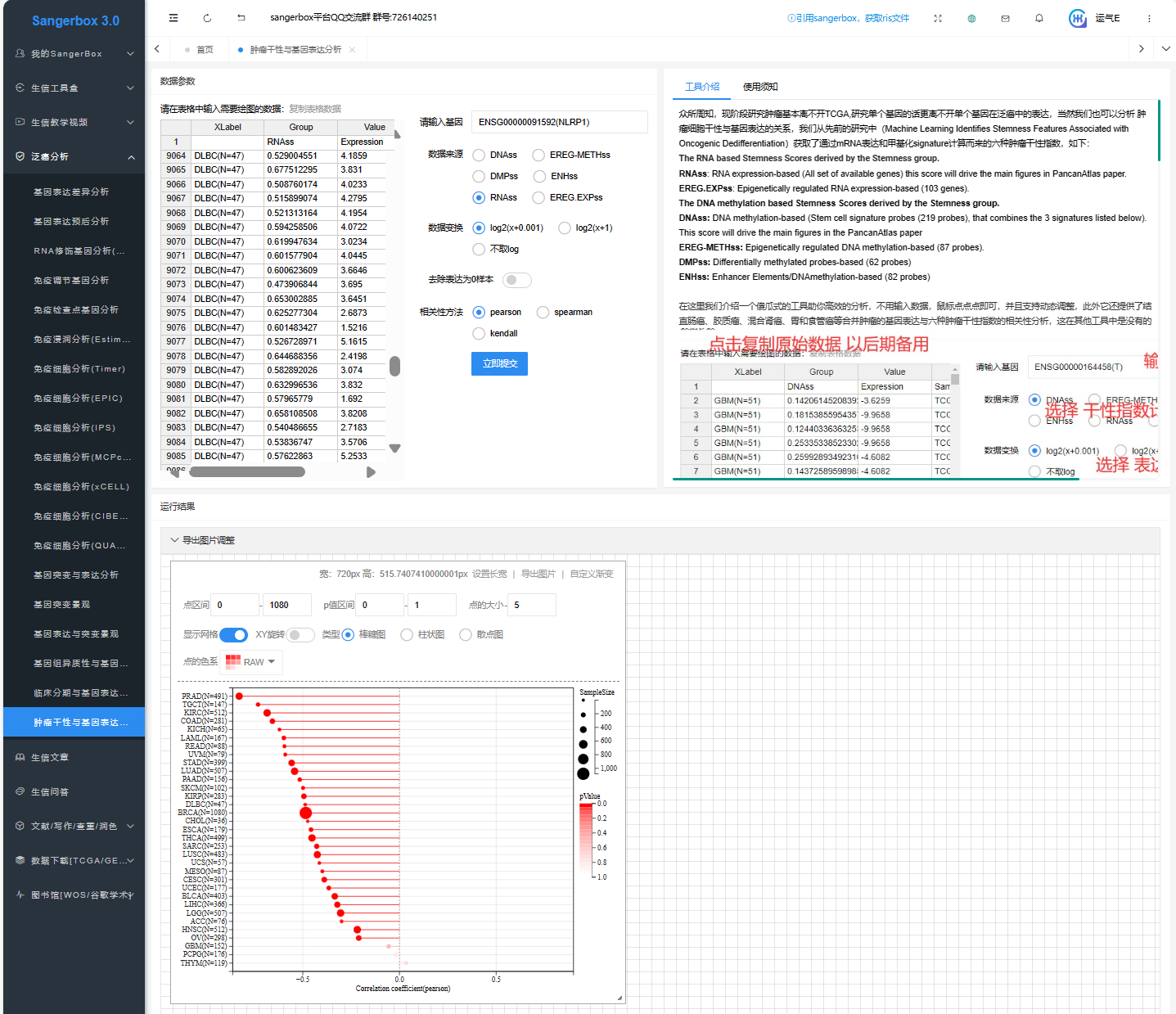
**

By following these steps, users can perform a correlation analysis between gene expression of the gene of interest and tumor stemness using the Sangerbox 3.0 platform, and visualize the results effectively.

**3.7 Positive Analysis Between NLRP1 and Immune Microenvironment (Figures 8 and 9, analyzed on May 12, 2024)**

**Guidelines for Correlation Analysis Between NLRP1 and Tumor-Infiltrating Lymphocytes Abundances Using TISIDB “Lymphocyte” Module**

1. Access the Tumor Immune System Interactions Database (TISIDB):

Navigate to the TISIDB website: http://cis.hku.hk/TISIDB/index.php

1. Retrieve Data on NLRP1:

Search for data related to the NLRP1 gene within the TISIDB database.

1. Select the “Lymphocyte” Module:

Within the TISIDB interface, choose the “Lymphocyte” module.

1. Generate Heatmap of Correlation:

Use the selected “Lymphocyte” module to generate a heatmap illustrating the correlation between NLRP1 expression and the abundances of 28 tumor-infiltrating lymphocytes (TIL) subtypes across various cancers (**Figure 8A**).

1. Review and Interpret the Heatmap:

Analyze the heatmap to understand the relationship between NLRP1 expression and the abundance of different TIL subtypes. **Figure 8A** (main body manuscript) displays the heatmap visualizing the correlation between NLRP1 and TIL abundances.


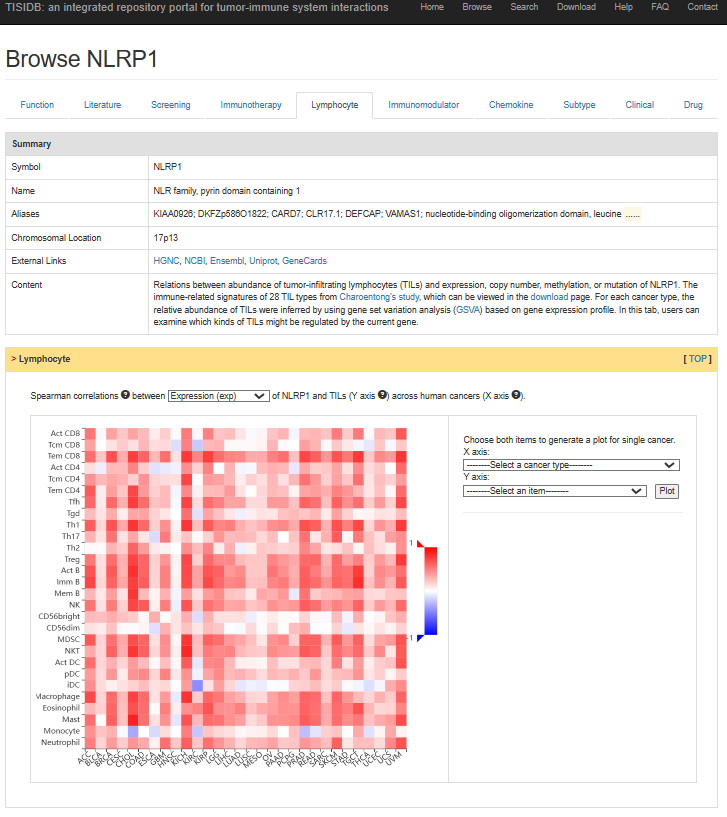


By following these steps, users can perform a correlation analysis between NLRP1 expression and tumor-infiltrating lymphocytes using the TISIDB and visualize the results through heatmaps.

**Correlation Analysis Between NLRP1 and Immune Pathway Marker Genes Using the “Immunoinhibitor” Module**

1. Access the “Immunoinhibitor” Module:

Navigate to the TISIDB website: http://cis.hku.hk/TISIDB/index.php. Select the “Immunoinhibitor” module within TISIDB.

1. Explore the Expression of Immune Pathway Marker Genes:

The “Immunoinhibitor” module contains the expression data of two types of immune pathway-related marker genes: Immunostimulatory factors and Major Histocompatibility Complex (MHC) molecules.

1. Generate Heatmaps of Correlation:

Utilize the “Immunoinhibitor” module to generate heatmaps illustrating the correlation between NLRP1 expression and the expression of MHC markers, immune-stimulating markers, and chemokine proteins. **Figure 8B** (main body manuscript): Heatmap of correlation between NLRP1 expression and MHC markers. **Figure 8C** (main body manuscript): Heatmap of correlation between NLRP1 expression and immune stimulating markers.

1. Review and Interpret the Heatmaps:

Analyze the heatmaps to understand the correlation between NLRP1 expression and the expression of different immune pathway-related marker genes. These heatmaps provide insights into the potential involvement of NLRP1 in modulating the immune response through its interaction with various immune pathway markers. Below are the screenshots illustrating the steps involved in this analysis.

**
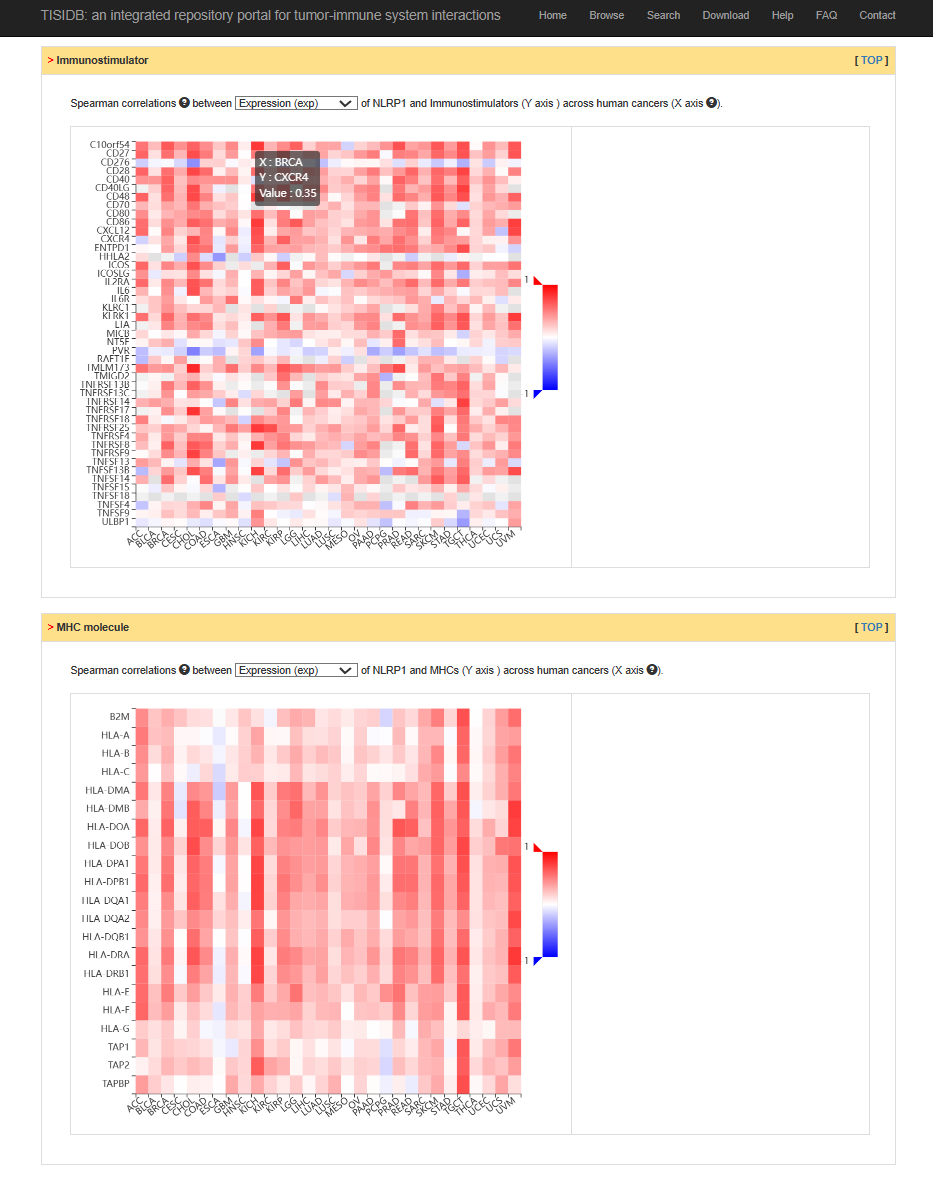
**

**Correlation Analysis Between NLRP1 and Chemokine Proteins Using the “Chemokine” Module**

1. Access the TISIDB “Chemokine” Module:

Navigate to the TISIDB website: http://cis.hku.hk/TISIDB/index.php. Select the “Chemokine” module within TISIDB.

1. Explore the Expression of Chemokine Proteins:

The “Chemokine” module contains the expression data of 41 chemokine proteins as immune pathway-related marker genes.

1. Generate Heatmaps of Correlation:

Utilize the “Chemokine” module to generate heatmaps illustrating the correlation between NLRP1 expression and chemokine proteins. **Figure 8D** (main body manuscript): Heatmap of correlation between NLRP1 expression and chemokine proteins.

1. Review and Interpret the Heatmaps:

Analyze the heatmaps to understand the correlation between NLRP1 expression and the expression of chemokine proteins. These heatmaps provide insights into the potential involvement of NLRP1 in modulating the immune response through its interaction with 41 chemokine proteins. Below are the screenshots illustrating the steps involved in this analysis.


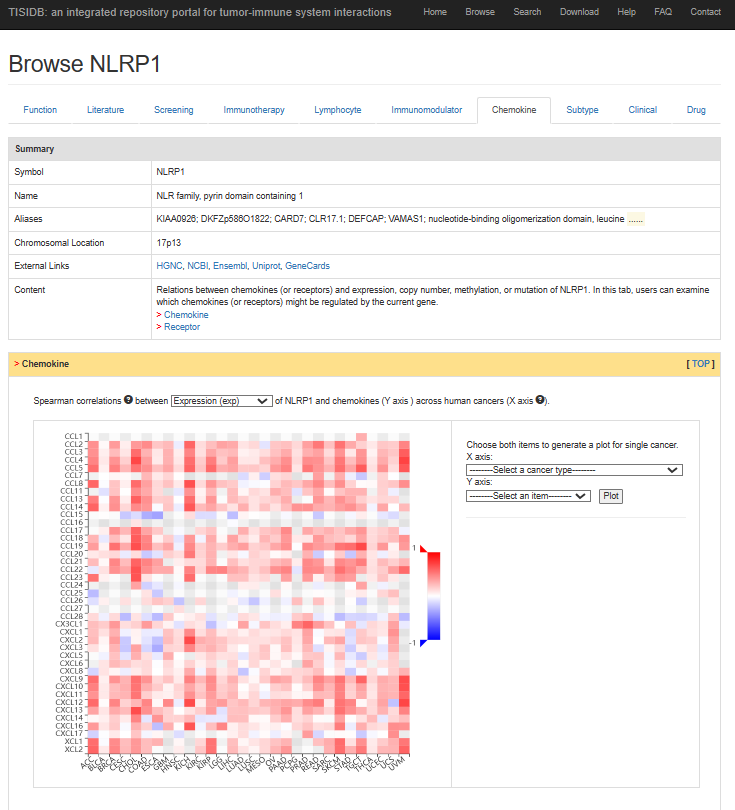


By following these steps, users can effectively analyze the correlation between NLRP1 expression and the expression of immune pathway-related marker genes using the “Immunoinhibitor” and “Chemokine” modules of TISIDB and visualize the results through heatmaps.

**Guidelines for Correlation Analysis Between NLRP1 and Immune Subtypes Using TISIDB “Subtype” Module**

1. Access the TISIDB “Subtype” Module:

Navigate to the TISIDB website: http://cis.hku.hk/TISIDB/index.php. Select the “Subtype” module from the TISIDB interface.

1. Perform Kruskal-Wallis Test:

Use the “Subtype” module to perform the Kruskal-Wallis Test to investigate the differences in the expression of NLRP1 in the immune subtypes of various cancers.

1. Obtain Bar Graphs:

Utilize the “Subtype” module to obtain bar graphs illustrating the differences in the expression of NLRP1 in the immune subtypes of various cancers (**Figure 9A** (main body manuscript)).

1. Select Specific Cancer Types:

Choose specific cancer types of interest, such as BRCA (Breast invasive carcinoma), LUAD (Lung adenocarcinoma), PRAD (Prostate adenocarcinoma), PCPG (Pheochromocytoma and Paraganglioma), and KIRC (Kidney renal clear cell carcinoma).

1. Generate Violin Plots:

Select the aforementioned cancer types to obtain violin plots depicting the immunosubtype expression of NLRP1 in these cancers (**Figure 9B-F** (main body manuscript)).


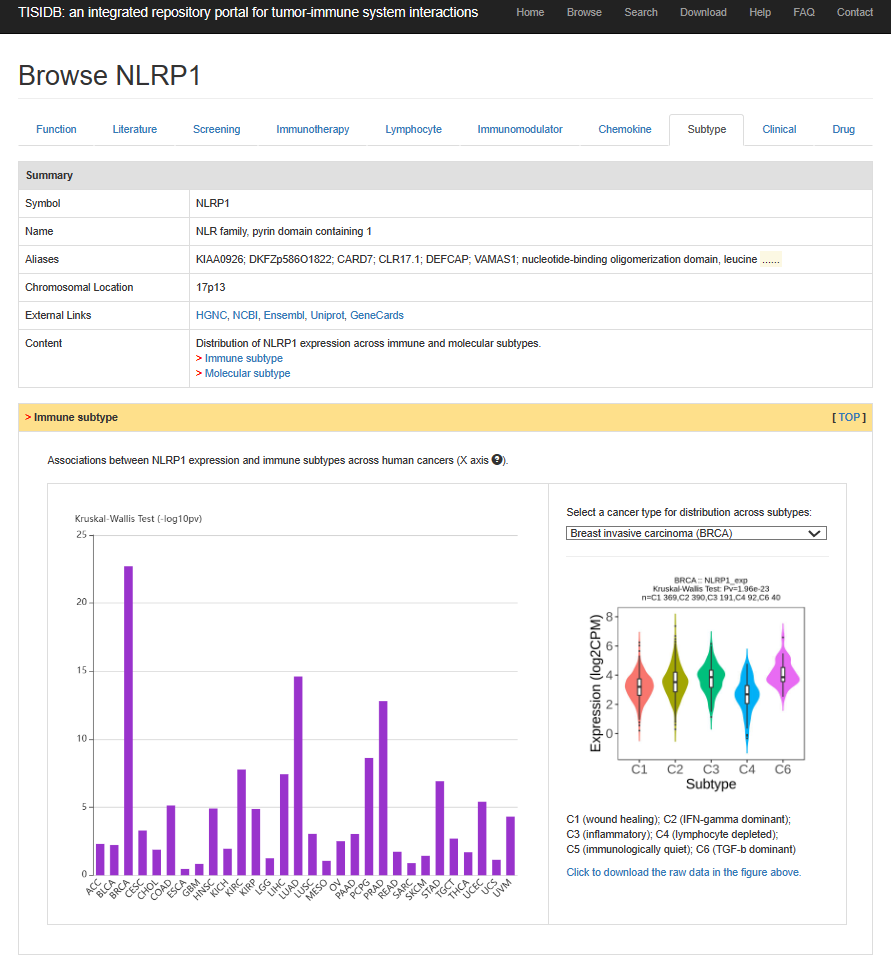


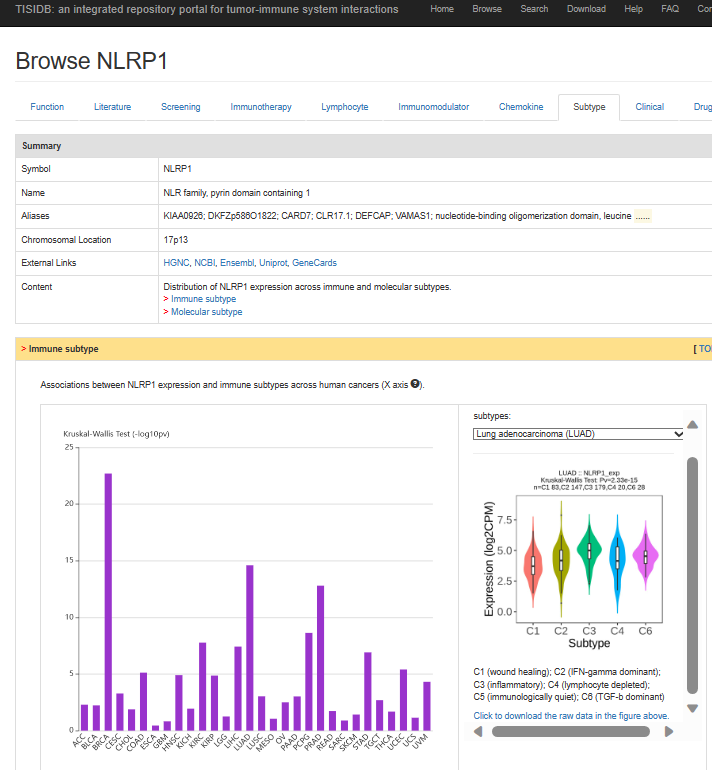


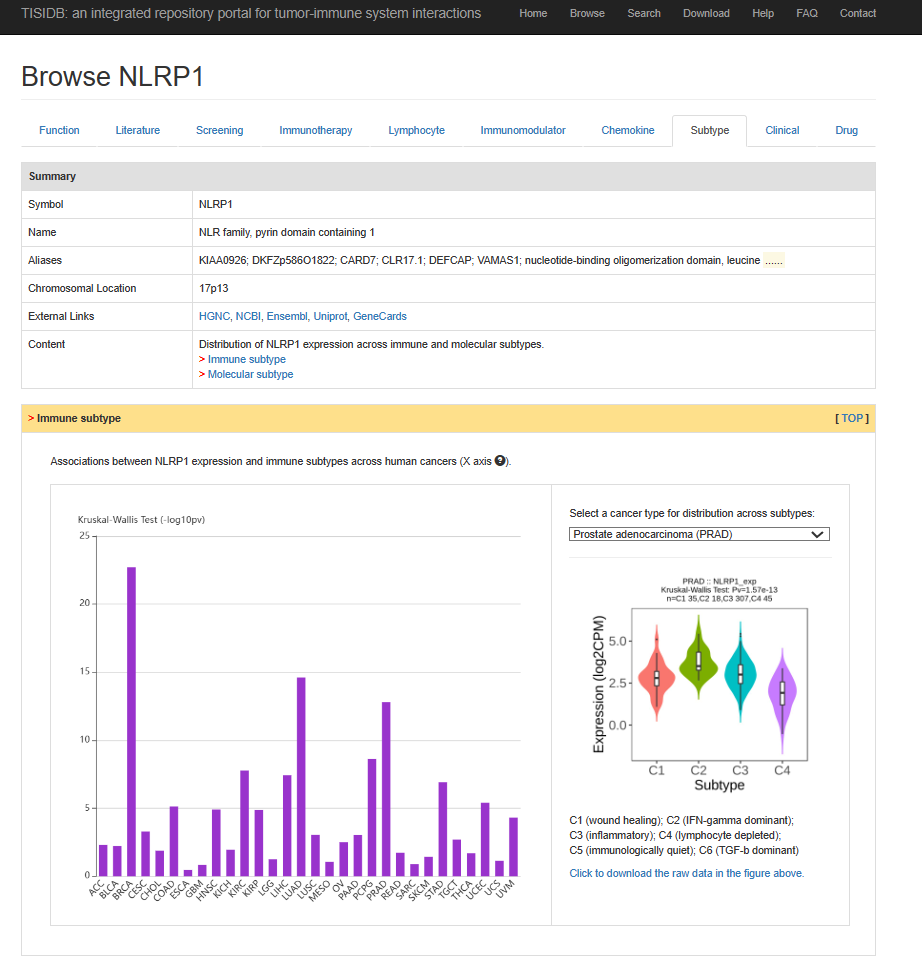


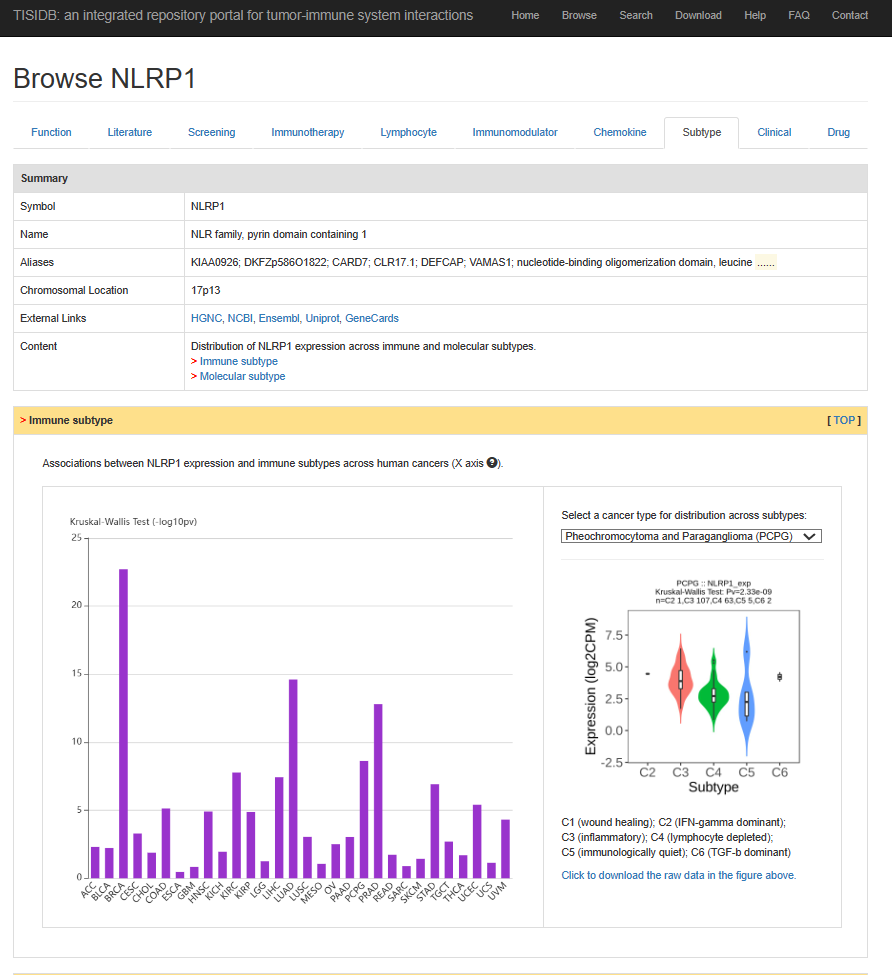


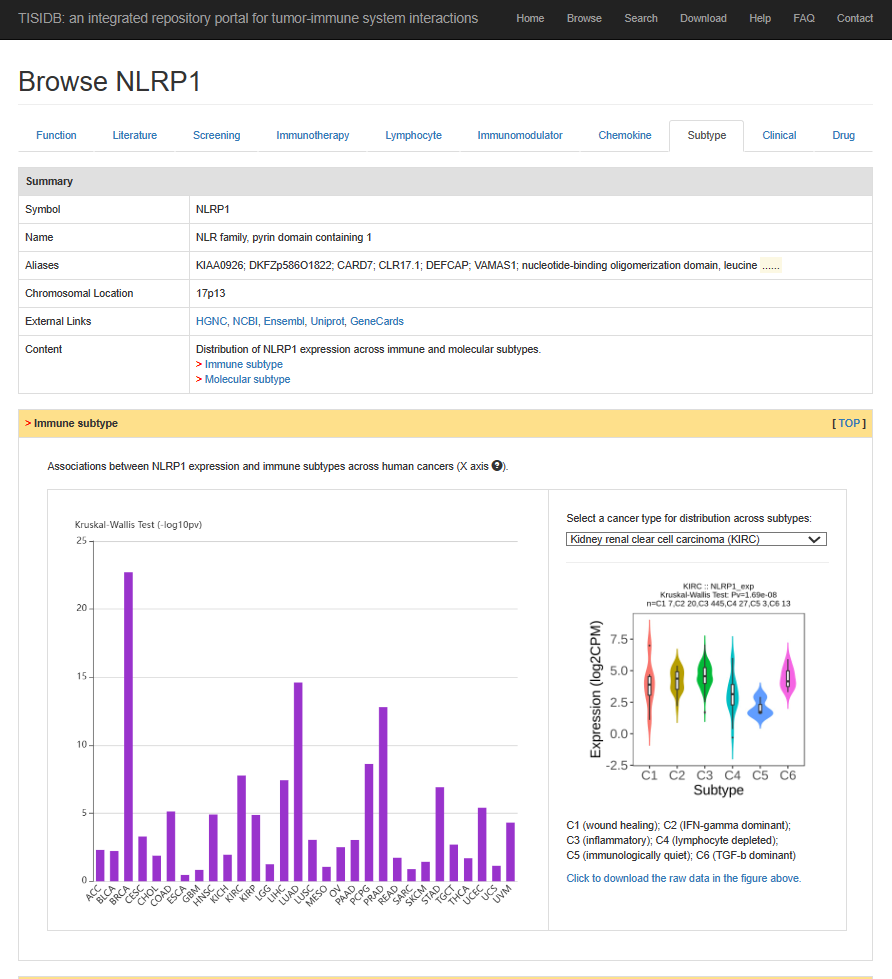


By following these guidelines, users can effectively investigate the correlation between gene expression of the gene of interest and immune subtypes using the TISIDB “Subtype” module. This analysis provides valuable insights into the role of the gene of interest in modulating immune responses across different cancer types.

**3.8 Association Between NLRP1 And Immune/Metastasis-related Pathways (Figure 10, analyzed on May 9, 2024)**

Enrichment of potential biological functional pathways in the NLRP1 high and low expression groups of individual cancers is assessed using R based on the expression profiles of 33 cancers and hallmark gene sets which are acquired from MSigDB using the file “h.all.v7.2.symbols.gmt”. The code is also detailed in the link: https://github.com/liaoyong0668/NLRP1-in-pancancer. Download and run the provided R script to generate the bubble chart for showing association of NLRP1 expression with signaling pathways across 33 cancer types (**Figure 10** (main body manuscript)).

**R code:**

library(clusterProfiler)

library(limma)

library(ggplot2)

library(data.table)

library(ggpubr)

library(GSVA)

samAnno <- read.table("easy_input_sample_annotation.txt", sep = "\t",header = T, check.names = F)

expr <- fread("geneExp.tsv",sep = "\t",stringsAsFactors = F,check.names = F,header = T)

expr <- as.data.frame(expr); rownames(expr) <- expr[,1]; expr <- expr[,-1]

gene <- sapply(strsplit(rownames(expr),"|",fixed = T), "[",1)

expr$gene <- gene

expr <- expr[!duplicated(expr$gene),]

rownames(expr) <- expr$gene; expr <- expr[,-ncol(expr)]

twoclasslimma <- function(subtype = NULL,

featmat = NULL,

treatVar = NULL,

ctrlVar = NULL,

prefix = NULL,

overwt = FALSE,

sort.p = TRUE,

verbose = TRUE,

res.path = getwd()) {

library(limma)

if(!is.element("condition", colnames(subtype))) {

stop("argument of subtype must contain a column named with 'condition'!")

}

# Create comparison list for differential analysis between two classes.

createList <- function(subtype = NULL) {

tumorsam <- rownames(subtype)

sampleList <- list()

treatsamList <- list()

treatnameList <- c()

ctrlnameList <- c()

sampleList[[1]] <- tumorsam

treatsamList[[1]] <- intersect(tumorsam, rownames(subtype[which(subtype$condition == treatVar),,drop = F]))

treatnameList[1] <- treatVar

ctrlnameList[1] <- ctrlVar

return(list(sampleList, treatsamList, treatnameList, ctrlnameList))

}

complist <- createList(subtype = subtype)

sampleList <- complist[[1]]

treatsamList <- complist[[2]]

treatnameList <- complist[[3]]

ctrlnameList <- complist[[4]]

allsamples <- colnames(featmat)

# log transformation

if(max(featmat) < 25 | (max(featmat) >= 25 & min(featmat) < 0)) {

message("--feature matrix seems to have been standardised (z-score or log transformation), no more action will be performed.")

gset <- featmat

}

if(max(featmat) >= 25 & min(featmat) >= 0){

message("--log2 transformation done for feature matrix.")

gset <- log2(featmat + 1)

}

options(warn = 1)

for (k in 1:length(sampleList)) {

samples <- sampleList[[k]]

treatsam <- treatsamList[[k]]

treatname <- treatnameList[k]

ctrlname <- ctrlnameList[k]

compname <- paste(treatname, "_vs_", ctrlname, sep="")

tmp <- rep("others", times = length(allsamples))

names(tmp) <- allsamples

tmp[samples] <- "control"

tmp[treatsam] <- "treatment"

if(!is.null(prefix)) {

outfile <- file.path(res.path, paste(prefix, "_limma_test_result.", compname, ".txt", sep = ""))

} else {

outfile <- file.path(res.path, paste("limma_test_result.", compname, ".txt", sep = ""))

}

if (file.exists(outfile) & (overwt == FALSE)) {

cat(paste0("limma of ",compname, " exists and skipped...\n"))

next

}

pd <- data.frame(Samples = names(tmp),

Group = as.character(tmp),

stringsAsFactors = FALSE)

design <-model.matrix(~ -1 + factor(pd$Group, levels = c("treatment","control")))

colnames(design) <- c("treatment","control")

fit <- limma::lmFit(gset, design = design);

contrastsMatrix <- limma::makeContrasts(treatment - control, levels = c("treatment", "control"))

fit2 <- limma::contrasts.fit(fit, contrasts = contrastsMatrix)

fit2 <- limma::eBayes(fit2, 0.01)

resData <- limma::topTable(fit2, adjust = "fdr", sort.by = "B", number = 100000)

resData <- as.data.frame(subset(resData, select=c("logFC","t","B","P.Value","adj.P.Val")))

resData$id <- rownames(resData)

colnames(resData) <- c("log2fc","t","B","pvalue","padj","id")

resData$fc <- 2^resData$log2fc

if(sort.p) {

resData <- resData[order(resData$padj),]

} else {

resData <- as.data.frame(resData)

}

if(verbose) {

resData <- resData[,c("id","fc","log2fc","pvalue","padj")]

} else {

resData <- resData[,c("id","fc","log2fc","t","B","pvalue","padj")]

}

write.table(resData, file = outfile, row.names = FALSE, sep = "\t", quote = FALSE)

cat(paste0("limma of ",compname, " done...\n"))

}

options(warn = 0)

}

es <- log2(expr[rownames(expr) == "NLRP1",] + 0.01)

msigdb.hallmark <- read.gmt("h.all.v7.2.symbols.gmt")

pct <- 0.3

gseaTab <- NULL

tumors <- c("ACC","BLCA","BRCA","CESC","CHOL","COAD", #6

"DLBC","ESCA","GBM","HNSC","KICH","KIRC",

"KIRP","LAML","LGG","LIHC","LUAD","LUSC",

"MESO","OV","PAAD","PCPG","PRAD","READ",

"SARC","SKCM","STAD","TGCT","THCA","THYM",

"UCEC","UCS","UVM")

for (i in tumors) {

message("--",i,"...")

sam <- samAnno[which(samAnno$`cancer type` == i),"simple_barcode"]

comsam <- intersect(colnames(es), sam)

tumsam <- comsam[substr(comsam,14,14) == "1"]

es_subset <- as.data.frame(t(es[,tumsam]))

es_subset <- es_subset[order(es_subset$NLRP1,decreasing = T),,drop = F]

high.es <- rownames(es_subset[1:(nrow(es_subset) * pct),,drop = F]) # 取前30%为高???

low.es <- rownames(es_subset[nrow(es_subset):(nrow(es_subset) - nrow(es_subset) * pct + 1),,drop = F])

subt <- data.frame(condition = rep(c("high","low"),c(length(high.es),length(low.es))),

row.names = c(high.es, low.es),

stringsAsFactors = F)

gset <- log2(na.omit(expr[,rownames(subt)]) + 1)

twoclasslimma(subtype = subt,

featmat = gset,

treatVar = "high",

ctrlVar = "low",

prefix = paste0("TCGA_enrichment_score_",i),

overwt = T,

sort.p = F,

verbose = TRUE,

res.path = ".")

res <- read.table( paste0("TCGA_enrichment_score_",i,"_limma_test_result.high_vs_low.txt"), sep = "\t",row.names = 1,check.names = F,stringsAsFactors = F,header = T)

res <- res[order(res$log2fc, decreasing = T),]

glist <- res$log2fc; names(glist) <- rownames(res)

set.seed(20211114)

gsea <- GSEA(geneList = glist,

pvalueCutoff = 1,

seed = TRUE,

TERM2GENE = msigdb.hallmark)

gc()

gsea.df <- as.data.frame(gsea)

write.table(gsea.df,file = "output_gsea between high and low group of enrichment score in pancancer.txt",sep = "\t",row.names = T,col.names = NA,quote = F)

gseaTab <- rbind.data.frame(gseaTab,

data.frame(term = gsea.df$ID,

NES = gsea.df$NES,

FDR = gsea.df$p.adjust,

tumor = i,

stringsAsFactors = F),

stringsAsFactors = F)

}

darkblue <- "#303B7F"

darkred <- "#D51113"

yellow <- "#EECA1F"

tmp <- gseaTab

tmp$term <- gsub("HALLMARK_","",tmp$term)

my_palette <- colorRampPalette(c(darkblue,yellow,darkred), alpha=TRUE)(n=128)

ggplot(tmp, aes(x=tumor,y=term)) +

geom_point(aes(size=-log10(FDR),color=NES)) +

scale_color_gradientn('NES',

colors=my_palette) +

theme_bw() +

theme(panel.grid.minor = element_blank(),

panel.grid.major = element_blank(),

axis.text.x = element_text(angle = 45, size = 12, hjust = 0.3, vjust = 0.5, color = "black"),

axis.text.y = element_text(size = 10, color = "black"),

axis.title = element_blank(),

panel.border = element_rect(size = 0.7, linetype = "solid", colour = "black"),

legend.position = "bottom",

plot.margin = unit(c(1,1,1,1), "lines"))

**Result:**


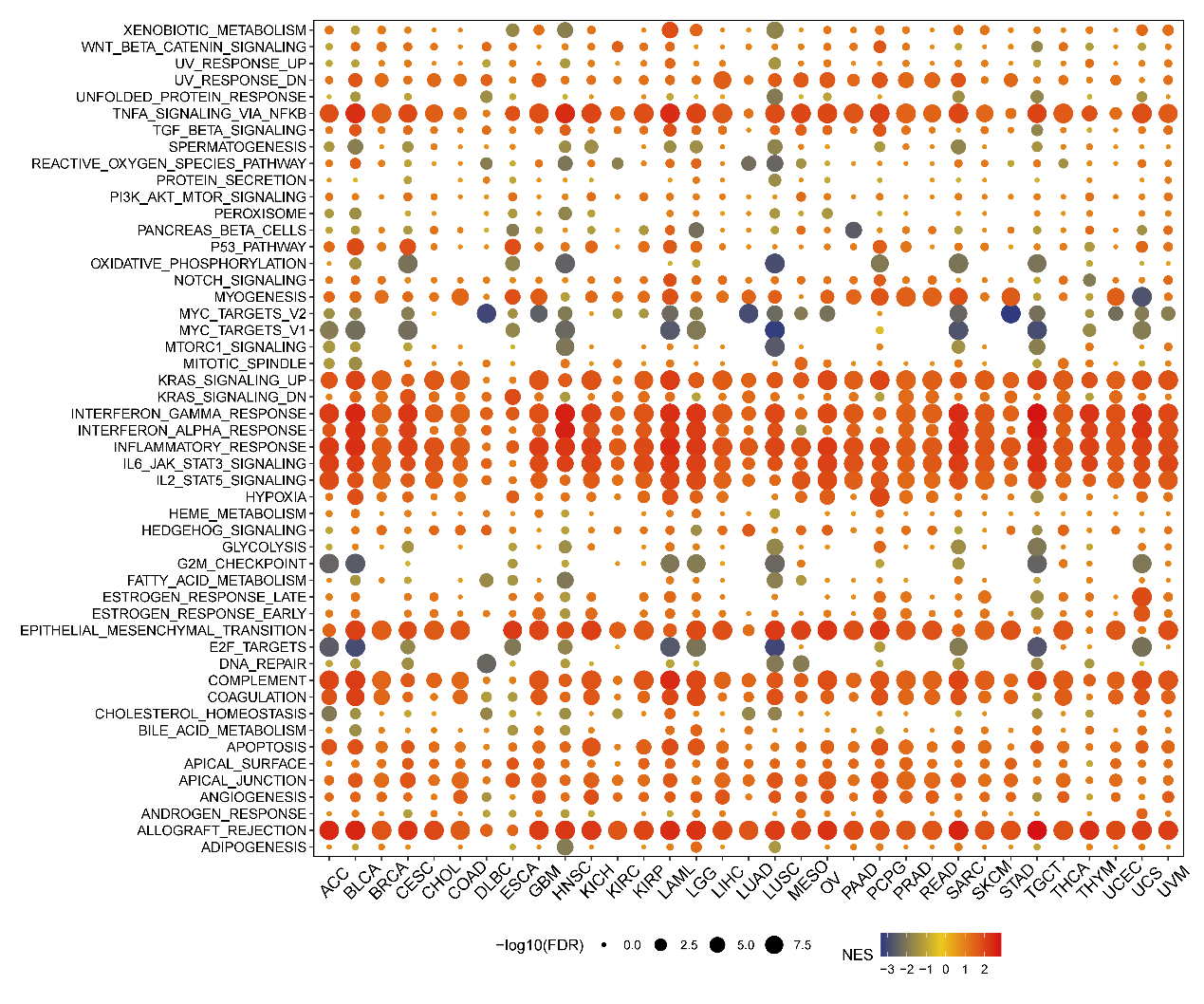


**3.9 Potential Association Between NLRP1 and Tumor Metabolic Reprogramming (Figure 11, analyzed on May 13, 2024)**

1. Access GEPIA2 Platform:

Go to the GEPIA2 website: http://gepia2.cancer-pku.cn/.

1. Select “Correlation Analysis” Function:

Within the “Expression Analysis” module, choose the “Correlation Analysis” function.

1. Input Gene and Gene Set:

Under “Gene A”, input the NLRP1 gene. In the second section, select “Signatures” and input the gene set for glycolysis, fatty acid metabolism, and amino acid metabolism obtained from the Molecular Signatures Database (MSigDB) under “Gene Set B”.

1. Choose Cancer Type and Sample Type:

Select “LUAD Tumor” and “LUAD Normal” to obtain gene expression data for tumor samples and normal samples of Lung Adenocarcinoma (LUAD).

1. Analyze and Visualize Correlation:

Click on “plot” to perform the correlation analysis and visualize the correlation between NLRP1 and glycolysis, fatty acid metabolism, and amino acid metabolism (**Figure 11** (main body manuscript)).


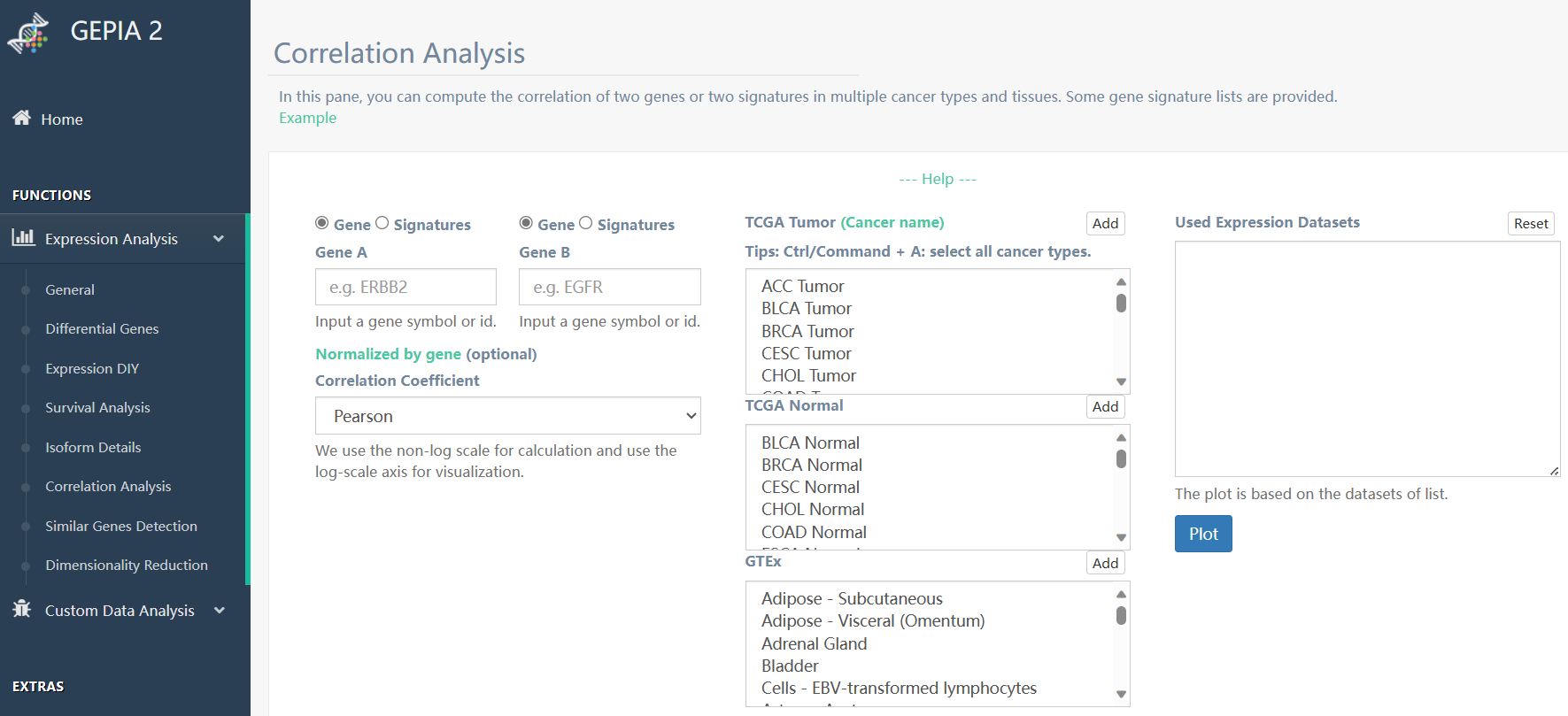


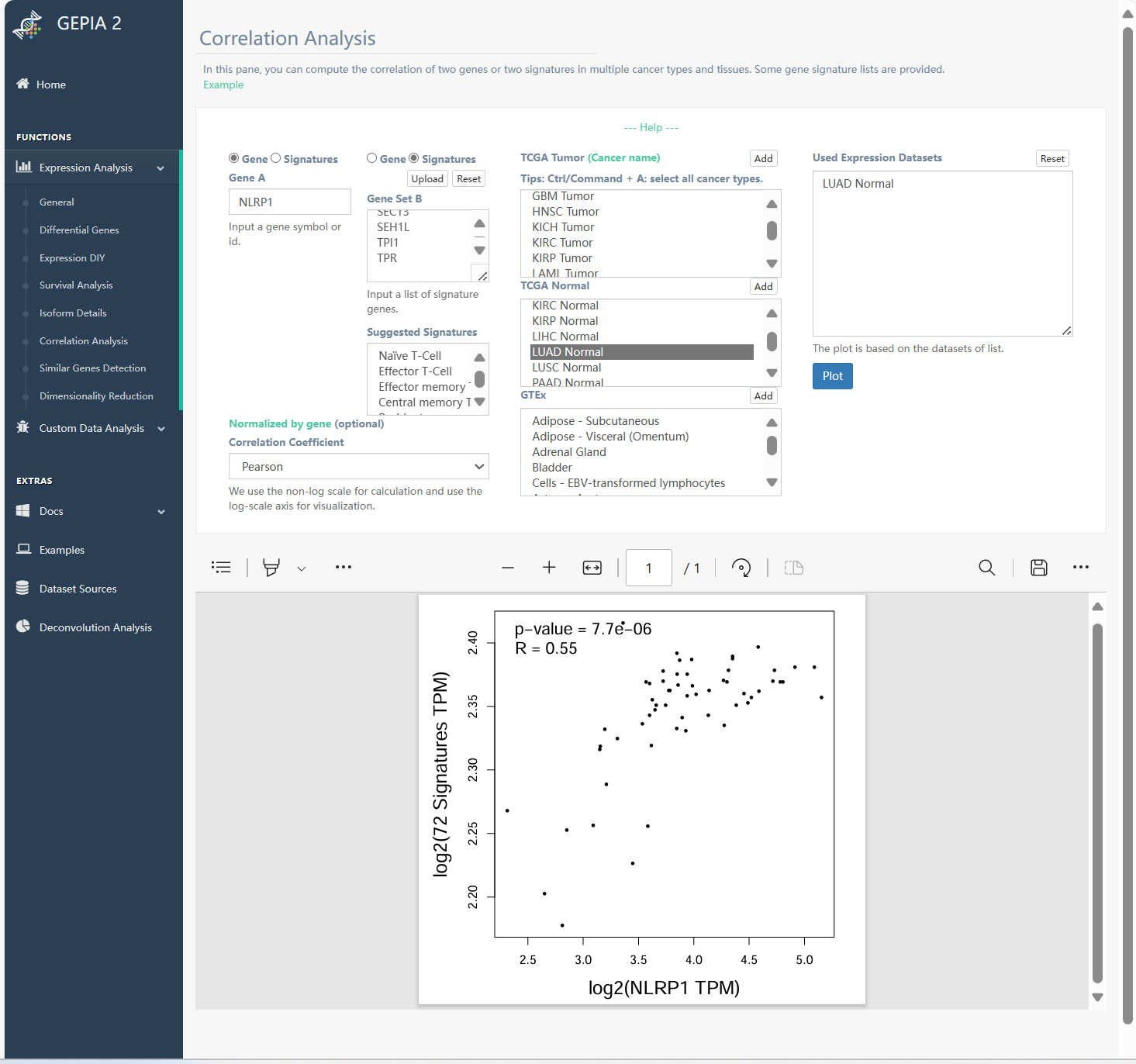


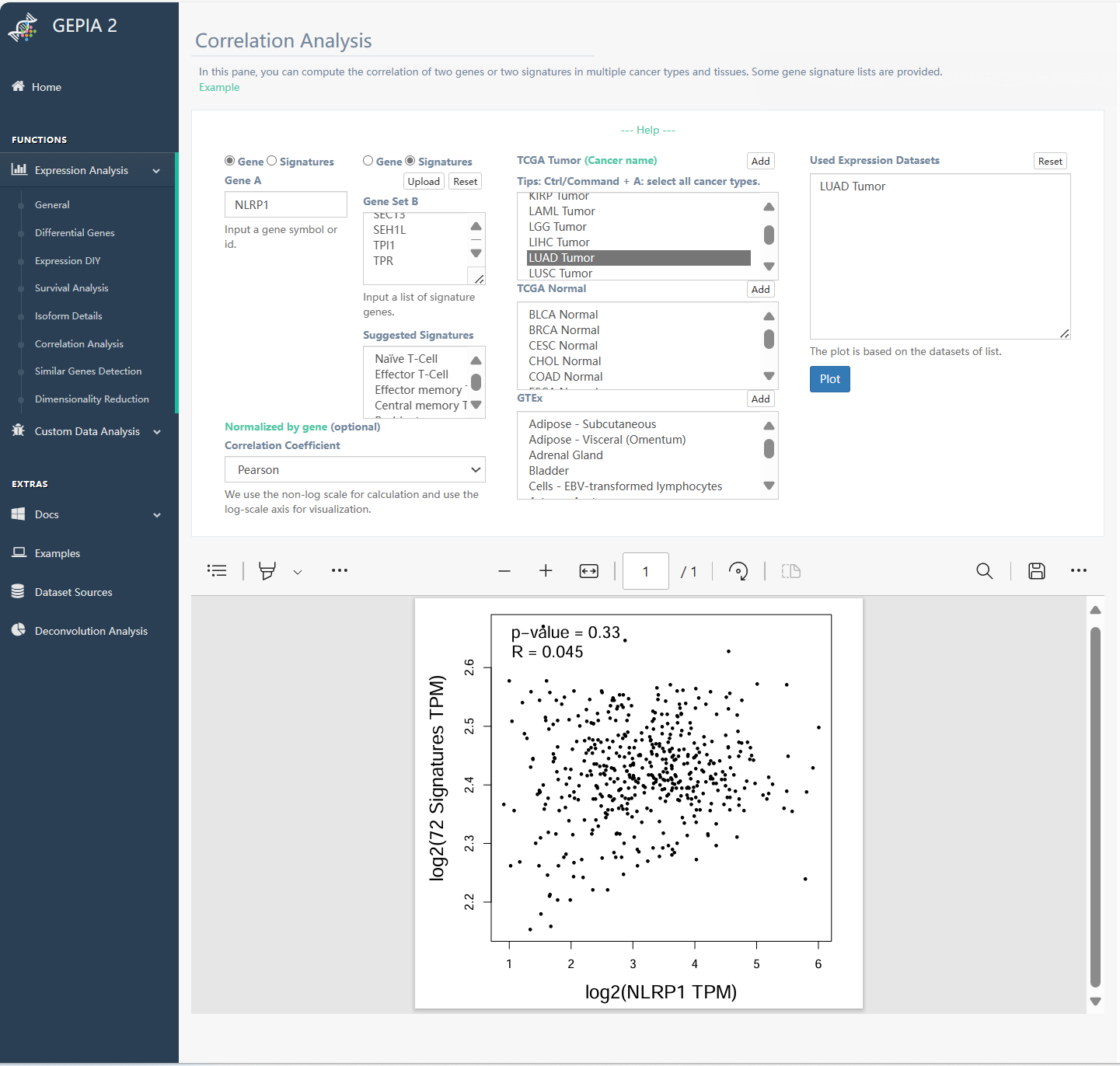


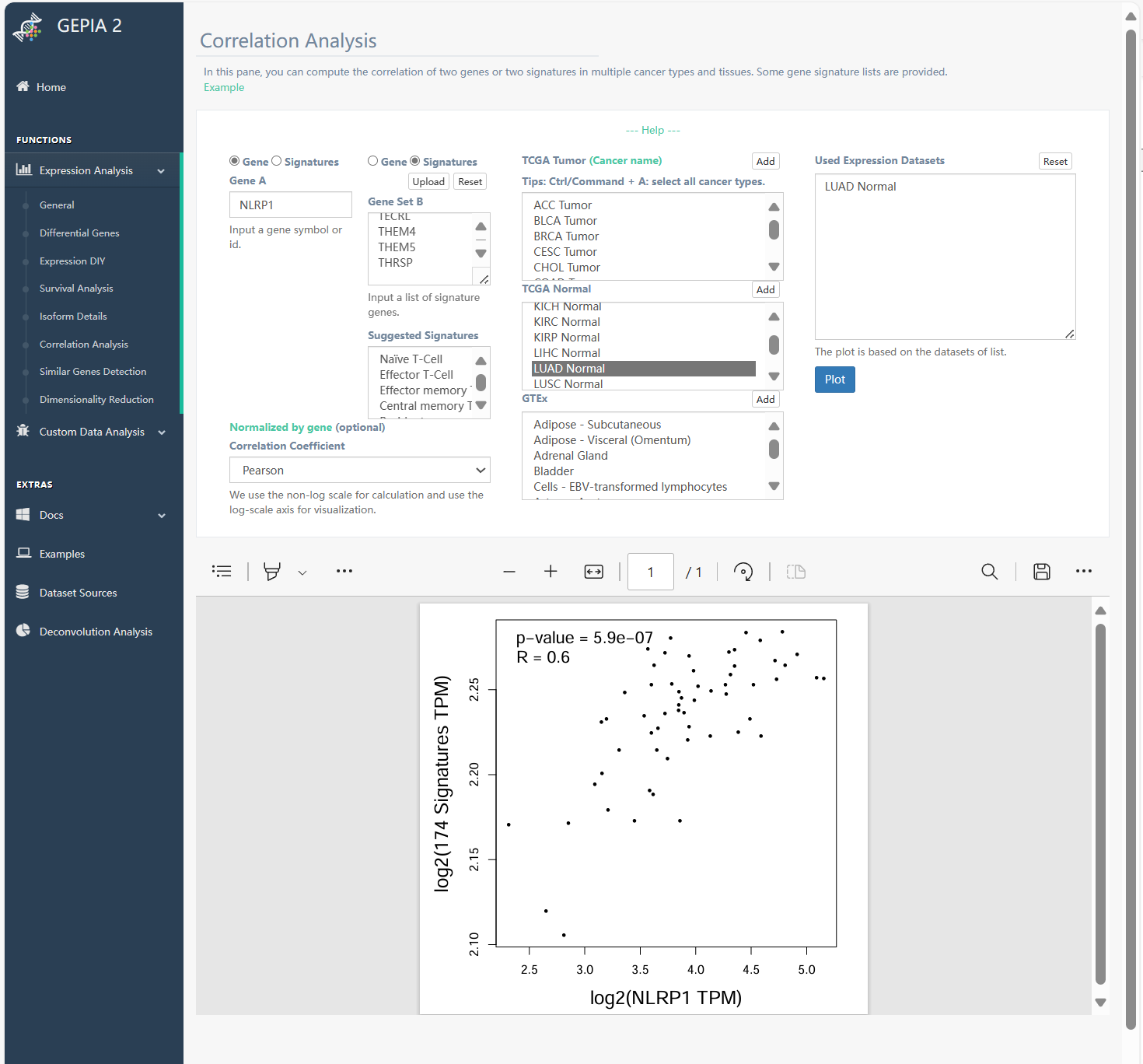


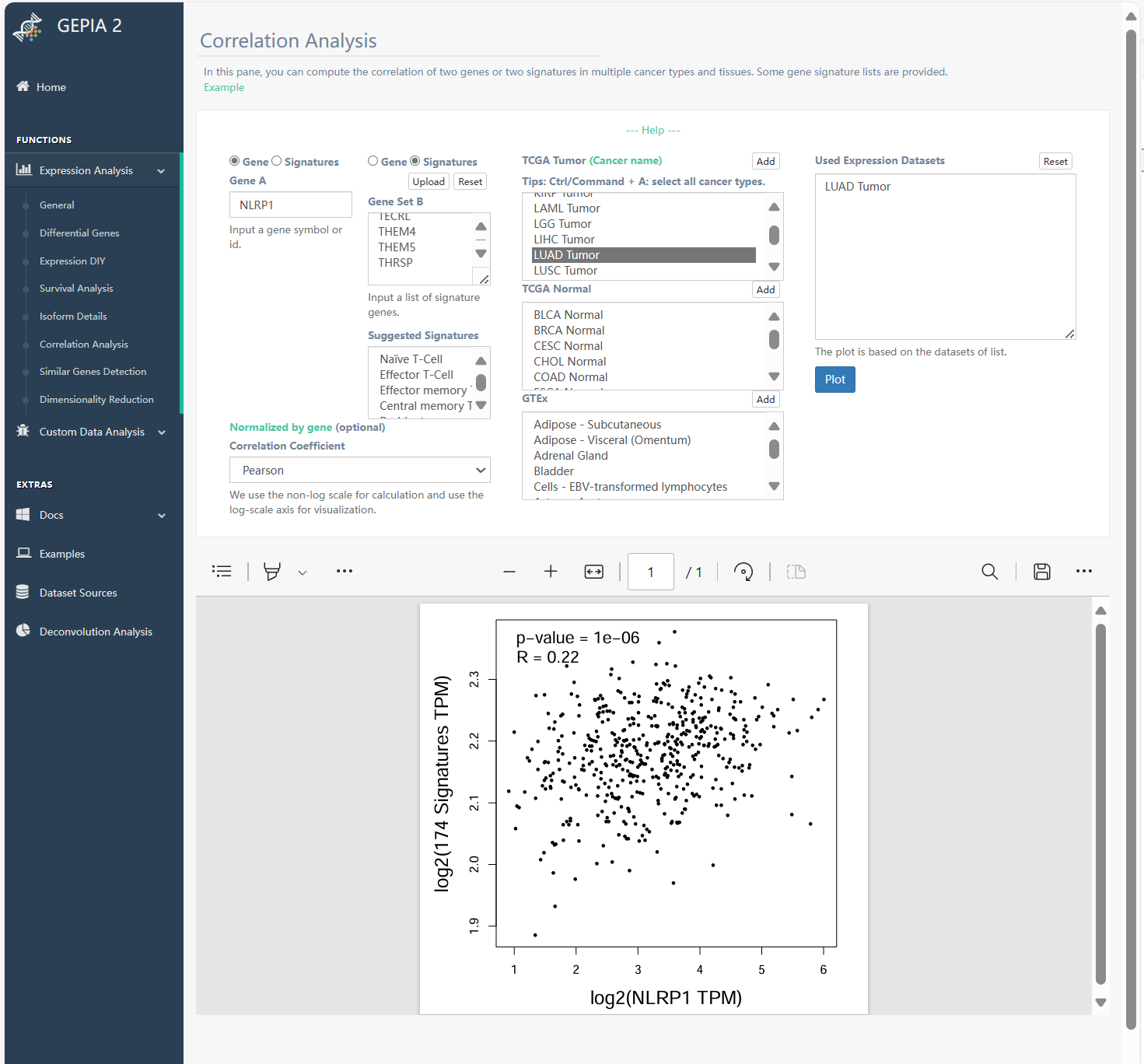


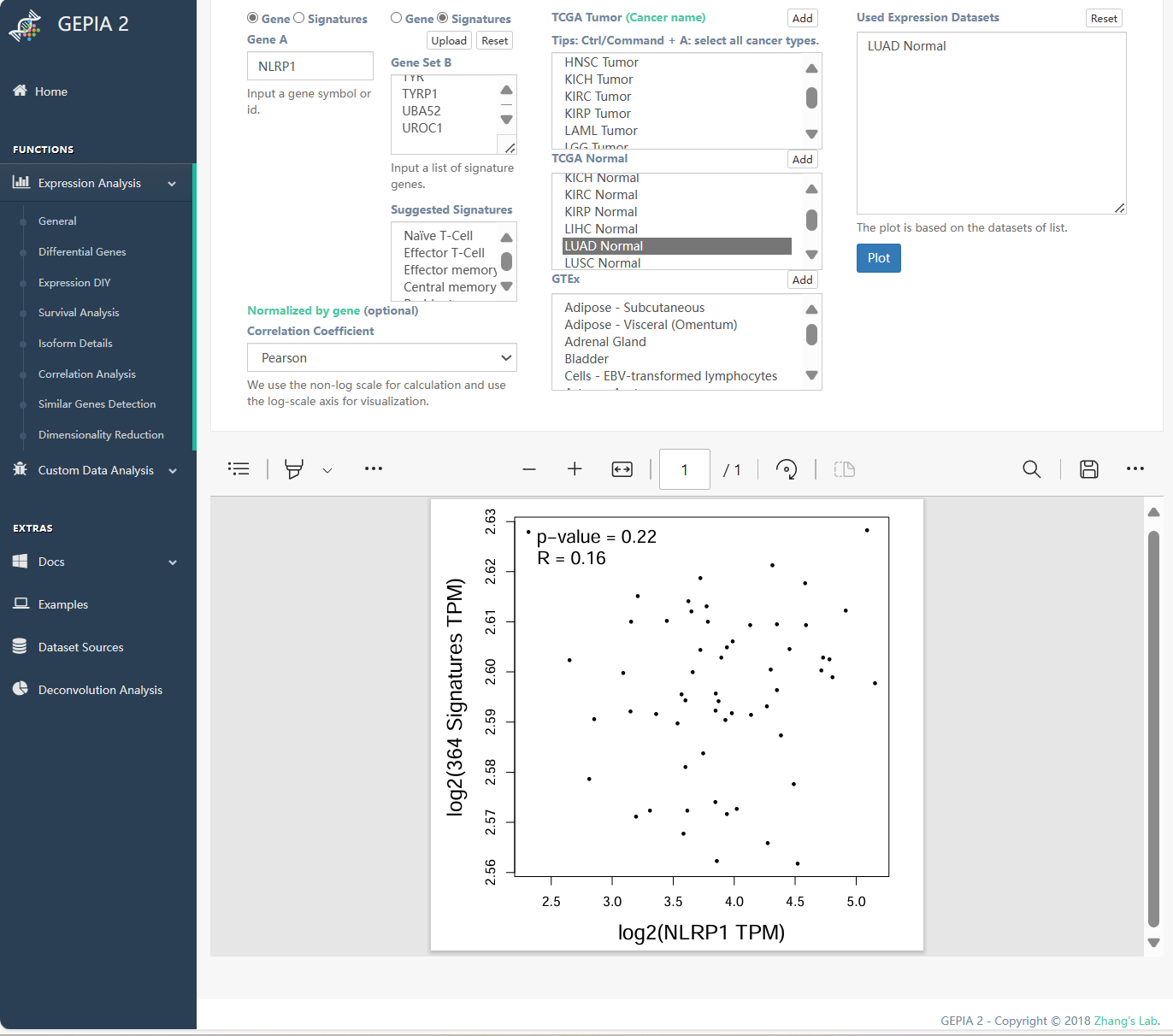


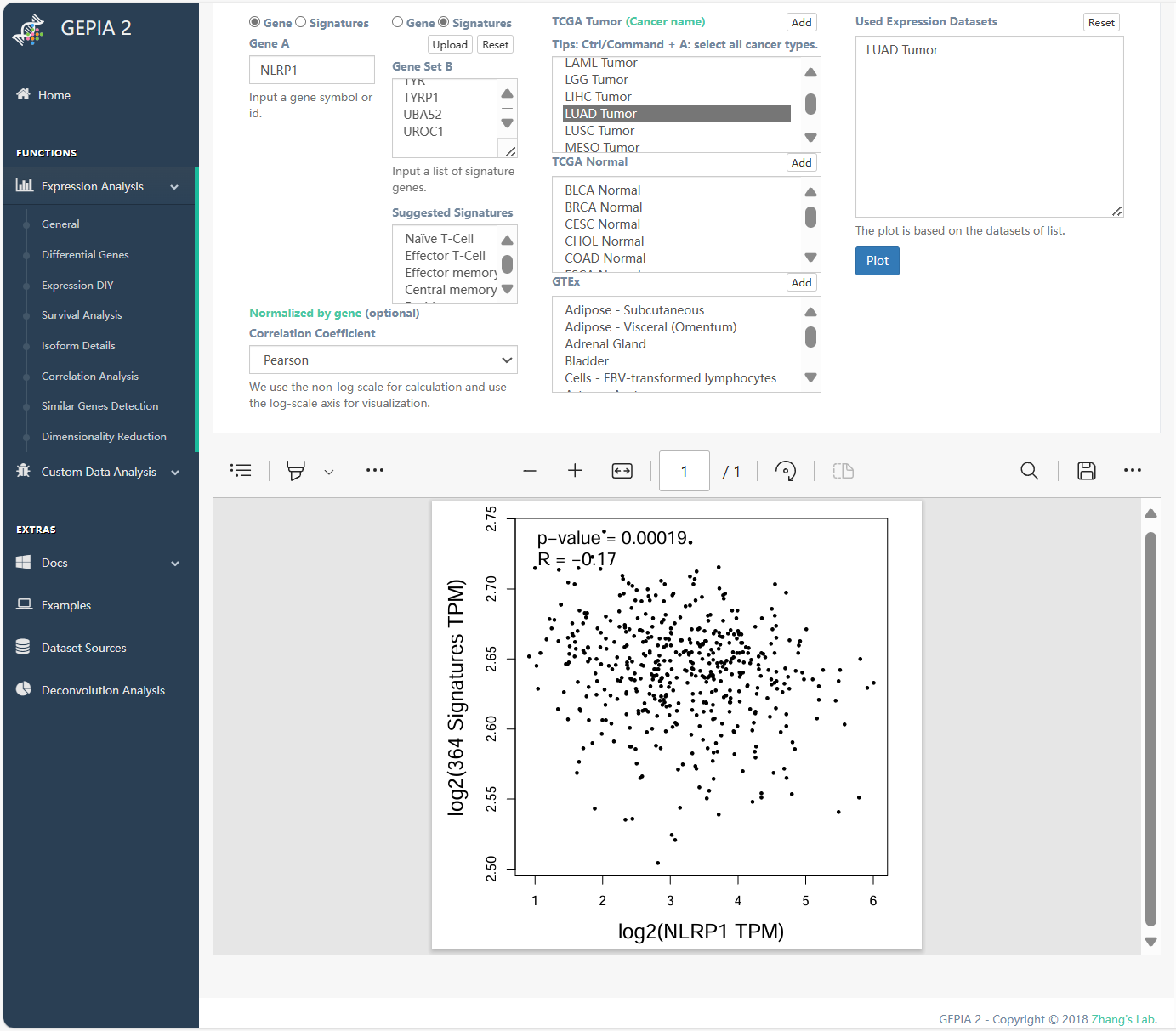


By following these steps, users can investigate the potential association between NLRP1 expression and tumor metabolic reprogramming in LUAD using the GEPIA2 platform. This analysis provides insights into the potential role of NLRP1 in modulating metabolism pathways associated with cancer progression.

**3.10 Connection Between NLRP1 Mutations and Tumor Prognosis (Figure 12, analyzed on May 12, 2024)**

**1. Access to mutation data of NLRP1 for Conducting NLRP1 Mutation Analysis through the cBioPortal Database:**

**1.1** Access cBioPortal Database:

Visit the cBioPortal website: http://www.cbioportal.org/.

**1.2** Select Study Data:

Under the “Select Study Data” section, choose “TCGA PanCancer Atlas Studies” from the “Quick Select” menu. This dataset encompasses 34 cancer types and 10,528 total samples.

**1.3** Choose Genomic Profiles:

In the “Select Genomic Profiles” section, select these options: “Mutations”, “Structural Variant”, “Putative copy-number alterations from GISTIC”

**1.4** Enter Gene for Analysis:

Input “NLRP1” in the “Enter Genes” field to specify NLRP1 for mutation analysis.

**1.5** Conduct the Analysis:

Click on the analysis button to initiate NLRP1 mutation analysis across different cancer types.


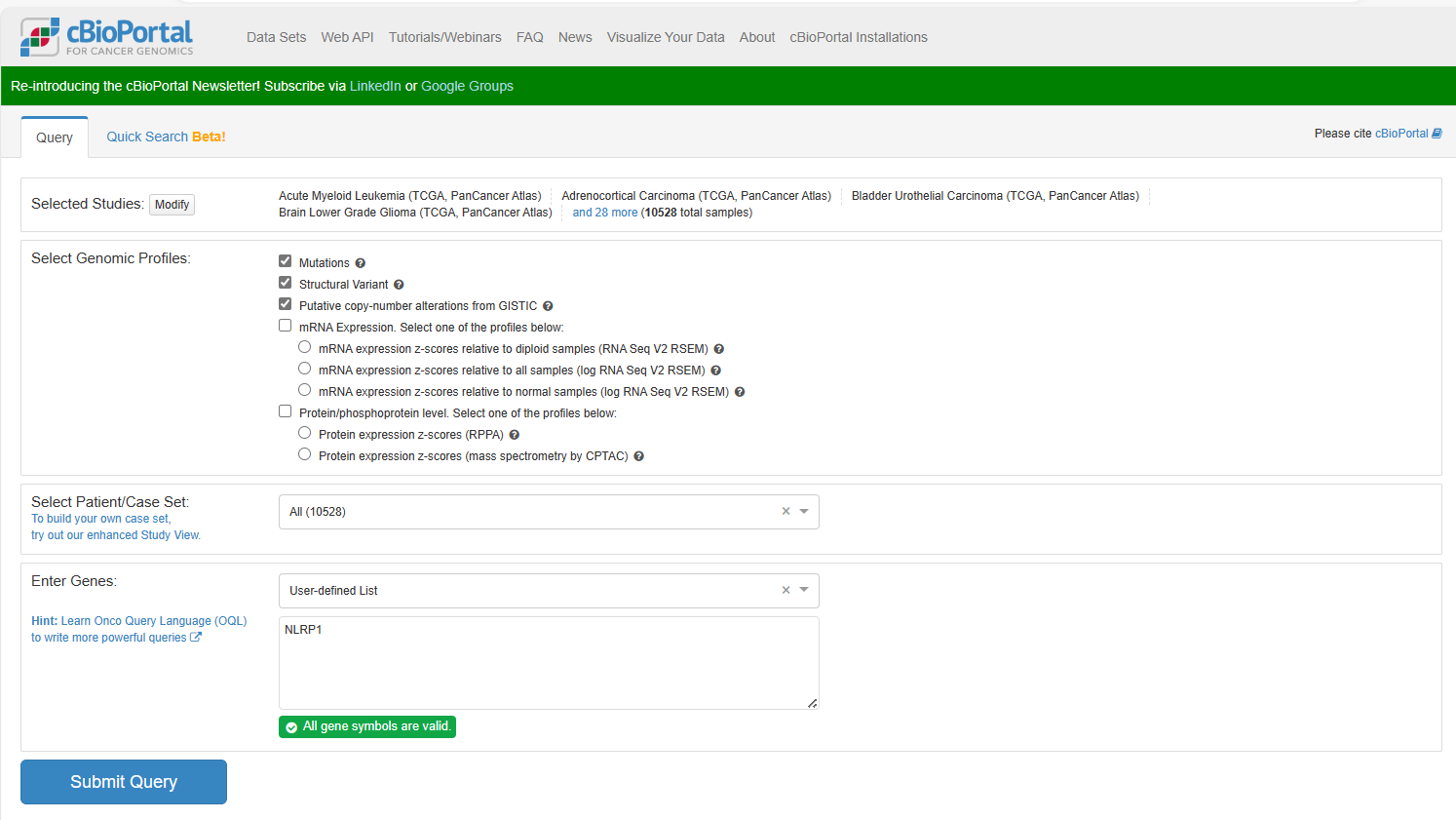


**2. Identifying the Highest Frequency of NLRP1 Alterations Across Various Cancer Types**

**2.1** Access the “Cancer Types Summary” Module:

Within the cBioPortal interface, locate and select the “Cancer Types Summary” module.

**2.2** Review NLRP1 Alterations:

Use the “Cancer Types Summary” module to analyze the frequency and distribution of NLRP1 alterations across different cancer types.

**2.3** Identify Highest Frequency:

Identify the cancer types with the highest frequency of NLRP1 alterations. This information provides insights into the prevalence of NLRP1 mutations in specific cancer contexts.

**2.4** Analyze the Results:

Review the summary to understand the significance of NLRP1 alterations in different cancer types and their potential implications for cancer biology and therapy (**Figure 12A** (main body manuscript)).


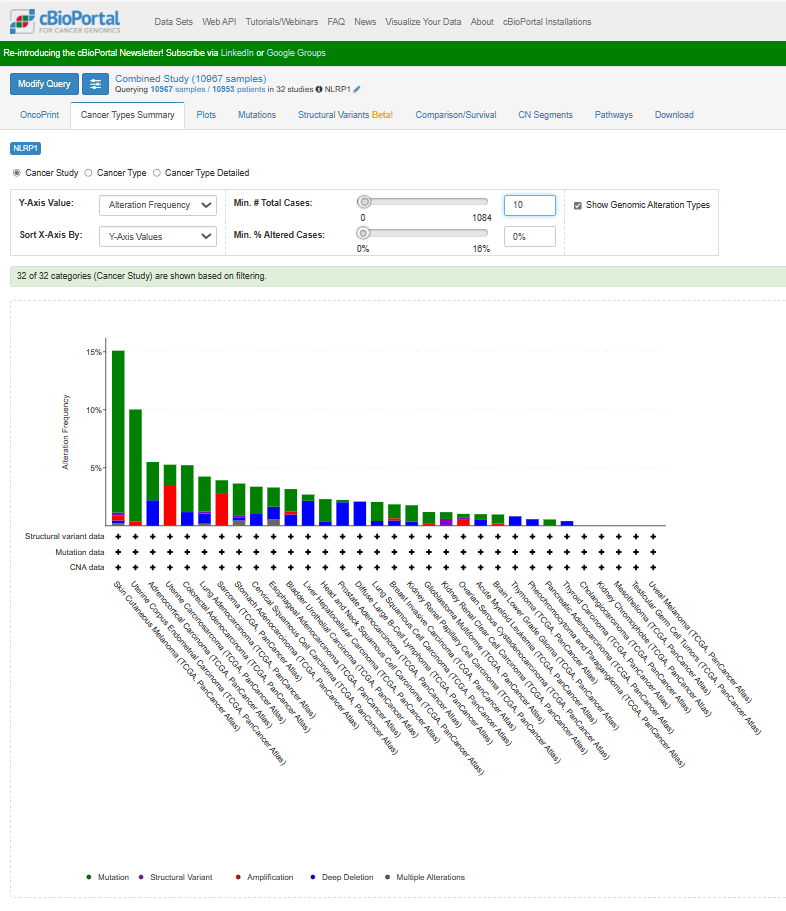


By following these steps, users can effectively identify the cancer types with the highest frequency of NLRP1 alterations using the cBioPortal “Cancer Types Summary” module. This analysis aids in understanding the prevalence and relevance of NLRP1 mutations across various cancer types.

**3. Exploring Detailed NLRP1 Mutation Information**

**3.1** Access the “Mutations” Module:

Click on the “Mutations” module within the cBioPortal interface.

**3.2** Review Mutation Details:

Explore detailed information such as the type of alterations, mutated sites, and mutation cases associated with NLRP1.

**3.3** Analyze Mutation Data:

Analyze the mutation data to understand the specific types of alterations (e.g., missense mutations, deletions, insertions) occurring in NLRP1 across different cancer types.

**3.4** Examine Mutated Sites:

Examine the mutated sites within the NLRP1 gene to identify regions that are frequently altered in cancer.

**3.5** Review Mutation Cases:

Review mutation cases to understand the distribution of NLRP1 mutations among individual samples and their potential clinical relevance (**Figure 12B** (main body manuscript)).


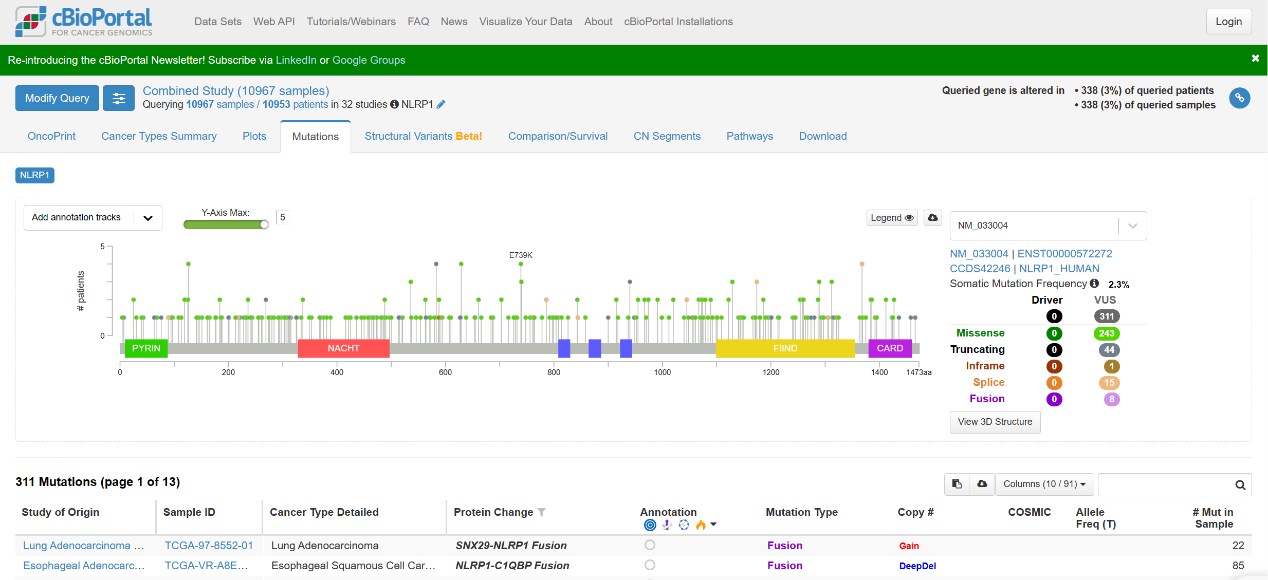


By clicking on the “Mutations” module, users can access detailed information about NLRP1 mutations, including the type of alterations, mutated sites, and mutation cases. This analysis provides valuable insights into the genetic landscape of NLRP1 in cancer and its potential implications for disease biology and therapy.

**4. Exploring Prognostic Differences in NLRP1 Mutations**

**4.1** Access the “Comparison/Survival” Module:

Within the cBioPortal interface, navigate to the “Comparison/Survival” module.

**4.2** Choose the “Survival” Function:

Select the “Survival” function within the “Comparison/Survival” module.

**4.3** Analyze the relationship Between NLRP1 Mutation and Prognosis:

Use the “Survival” function to analyze the relationship between NLRP1 mutation status and prognosis.

**4.4** Review Statistical Values:

Review statistical values reflecting disparities in survival types between the altered and unaltered groups within NLRP1 mutations. These values are presented in the left table of the page.

**4.5** View Survival Curves:

Click on “Overall” and “Progression Free” in the left table to obtain survival curves for overall and progression-free survival of the altered and unaltered groups within NLRP1 mutations (**Figure 12C-D** (main body manuscript)).


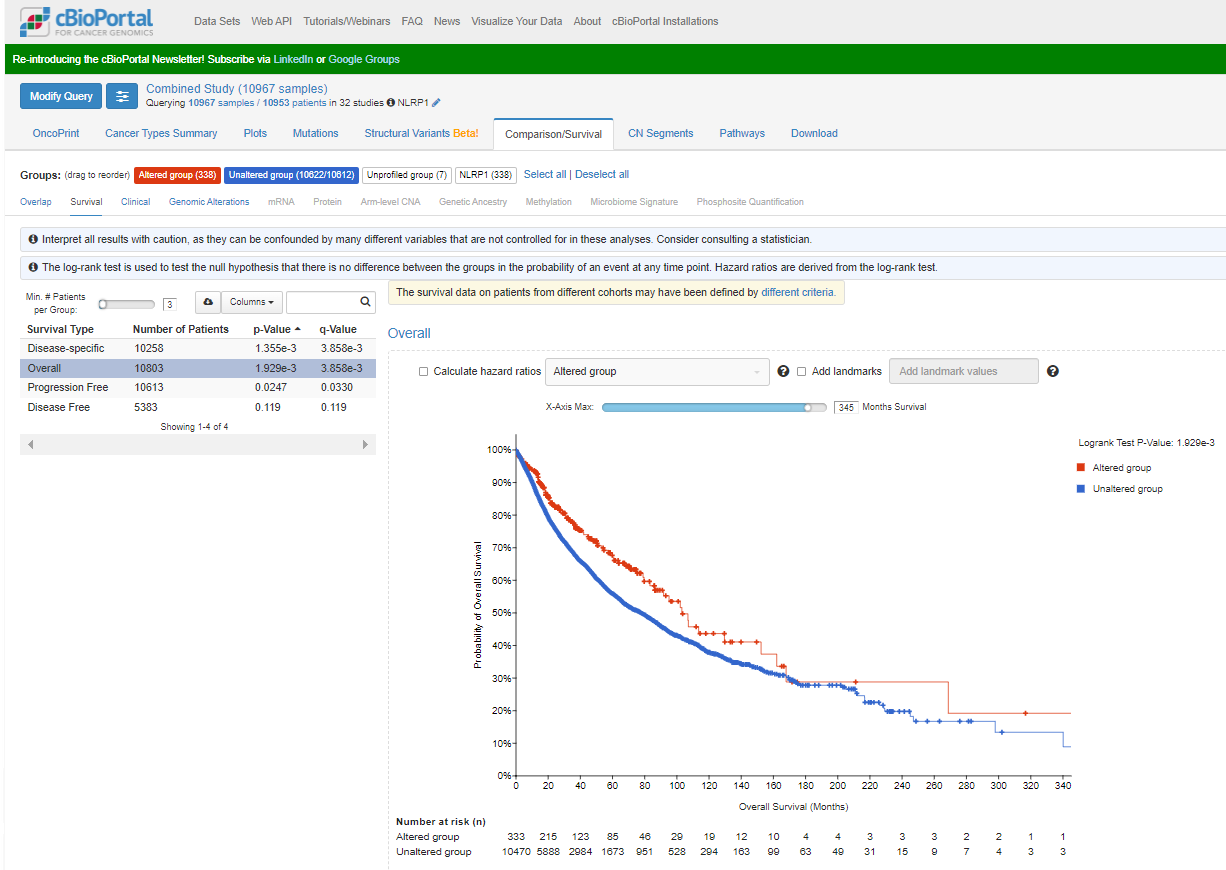

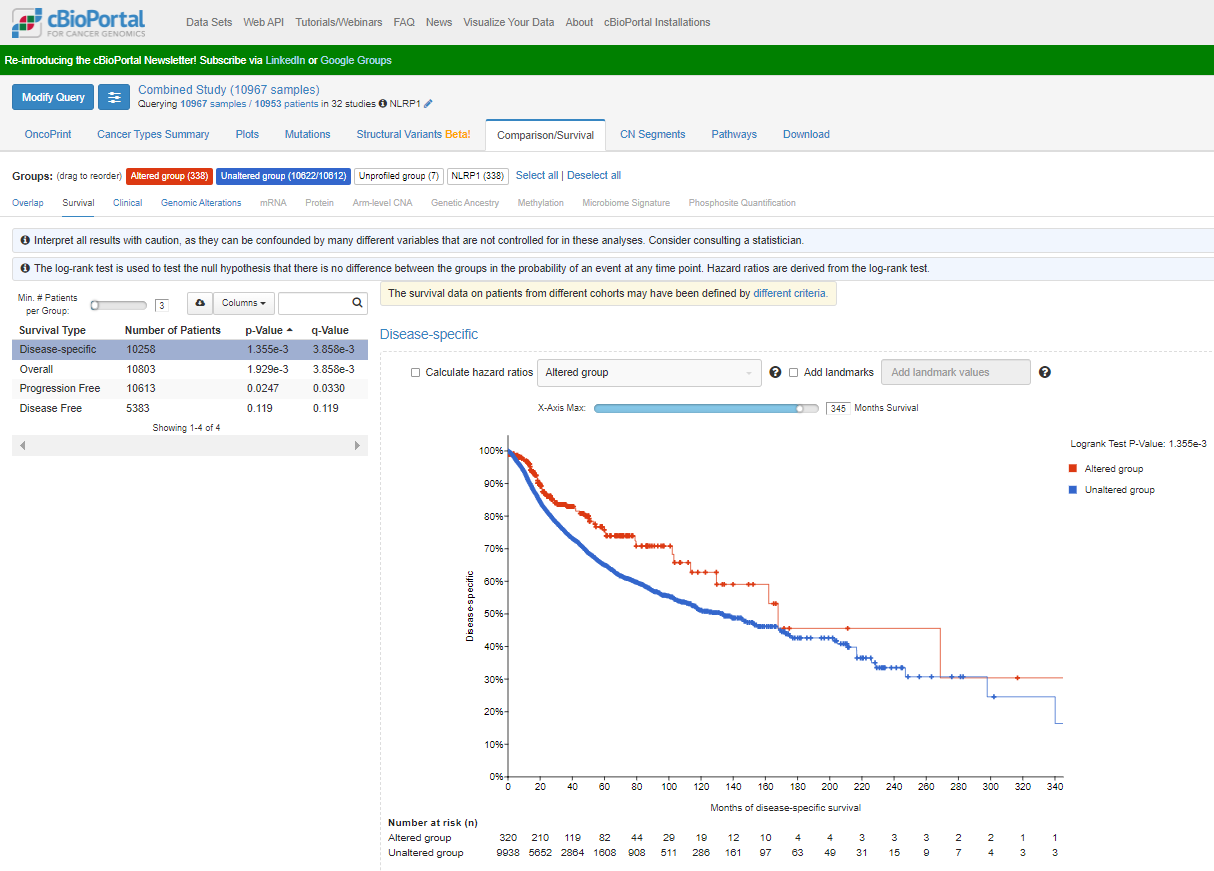


By following these steps, users can explore prognostic differences between the altered and unaltered groups within NLRP1 mutations using the “Survival” function of the “Comparison/Survival” module in the cBioPortal database. This analysis provides insights into the impact of NLRP1 mutations on patient prognosis in cancer.
